# APOBEC3B-Mediated Cytidine Deamination Is Required for Estrogen Receptor Action in Breast Cancer

**DOI:** 10.1016/j.celrep.2015.08.066

**Published:** 2015-09-24

**Authors:** Manikandan Periyasamy, Hetal Patel, Chun-Fui Lai, Van T.M. Nguyen, Ekaterina Nevedomskaya, Alison Harrod, Roslin Russell, Judit Remenyi, Anna Maria Ochocka, Ross S. Thomas, Frances Fuller-Pace, Balázs Győrffy, Carlos Caldas, Naveenan Navaratnam, Jason S. Carroll, Wilbert Zwart, R. Charles Coombes, Luca Magnani, Laki Buluwela, Simak Ali

**Affiliations:** 1 Department of Surgery and Cancer, Imperial College London, Hammersmith Hospital Campus, Du Cane Road, London W12 0NN, UK; 2 Department of Molecular Pathology, The Netherlands Cancer Institute, 1066 CX Amsterdam, the Netherlands; 3 Cancer Research UK, Cambridge Research Institute, Li Ka Shing Centre, Robinson Way, Cambridge CB2 0RE, UK; 4 Division of Cancer Research, University of Dundee, Ninewells Hospital and Medical School, Dundee DD1 9SY, UK; 5 MTA TTK Lendület Cancer Biomarker Research Group, Second Department of Pediatrics, Semmelweis University and MTA-SE Pediatrics and Nephrology Research Group, Budapest 1085, Hungary; 6 MRC Clinical Sciences Centre, Imperial College London, Hammersmith Hospital Campus, Du Cane Road, London W12 0NN, UK

## Abstract

Estrogen receptor α (ERα) is the key transcriptional driver in a large proportion of breast cancers. We report that APOBEC3B (A3B) is required for regulation of gene expression by ER and acts by causing C-to-U deamination at ER binding regions. We show that these C-to-U changes lead to the generation of DNA strand breaks through activation of base excision repair (BER) and to repair by non-homologous end-joining (NHEJ) pathways. We provide evidence that transient cytidine deamination by A3B aids chromatin modification and remodelling at the regulatory regions of ER target genes that promotes their expression. A3B expression is associated with poor patient survival in ER+ breast cancer, reinforcing the physiological significance of A3B for ER action.

## Highlights

• APOBEC3B is associated with poor survival in ER+ breast cancer patients
• APOBEC3B controls breast cancer cell growth by promoting ER transcriptional activity
• APOBEC3B can cause C-to-U mutations at ER target genes, to activate DNA repair
• Repair of APOBEC3B-induced lesions allows chromatin remodelling that stimulates gene expression

## Graphical Abstract

**Figure:**
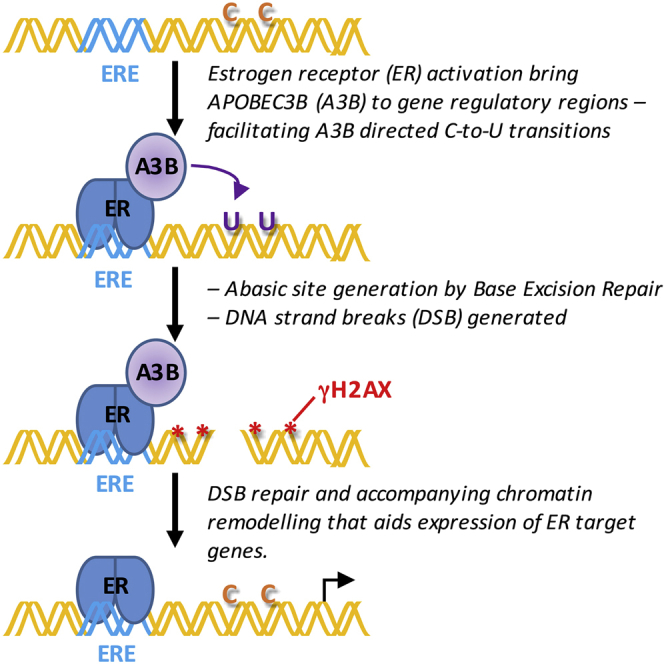

## Teaser

Periyasamy et al. show that APOBEC3B is required for the regulation of gene expression by the estrogen receptor in breast cancer cells. They report APOBEC3B can promote cytidine deamination at gene regulatory regions, with consequent repair providing a mechanism for chromatin remodelling that facilitates gene expression.

## Introduction

Estrogens play a central role in promoting breast cancer development (Ali and Coombes, 2002) and are important in uterine and ovarian cancer (O’Donnell et al., 2005; Shang, 2006). Two closely related nuclear receptors, estrogen receptor α (ERα, herein referred to as ER) and ERβ, mediate estrogen actions (Dahlman-Wright et al., 2006). ERα is dominant in breast cancer; 70% of breast cancers express ERα, and therapies to inhibit its activity have transformed breast cancer treatment. However, many patients develop resistance, with few treatment options being available for endocrine-therapy-resistant breast cancer (Osborne and Schiff, 2011).

Gene expression profiling and approaches for genome-wide identification of ER binding regions have allowed the identification of direct ER targets in breast cancer cells and highlight the importance of pioneer factors, particularly FOXA1 and GATA3 in directing ER by promoting chromatin accessibility and long-range chromatin interactions (Magnani et al., 2011; Ross-Innes et al., 2012). Critical for transcription regulation by ER is the ordered recruitment of a multitude of transcriptional co-regulator complexes with enzymatic activities for histone modification and chromatin remodeling (Métivier et al., 2006), which promote short- and long-range protein-DNA and protein-protein interactions between enhancer regions and gene promoters, to facilitate expression of ER target genes that drive breast cancer cell proliferation. The processes of transcription and DNA repair are intimately linked, as defined most obviously for the basal transcription factor TFIIH, which is essential for transcription initiation by RNA polymerase II (PolII), but is also required for the transcription-coupled nucleotide excision repair (Compe and Egly, 2012; Kamileri et al., 2012). Other DNA repair pathways also aid transcription (Fong et al., 2013) by promoting active DNA demethylation (Bhutani et al., 2011; Nabel and Kohli, 2011), enhancer RNA (eRNA) synthesis (Puc et al., 2015), and chromatin remodeling (Ju et al., 2006; Perillo et al., 2008), through processes that can involve the generation of single- and double-strand DNA breaks at enhancer regions.

The AID/APOBEC genes comprise a family of enzymes that mutate RNA or DNA by deaminating cytidine to uridine. Among their functions are RNA editing of the apolipoprotein B pre-mRNA by APOBEC1 and generation of antibody diversity by class-switch recombination and somatic hypermutation through DNA editing by AID (Conticello, 2008). In primates, there are seven closely related APOBEC3 genes, some of which function in retroviral restriction by promoting “hypermutation” in viral genomes. These functions do not readily explain the potential roles of APOBEC3 genes in non-immune system tissues, including breast, lung, cervix, bladder, and ovary, or the overexpression of some members of the family in cancers from these tissues (Burns et al., 2013a, 2013b; Leonard et al., 2013), although a potential role in the repair of DNA double-strand breaks (DSBs), resulting in resistance of lymphoma cells to ionizing radiation, has been ascribed to APOBEC3G (Nowarski et al., 2012). Interestingly, ectopic expression of APOBEC3A (A3A) and A3B can promote mutagenesis in cancer cells (Burns et al., 2013a; Landry et al., 2011; Taylor et al., 2013).

Cancer genomes are marked by an accretion of somatic mutations. Recent whole-genome sequencing of breast cancer has yielded genome-wide mutational signatures, one of which is consistent with the DNA mutation profiles associated with cytidine deamination by APOBEC3 genes (Alexandrov et al., 2013; Nik-Zainal et al., 2012). Similar mutational signatures have been described in ovarian, bladder, cervical, head and neck, and lung cancer (Burns et al., 2013a, 2013b; de Bruin et al., 2014; Leonard et al., 2013; Roberts et al., 2013). A3B expression is frequently elevated in breast and other cancers that feature mutational landscapes consistent with cytidine deaminase activity (Burns et al., 2013a, 2013b). This, together with the demonstration that ectopic A3B expression can promote C-to-T mutations in breast cancer cells, has led to a proposed model in which A3B overexpression in breast cancer could aid tumor initiation and progression by driving somatic mutations in cancer. However, breast cancers from patients featuring a germline copy-number polymorphism involving A3A and A3B, in which A3B is effectively deleted, carry a greater burden of mutations associated with the APOBEC-dependent signature than those in which the A3A and A3B genes are intact (Nik-Zainal et al., 2014), bringing into question the importance of A3B in this process.

To investigate the potential role of A3B in breast cancer, we analyzed its expression in breast cancer. Surprisingly, A3B expression was associated with poor patient survival in ER+ breast cancer, but not in ER− breast cancer, despite the fact that A3B expression was higher in ER− breast cancer than in ER+ breast cancer, thus implicating A3B in ER action. Here, we provide evidence for a molecular mechanism in which A3B causes local and transient C-to-U transitions at ER enhancers, leading to activation of base excision repair (BER) and non-homologous end-joining (NHEJ) pathways, which in turn promote chromatin modification and remodeling, to drive expression of ER target genes.

## Results

### A3B Expression Is Associated with Poor Prognosis in ER+ Breast Cancer

**Figure 1:**
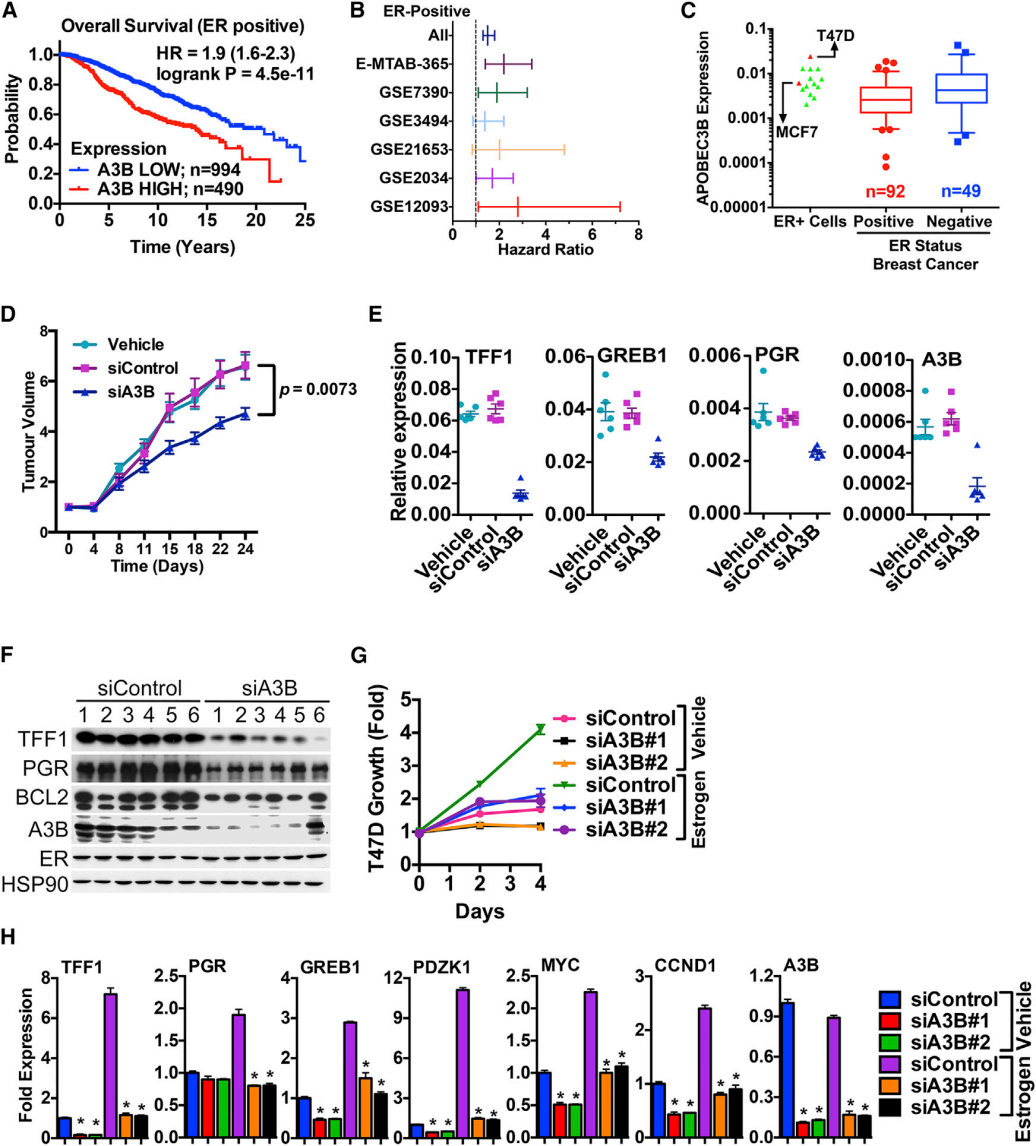
A3B Regulates ER Activity to Promote Breast Cancer Cell Growth (A) Kaplan-Meier plots of breast cancer survival for ER+ patients from METABRIC, according to A3B expression. (B) Forest plot analysis of ER+ breast cancers for A3B expression. Affymetrix microarray datasets that included >100 ER+ breast cancers were used in the analysis: E-MTAB-365 (Guedj et al., 2012), GSE7390 (Desmedt et al., 2007), GSE3494 (Miller et al., 2005), GSE21653 (Sabatier et al., 2011), GSE2034 (Wang et al., 2005), and GSE12093 (Zhang et al., 2009). Hazard ratios and 95% confidence intervals are plotted on the x axis. (C) Real-time RT-PCR was carried out using RNA prepared from ER+ (n = 92) and ER− (n = 49) breast cancers. Also shown are the expression profiles for ER+ breast cancer cell lines, with MCF7 and T47D expression highlighted. (D) MCF7 tumors were treated weekly with vehicle (n = 6), control siRNA (n = 6), or A3B siRNA (n = 6). Mean tumor volumes are plotted ± SEM. RNA and protein were prepared from tumors at the end of the experiment. (E) Real-time RT-PCR relative to GAPDH levels (n = 3; p < 0.0001) for tumors. (F) Immunoblotting of protein lysates (20 μg) tumors is shown. (G) Hormone-depleted T47D cells transfected with A3B siRNAs were assessed for growth (n = 4). (H) mRNA levels were determined following transfection of hormone-depleted T47D cells with A3B siRNAs. mRNA expression is shown relative to the expression for the siControl samples (n = 3). Statistical significance within each treatment group for each A3B siRNA relative to siControl is denoted by asterisks (p < 0.001).

AID/APOBECs function in retroviral restriction and in the case of AID, in class-switch recombination and somatic hypermutation to generate antibody diversity by causing C-to-T mutations (Conticello, 2008), functions that do not explain the high-level expression of A3B in many cancers (Burns et al., 2013a, 2013b). Toward defining the role of A3B in breast cancer subtypes, we determined the relationship between A3B levels and patient outcome. Analysis of the METABRIC (Curtis et al., 2012) series of 2,000 breast cancer patients revealed that high A3B expression is associated with poor survival (hazard ratio [HR] = 1.5, p = 1 × 10^−11^) (Figures S1A and S1B). Interestingly, A3B expression was associated with poor outcome in ER+ (HR = 1.9; p = 4.5 × 10^−11^) (Figure 1A), but not in ER− (p = 0.18), breast cancer. A3B retained its significance (HR = 1.59, p = 1.27 × 10^−6^) in multivariate analysis of ER+ breast cancer (Figure S1C). There was no association between expression of other APOBECs and poor outcome in ER+ or ER− breast cancer in the METABRIC dataset (data not shown). We extended this analysis to other gene expression microarray datasets. Kaplan-Meier plot analysis of Affymetrix microarray datasets similarly showed that high A3B expression is associated with poor outcome for ER+, but not ER−, breast cancer (Figures S1D–S1F). Forest-plot analysis for relapse-free survival further confirmed the importance of A3B in ER+ breast cancer (Figure 1B). Real-time RT-PCR for 151 breast cancers again confirmed that A3B is expressed in ER+ and ER− breast cancer, as well as in the majority of breast cancer cell lines examined (Figures 1C and S1G–S1R), in agreement with previous findings (Burns et al., 2013a).

### A3B Regulates the Growth of ER+ Breast Cancer Cells

Given the association between A3B expression and poor patient survival in ER+ breast cancer, we wondered whether A3B regulates the growth of ER+ breast cancer cells. To investigate this, we treated tumor xenografts of the ER+ and estrogen-regulated MCF7 breast cancer cell line, which expresses moderate levels of A3B, with A3B small interfering RNA (siRNA). A3B knockdown markedly inhibited MCF7 tumor growth (Figure 1D), demonstrating that A3B is required for MCF7 tumor growth in vivo. Remarkably, ER target gene expression was greatly reduced in these tumors (Figures 1E and 1F), suggesting that A3B impacts directly on ER function. Indeed, transfection of MCF7 cells in culture with two independent A3B siRNAs inhibited estrogen-stimulated growth, accompanied by reduced expression of ER regulated genes (Figures S2A–S2C). Inhibition of estrogen-regulated growth and estrogen-responsive gene expression was confirmed in a second ER+ cell line (T47D) (Figures 1G and 1H). SkBr3 cells, which are null for A3B (Komatsu et al., 2008), were not affected by A3B siRNA (Figures S1I and S2D). Growth of the A3B+/ER− MDA-MB-231 cells was also unaffected by A3B siRNA (Figure S2E). Furthermore, inhibition of ER target gene expression by a siRNA targeting the A3B 3′-UTR was rescued by transfection of an A3B expression plasmid that lacks the 3′ UTR. However, siRNAs targeting the A3B coding region knocked down endogenous and ectopic A3B and inhibited ER-regulated genes (Figures S2F and S2G). Taken together, these results demonstrate A3B specificity of the siRNAs and show that A3B regulates growth and expression of estrogen-responsive genes in breast cancer cells.

### A3B Is Recruited Globally to ER Binding Regions, and This Requires Its Interaction with the ER

**Figure 2:**
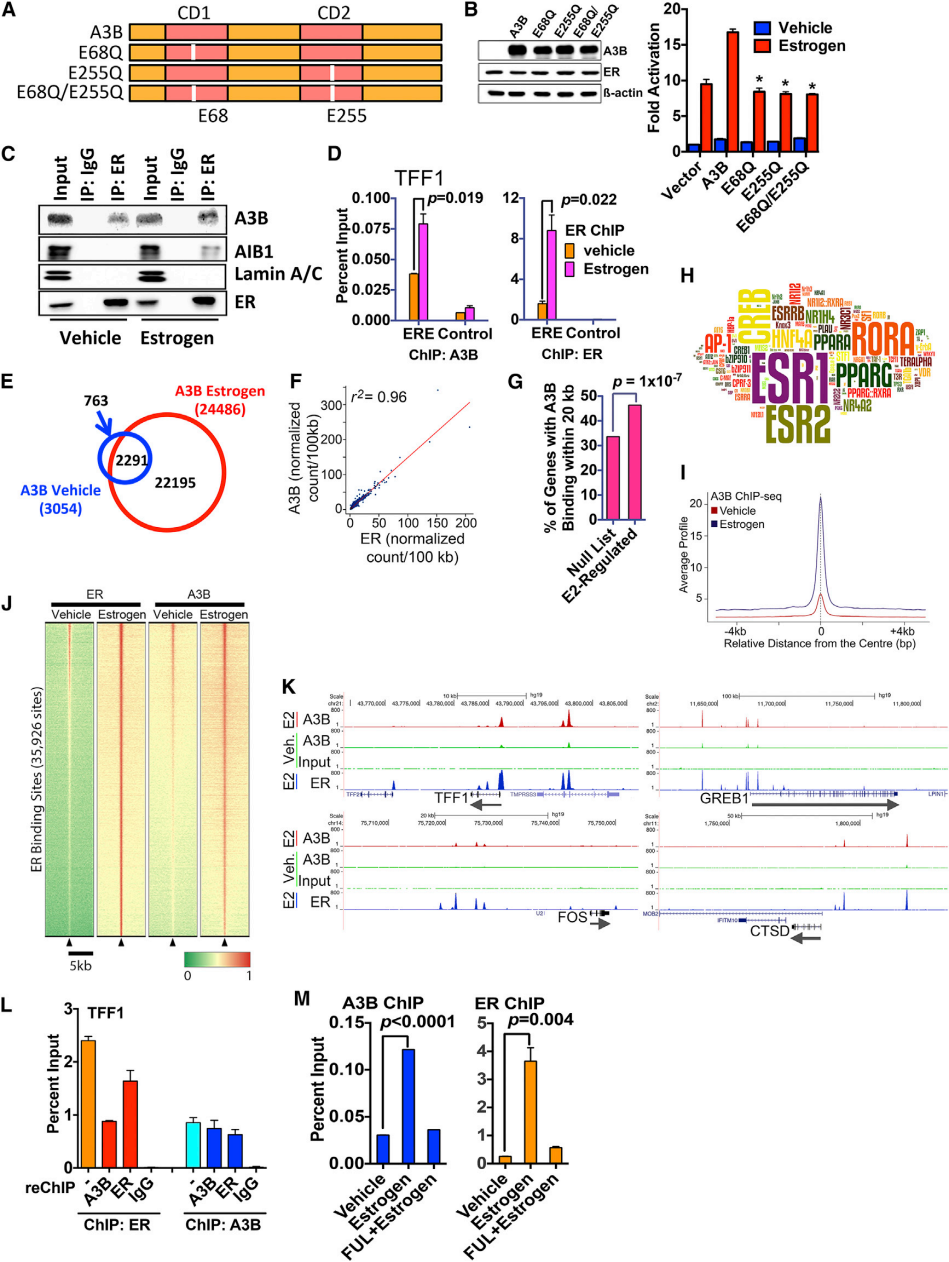
A3B Interacts with ER and Is Recruited to ER Binding Regions (A) Schematic representation of A3B. Highlighted are the catalytic site mutations used here. (B) COS-1 cells cultured in hormone-free medium were transfected with an estrogen-responsive luciferase reporter gene, ER and A3B. Estrogen (10 nM) was added for 20 hr. Data are shown as fold activation, relative to reporter activity for vehicle treatment, following co-transfection of ER without A3B (vector control) (n = 3; ^∗^ = p < 0.05). Immunoblotting for ER and A3B following transfection of COS-1 cells is also shown. (C) Hormone-depleted MCF7 cells treated with estrogen were immunoprecipitated with an ER antibody. Input represents 10% of lysate used in the immunoprecipitations. (D) MCF7 cells were treated with estrogen (10 nM, 45 min), followed by ChIP. Shown are the results of real-time PCR of ChIP DNA for the TFF1 gene promoter proximal ERE or a control region in the TFF1 gene to which ER binding is not observed (n = 3). (E) A3B ChIP-seq was carried out following treatment of MCF7 cells with 10 nM estrogen for 45 min. The Venn diagram shows A3B binding regions from the peak-calling analysis. (F) Genome-wide enrichment correlation analysis for A3B and ER raw signals demonstrates correlation (*r*^*2*^ = 0.96) between A3B and ER binding sites. A3B and ER ChIP-seq reads were normalized (wig file) and binned in windows of 100 kb where the average score was calculated. The data were then used to calculate genome-wide correlation using a Spearman’s correlation score. The score for each window is plotted and an interpolation line added to ease interpretation. (G) Analysis of A3B binding regions identified by ChIP-seq are significantly enriched in the proximity of estrogen responsive genes. (H) Analysis of the relative enrichment of transcription factor binding sites was used to generate a *Z* score, which is represented by the size of the motif in the word cloud. (I) Average signal intensity of A3B ChIP-seq binding events, centered on ER binding regions, shows increased recruitment of A3B to ER binding regions globally, upon estrogen treatment. Signal intensity is a normalized count of individual, non-redundant ChIP fragments at single ER binding sites identified by the peak-calling algorithm (MACS 1.4) (J) Heatmap showing clustered binding signal for A3B ± estrogen. The window represents ±2.5-kb regions from the center of the ER binding events. The color scale represents relative enrichment based on raw signal. (K) Representative genome browser snapshots show A3B binding regions and overlap with ER binding regions, shown on the same scale. (L) ChIP with ER or A3B antibodies (denoted by −) was followed by recovery of the chromatin complexes and reChIP with antibodies for A3B, ER, or mouse immunoglobulins (IgG control) (n = 3). (M) MCF7 cells were treated with fulvestrant for 24 hr, followed by addition of estrogen for 45 min (n = 3).

Next, we sought to determine whether A3B is required for the ER transcriptional response. In a reporter gene assay, A3B stimulated ER activity (Figures 2A and 2B). A3B contains two zinc coordinating cytidine deaminase activity (CDA) domains (Conticello, 2008). Substitution of glutamic acid residues at positions 68 or 255 to glutamine, which inhibits the cytidine deaminase activity in the N- and C-terminal domains of A3B, respectively, reduced stimulation of ER activity. Hence, both CDA domains are required for modulation of ER activity by A3B, echoing a previous report, which showed that both CDA domains are enzymatically active and contribute to C-to-T editing (Bogerd et al., 2007). A3B was co-immunoprecipitated with ER in MCF7 cells (Figure 2C), and chromatin immunoprecipitation (ChIP) assays showed that A3B is recruited, in an estrogen-dependent manner, to the ER binding regions in the TFF1 and GREB1 genes (Figures 2D and S3A). AIB1, which interacts with ER in an estrogen-dependent manner (Anzick et al., 1997), acts as a control.

ChIP sequencing (ChIP-seq), for A3B to define the global distribution of A3B on chromatin, identified 24,486 binding sites in MCF7 cells treated with estrogen (Figures 2E and S3B). A3B binding was primarily observed at intronic and gene-distal regions (Figure S3C), suggesting that A3B binding occurs in gene regulatory regions. ChIP-seq for ER has established that the great majority of ER binding events also map to sites within introns and at considerable distances upstream and downstream of transcribed regions (Welboren et al., 2009). Analysis of the raw reads demonstrated a remarkably close genomic co-localization of the binding sites for the two proteins (*r*^*2*^ = 0.96) (Figure 2F). A3B binding sites were significantly enriched in the vicinity of estrogen-responsive genes (Figure 2G) and were enriched in binding motifs for ER (Figure 2H; Table S1). Alignment of all ER binding sites showed that the majority of ER binding sites in MCF7 cells are bound by A3B and that estrogen treatment results in global stimulation of A3B recruitment to ER binding regions (Figures 2I and 2J), as evident from the genome browser snapshots for the ER target genes TFF1, GREB1, FOS, and CTSD (Figure 2K). These results provide a compelling argument for a mechanism of chromatin-based collaboration between A3B and ER, toward the global regulation of ER target genes in breast cancer.

Recovering the chromatin following ChIP for ER and performing ChIP with A3B antibody (ChIP/reChIP) showed that ER and A3B are present concurrently at the TFF1 ERE (Figure 2L). ChIP for A3B followed by reChIP for ER provided similar results. Treatment of MCF7 cells with the anti-estrogen fulvestrant (aka ICI182,780), which specifically promotes the downregulation of ER protein (McClelland et al., 1996), resulted in ER loss and lack of ER binding to chromatin in ChIP assays (Figures 2L and S3D). Fulvestrant did not affect A3B protein levels, but A3B recruitment to the TFF1 and PDZK1 ER binding regions was nevertheless prevented. By contrast, siRNA-mediated A3B knockdown did not affect ER recruitment (Figure S3E). Thus, although A3B interacts with ER in a ligand-independent manner, its recruitment to chromatin is estrogen dependent by virtue of estrogen-stimulated recruitment of ER to chromatin. Furthermore, given that A3B is not required for ER recruitment to DNA, A3B is unlikely to act as a pioneer factor.

### A3B Promotes Cytidine Deamination to Generate C-to-U Transitions at ER Binding Regions in Breast Cancer Cells

**Figure 3:**
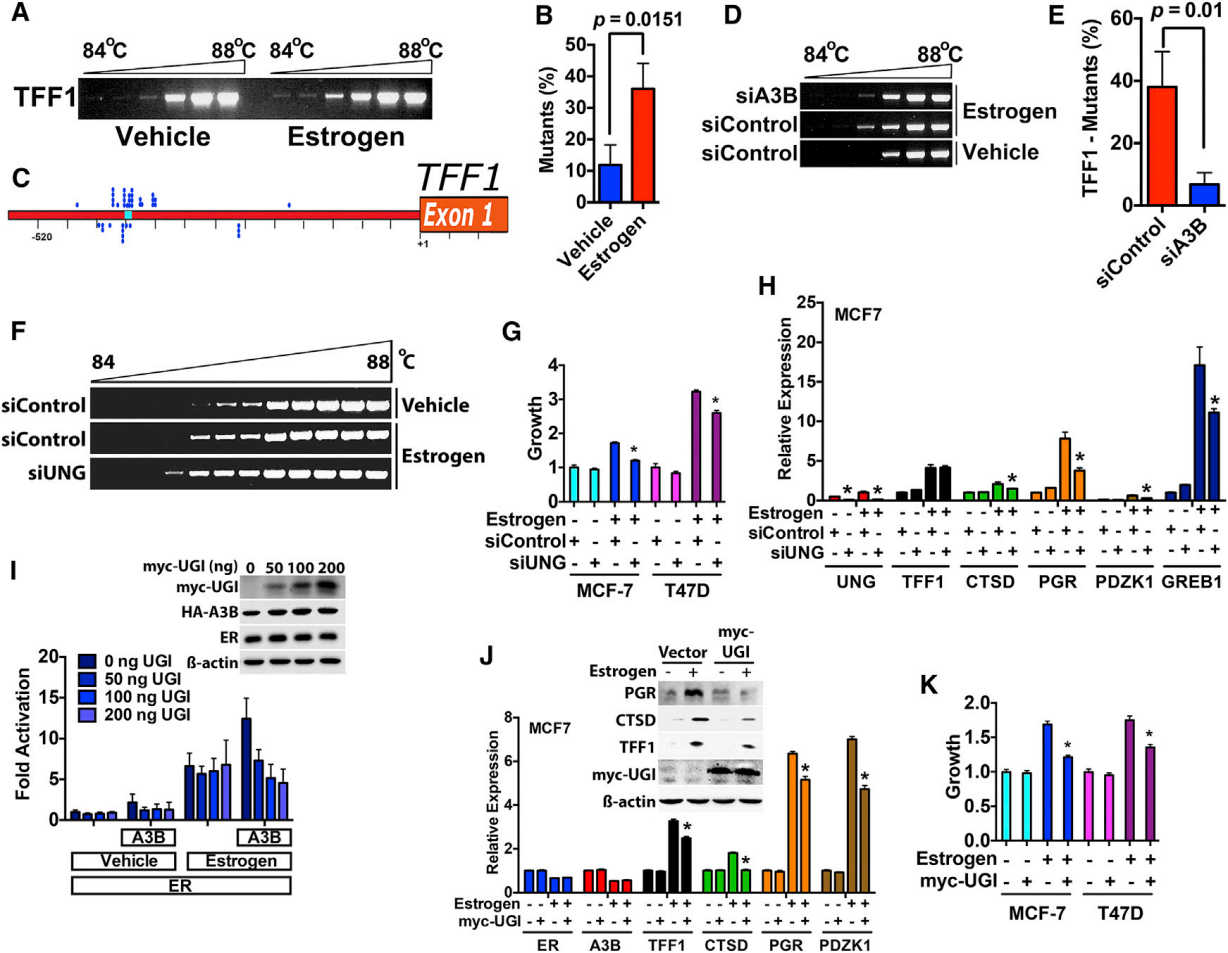
Estrogen Treatment Induces A3B-Dependent C-to-U Transitions at the ER and A3B Binding Region in the TFF1 Gene (A) Genomic DNA was prepared from MCF7 cells treated with 10 nM estrogen for 45 min. Agarose gel analysis of 3D-PCR for the TFF1 promoter region is shown. (B) Mutation analysis of 3D-PCR amplicons from vehicle- and estrogen-treated cells is represented as a percentage of clones harboring C-to-T changes. Clones from three independent experiments were sequenced, for a total of >100 clones per treatment. (C) Each identified C-to-T transition is shown (blue dots), mapped to the region of the TFF1 gene (−520 to +80) amplified by 3D-PCR. (D) MCF7 cells transfected with A3B or control siRNA were treated with estrogen as described above. (E) 3D-PCR amplicons were cloned. Shown are the percentage of clones harboring C-to-T transitions for a total of >150 clones generated from three experiments. (F) 3D-PCR of genomic DNA from MCF7 cells transfected with UNG siRNA were treated with estrogen. (G) Hormone-depleted MCF7 and T47D cells transfected with siUNG were assessed for growth in the presence or absence of estrogen (^∗^p < 0.001; n = 4). (H) Real-time PCR using RNA prepared from MCF7 cells transfected with siUNG, following 12 hr treatment with estrogen (10 nM) (^∗^p < 0.001; n = 3). (I) HeLa cells cultured in hormone-free medium were transfected with an estrogen-responsive luciferase reporter gene, together with ER, A3B, and myc-UGI (n = 3). Immunoblotting is shown in the inset. (J) MCF7 cells transfected with myc-UGI were treated with estrogen for 12 hr, and RNA and protein were isolated. Real-time PCR and immunoblotting for ER target genes are shown. (K) Hormone-depleted MCF7 and T47D cells transfected with myc-UGI were grown in the presence or absence of estrogen for 5 days. Growth was measured using the sulforhodamine B (SRB) assay (^∗^p < 0.001; n = 4).

Mutational inactivation of the A3B catalytic domains inhibited ER stimulation in reporter gene assays (Figure 2A), implying that deamination of deoxycytidine to deoxyuridine (C to U) is required for the regulation of estrogen-responsive gene expression by A3B. We used differential DNA denaturation PCR (3D-PCR), which identifies C-to-T changes, based on detecting PCR amplicons at lower denaturation temperatures arising from an increase in A/T content (Burns et al., 2013a; Suspène et al., 2005), to determine if A3B causes C-to-U changes at ER binding regions. Estrogen treatment of MCF7 cells generated lower-temperature amplicons in the TFF1 promoter region (Figure 3A). Cloning and sequencing of the PCR products from three independent experiments identified C-to-T changes in a total of 12/109 (11%) clones from vehicle-treated cells, increasing to 43/114 (38%) following estrogen treatment (Figure 3B). Importantly, the overwhelming majority (37/43 [86%]) of these changes mapped to the A3B binding region (Figure 3C). Sequencing of 3D-PCR products following A3B knockdown identified C-to-T changes in a total of 58/155 (37%) clones in siControl-transfected, compared with 13/163 (8%) clones in siA3B-transfected, MCF7 cells (Figures 3D and 3E), demonstrating that A3B is necessary for the C-to-U transitions as the TFF1 ER/A3B binding region. Similar results were obtained for the PDZK1 ER/A3B binding region, if A3B was knocked down in T47D cells (Figures S4A–S4E).

Failure to repair cytidine deamination would result in the accumulation of deleterious mutations at gene enhancers. As dU is excised by uracil DNA glycosylase (UNG) (Stavnezer, 2011), we determined if UNG is required for the A3B-dependent cytidine deamintion at ER binding regions. UNG knockdown resulted in lower-temperature amplicons at ER/A3B binding regions (Figure 3F), suggesting that lack of UNG “fixes” the A3B directed C-to-U changes in DNA. The consequence of UNG knockdown was inhibition of ER target gene expression and MCF7 and T47D cell growth (Figures 3G, 3H, and S4F).

**Figure 4:**
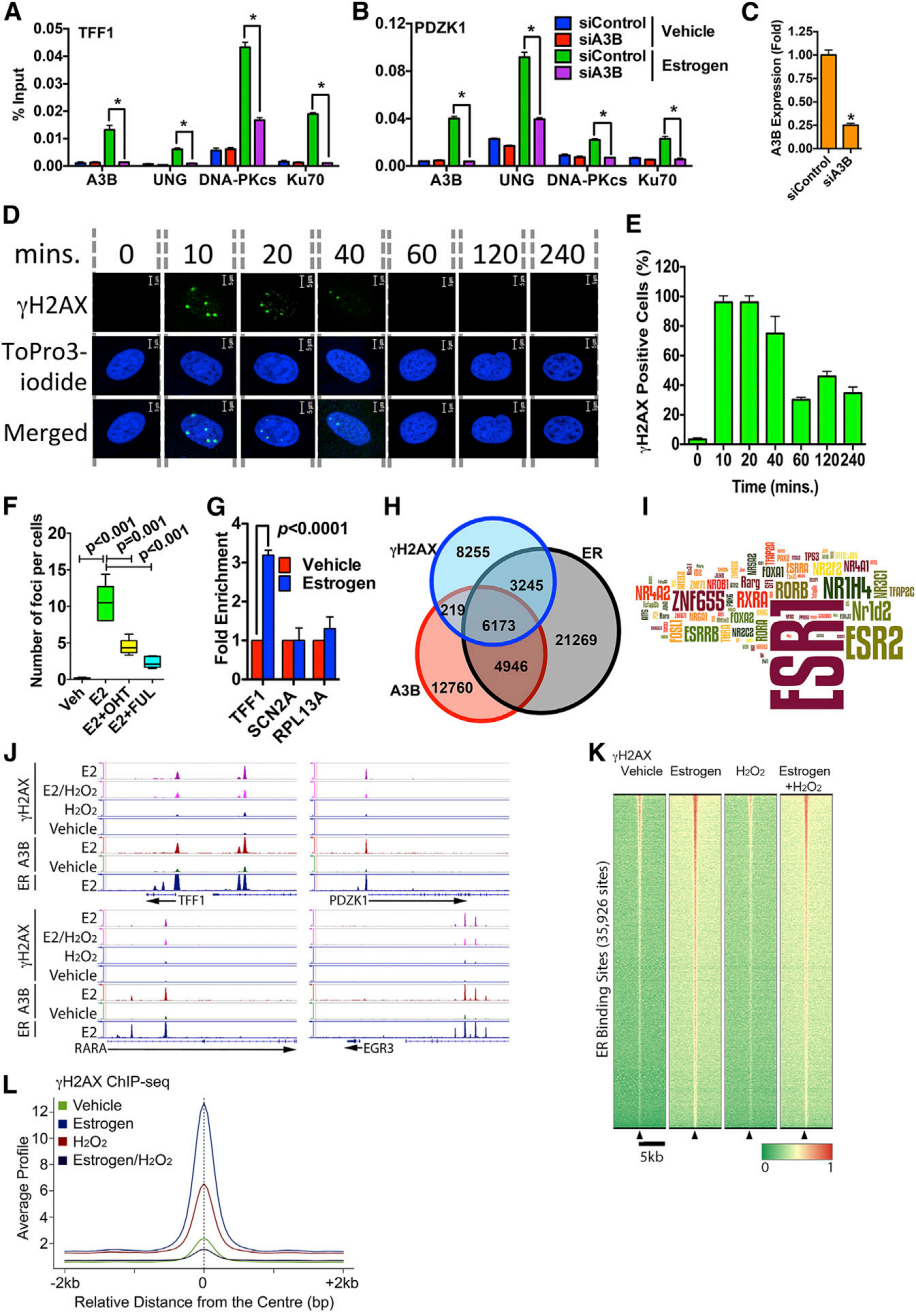
Estrogen Treatment of MCF7 Cells Induces DSBs at ER Binding Regions Hormone-depleted MCF7 cells were used in all experiments. (A and B) ChIP for A3B, UNG, DNA-PKcs, and Ku70 was performed following estrogen addition to MCF7 cells transfected with siA3B or siControl. Real-time PCR was performed on recovered DNA, using primers flanking the ER binding regions in TFF1 and PDZK1 genes (n = 3, ^∗^p < 0.001). (C) A3B mRNA levels by real-time PCR for samples used above. (D) Estrogen was added and cells were immunostained for γH2AX. Nuclei were visualized with the TOPRO DNA stain. (E) γH2AX in 100 cells from five replicates (total n = 500) was quantified using Cell Profiler 2.0. (F) OHT or FUL was added for 1 hr, followed by addition of estrogen (E2) for 10 min. Boxplots show the mean γH2AX foci number in 100 cells (n = 5). (G) γH2AX ChIP (MCF7) and real-time PCR for the TFF1 ER binding site or promoter regions of the SCN2A1 and RPL13A genes (n = 3). (H) Venn diagram of γH2AX, A3B, and ER binding events from ChIP-seq experiments for estrogen-treated cells. Individual peaks were identified using the same peak-calling algorithm (MACS1.4) using identical settings. (I) Analysis of the relative enrichment of transcription factor binding sites was used to generate a *Z* score, which is represented by the size of the motif in the word cloud. (J) Genome browser snapshots of γH2AX ChIP-seq in MCF7 cells treated with estrogen, H_2_O_2_, or vehicle. (K) Heatmap showing clustered binding signals for regions enriched in γH2AX for all treatment conditions. The windows represent ±5.0-kb regions from the center of the ER binding events. The color scale shows relative enrichment based on raw signal. (L) Average signal intensities of γH2AX ChIP-seq binding events centered on ER binding regions are shown for the different treatments. Signal intensity is a normalized count of individual, non-redundant ChIP fragments at single ER binding sites identified with the MACS1.4 peak-calling algorithm.

We used the bacteriophage PBS2 uracil DNA glycosylase inhibitor (UGI), which represses UNG activity in mammalian cells (Burns et al., 2013a), to confirm the involvement of UNG. In a reporter gene assay, ectopic expression of UGI prevented the stimulation of ER activity by A3B (Figure 3I). UGI transfection also repressed endogenous ER target gene expression and estrogen-stimulated growth of MCF7 and T47D cells (Figures 3J, 3K, and S4G). Finally, ChIP showed that UNG was recruited to ER binding regions in the TFF1 and PDZK1 genes, its recruitment being prevented by A3B knockdown (Figures 4A–4C). Thus, UNG is required for ER function and is recruited to ER binding sites in an A3B-dependent manner. Importantly, these results indicate that repair of A3B driven cytidine deamination involves the action of UNG.

### Estrogen Binding to ER Promotes DNA Strand Breaks at ER Binding Regions

The mechanism of immunoglobulin gene class switch recombination involves cytidine deamination by AID and subsequent dU excision by UNG results in the generation of DNA strand breaks that can be repaired by the NHEJ pathway (Kotnis et al., 2009; Nowarski et al., 2012). Moreover, high-level A3G expression in leukemia cells promotes DNA strand breaks (Kotnis et al., 2009; Nowarski et al., 2012). Finally, A3B overexpression promotes γH2AX (Burns et al., 2013a), which is activated at DNA strand breaks (DSB) and is thus a marker for DSB. We reasoned, therefore, that targeted A3B-mediated cytidine deamination and dU excision by UNG could promote DSB generation at ER/A3B enhancers. If so, then estrogen treatment of breast cancer cells should be sufficient to cause DSBs in breast cancer cells. Indeed, treatment of MCF7 cells with estrogen induced γH2AX within 10 min (Figures 4D and 4E). γH2AX induction required ER, since synthetic ER ligands also induced γH2AX (Figures S5A and S5B), while treatment with anti-estrogens 4-hydroxytamoxifen (OHT) or fulvestrant (FUL) prevented estrogen induction of γH2AX (Figures 4F and S5C), as did transfection with ER siRNA (Figure S5D). Estrogen similarly induced γH2AX in an ER-dependent manner in T47D cells (Figures S5E and S5F), but not in the ER- MDA-MB-231 cells (Figure S6A). However, estrogen stimulation of γH2AX was possible in MDA-MB-231 cells ectopically expressing ER.

Estrogen induced γH2AX required DNA-PK and ATM activities and the γH2AX foci co-localized with 53BP1 (Figures S5G and S5H), verifying that estrogen treatment promotes DSBs. We used ChIP for γH2AX to determine if the DSBs localize to ER and A3B binding regions. Estrogen treatment stimulated γH2AX at the ER/A3B binding region in the TFF1 gene, but no γH2AX enrichment was observed at the promoter of a gene that is not expressed in MCF7 cells (SCN2A) (Ju et al., 2006), nor was γH2AX enrichment observed at the non-ER target gene RPL13A, which is expressed in MCF7 cells (Figure 4G). We undertook γH2AX ChIP-seq to determine the global distribution of γH2AX following estrogen treatment (Figure S7A). Peak calling identified 17,892 γH2AX binding events in the estrogen-treated samples. Using the definition that a binding region must overlap by at least one base pair, 54% (9,637/17,892) of γH2AX regions co-localized with A3B and/or ER binding events, with two-thirds (64%, 6,173/9,637) of these regions co-localizing to A3B and ER co-incident binding events (Figure 4H). Motif enrichment analysis confirmed that the γH2AX regions are highly enriched for ER (ESR1) binding motifs (Figure 4I). Correlation coefficient values for the raw sequencing data confirmed the co-localization of γH2AX regions with A3B (*r*^*2*^ = 0.69) and ER (*r*^*2*^ = 0.70) binding events (Figure S7B). Genomic loci exemplifying γH2AX at A3B and ER binding regions are shown in Figure 4J.

Aligning the γH2AX peaks showed very little enrichment for γH2AX in vehicle-treated cells at ER binding regions (Figures 4K and 4L). H_2_O_2_ treatment, which induced γH2AX, also did not result in much enrichment of γH2AX at ER binding regions. Indeed, co-treatment with H_2_O_2_ and estrogen reduced γH2AX at ER regions, compared with estrogen alone. Indeed, the majority of γH2AX regions induced by estrogen were enriched for ER and A3B binding (Figure S7C). There was some enrichment for ER and A3B sites in γH2AX regions that were common to all treatments. However, there was very little overlap with ER or A3B sites for γH2AX regions present in vehicle treated, or following H_2_O_2_ treatment, indicating that estrogen/ER induced γH2AX occurs at sites that are quite distinct from those caused by DNA-damaging agents.

As described above, the greater part of ER and A3B binding occurs at distal regions. Active regulatory regions such as enhancers and promoters carry specific epigenetic modifications including H3K27ac (enhancers and promoters), H3K4me1 (enhancer specific), and H3K4me3 (promoter specific) (ENCODE Project Consortium, 2012). Interestingly, A3B was found at 93% of active enhancers and 7% of active promoters (Figure S7D). This was confirmed by strong enrichment for BRD4 and p300, two ubiquitous co-activators found at active regulatory elements (Hnisz et al., 2013). In addition, using recently published GRO-seq data (Hah et al., 2013), we could identify bi-directional transcription at a subset of distally bound A3B sites, indicating the possibility of eRNA synthesis at these elements.

### APOBEC3B Action at ER Target Genes Causes Transient DNA Strand Breaks That Are Repaired by NHEJ

**Figure 5:**
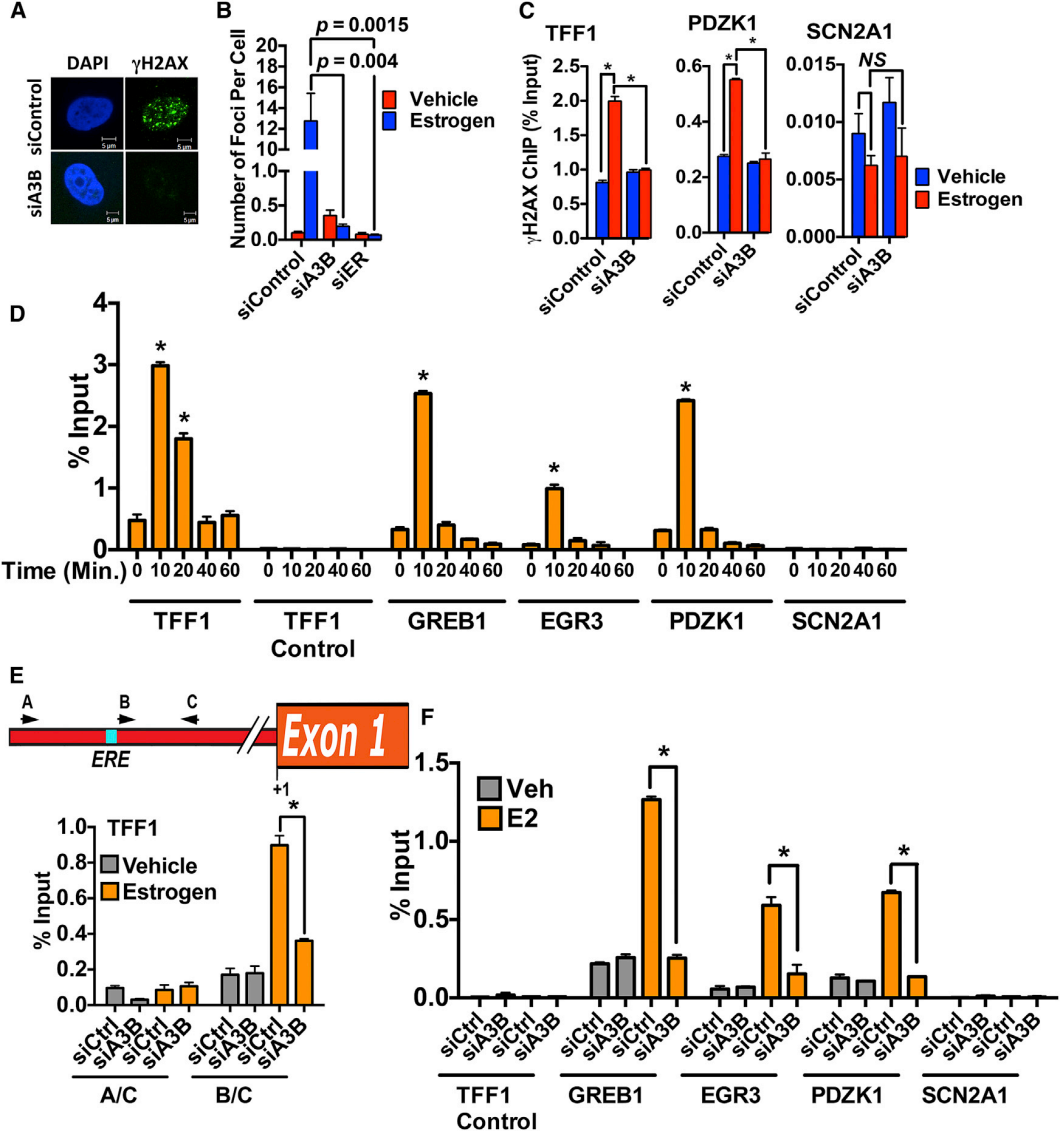
Estrogen-Induced DNA Strand Breaks Are Dependent on A3B (A) Hormone-depleted MCF7 cells were used for all experiments. Cells transfected with siA3B or siControl were treated with estrogen. Shown are representative images for γH2AX staining. (B) The average γH2AX foci number per cell in 100 cells from five replicates ± SEM. (C) ChIP assay with MCF7 cells transfected with A3B or control siRNA. (D) Estrogen was added and cells end labeled by incubation with biotin-16-dUTP in the presence of terminal deoxynucleotide transferase (TdT). ChIP was performed with a biotin antibody. (E and F) Estrogen was added following siRNA transfections for 10 min. Biotin end labeling was performed as in (D). (C–F) n = 3; ^∗^p < 0.001; NS, not significant.

Our results demonstrate that A3B induces C-to-U transitions and promotes UNG, DNA-PK, and Ku70 recruitment to ER binding regions (Figures 4A and 4B). We have also shown that estrogen/ER rapidly induces γH2AX globally at ER and A3B binding regions. These findings imply that A3B action is required for DSB generation at ER binding regions. Indeed, A3B knockdown blocked estrogen-induced γH2AX in MCF7 cells (Figures 5A, 5B, and S5D) and prevented γH2AX at ER binding sites in the TFF1 and PDZK1 genes (Figure 5C). Similarly, UNG was required, as its knockdown also inhibited estrogen induction of γH2AX (Figure S5I). Moreover, estrogen induction of γH2AX in MDA-MB-231 cells expressing ER required A3B (Figures S6B and S6C). A3B was also necessary for optimal expression of ER-regulated genes in the ER-expressing MDA-MB-231 lines (Figure S6D).

Next, we used biotin-16-deoxyuridine triphosphate (dUTP) labeling of DSBs with terminal deoxynucleotide transferase (TdT) (Ju et al., 2006) followed by biotin ChIP and real-time PCR to directly determine if DSBs are formed at ER/A3B binding regions. Real-time PCR using primers located 3′ to the region in the TFF1 gene to which the great majority of C-to-U transitions mapped showed 6-fold enrichment over the vehicle control within 10 min of estrogen addition (Figure 5D). DSBs in this region were reduced to basal levels by 60 min, in general agreement with the reduction in γH2AX over this time frame. A similar rapid but transient induction of DSBs was observed for other ER target genes, but there were no detectable DSBs at the non-expressed SCN2A1 gene promoter, or at a region 2 kb 5′ to the ER binding region in TFF1 (TFF1 control). PCR using primers A/C, which amplify across the region containing the C-to-U transitions failed to show estrogen stimulation of DSBs, indicating that estrogen induces DSBs within the region of the TFF1 gene that is characterized by A3B-mediated C-to-U transitions (Figure 5E). Estrogen induction of DSBs was prevented if the cells were transfected with A3B siRNAs (Figures 5E and 5F).

### A3B Is Required for Histone Modification and Recruitment of Chromatin Remodeling Factors at ER Binding Regions

**Figure 6:**
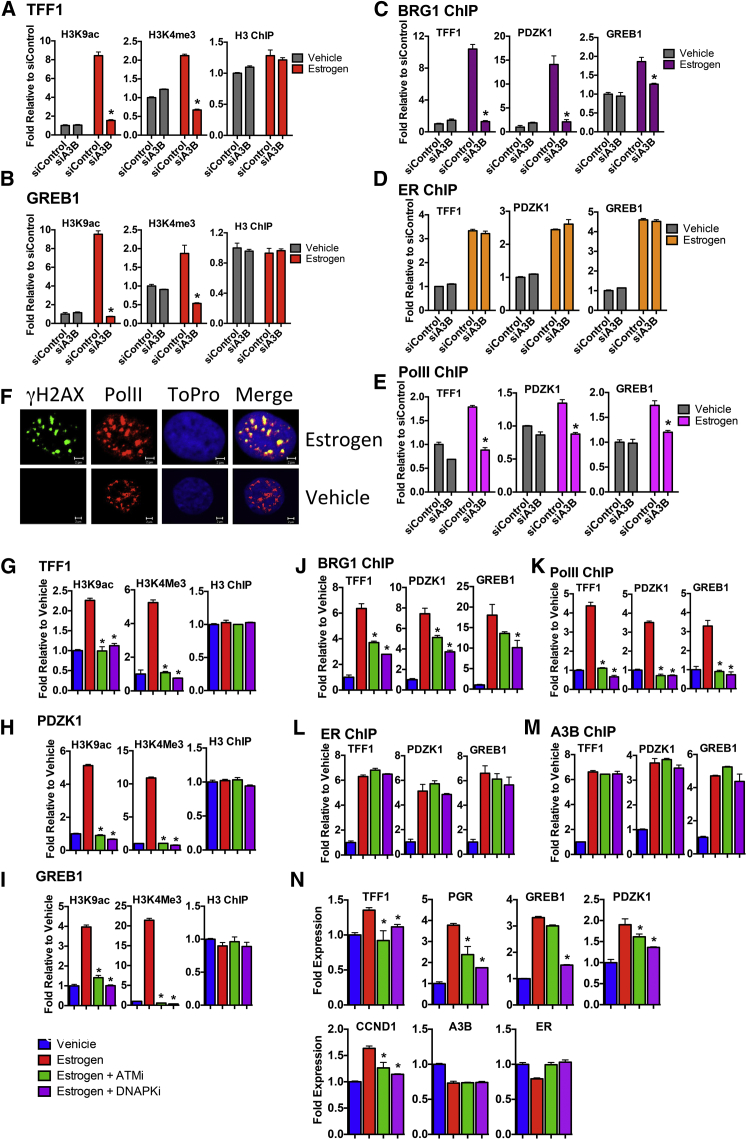
A3B Is Required for Chromatin Remodeling and Activating Histone Modifications at ER Enhancers All experiments were undertaken with hormone-depleted MCF7 cells. (A and B) ChIP for histone H3 and H3 modifications associated with gene activation, was performed following estrogen addition to cells transfected with siA3B or control siRNA (n = 3, ^∗^p < 0.001). (C–E) ChIP for BRG1, ER, and PolII followed by real-time for A3B/ER binding sites in TFF1, PDZK1, and GREB1. (F) Cells were treated with estrogen for 10 min and immunostained for γH2AX (green) and PolII (red). (G–M) 5 μM NU7441 (DNA-PKcs inhibitor) or KU55933 (ATM inhibitor) was added for 1 hr followed by estrogen addition. ChIP was performed as above (n = 3, ^∗^p < 0.001). (N) Cells were treated with NU7441 or KU55933 for 1 hr, at which point estrogen was added. Real-time RT-PCR was performed with RNA prepared after 4 hr (n = 3; ^∗^p < 0.001).

Transcription factors regulate gene expression by promoting the ordered recruitment of diverse complexes that modify and remodel chromatin, leading to transcription initiation. Recent studies show that DNA repair factors regulate gene expression by aiding chromatin remodeling (Fong et al., 2013). A3B knockdown prevented estrogen stimulation of histone modifications associated with transcription at ER target genes (Figures 6A and 6B). Importantly, H2AX phosphorylation at serine-139 (γH2AX) promotes chromatin recruitment of BRG1, the catalytic subunit of the SWI/SNF ATPase-dependent chromatin remodeling complex (Lee et al., 2010). In agreement with a model in which A3B-mediated cytidine deamination leads to H2AX activation, A3B knockdown prevented BRG1 recruitment (Figures 6C and 6D). A3B knockdown also inhibited PolII recruitment to the TFF1 and GREB1 genes. Interestingly, the estrogen-stimulated γH2AX co-localized with activated (phosphorylated) PolII, further evidence that the estrogen/ER and A3B regulated DSB formation occurs of transcriptionally active chromatin. In addition to regulating histone modification and recruitment of chromatin remodelers, A3B was required for PolII recruitment to ER/A3B binding regions (Figures 6E and 6F). The importance of H2AX activation was underscored by the fact that treatment with the DNA-PKcs inhibitor NU7441 or the ATM inhibitor KU55933 inhibited histone modification, as well as BRG1 and PolII recruitment at ER target genes (Figures 6G–6M). Note that these treatments did not affect A3B and ER recruitment.

## Discussion

The molecular mechanisms by which ER drives breast cancer has identified transcription factors that direct ER to active enhancers (Magnani et al., 2011) and revealed that ER controls the coordinated recruitment of chromatin remodeling and modification proteins and the transcription machinery that together facilitate the program of estrogen-responsive gene expression (Métivier et al., 2006). There is also gathering evidence that many protein complexes that sense and repair DNA damage are important for regulation of gene expression by ER and other transcription factors, their recruitment aiding chromatin modification/remodeling to promote gene activation (Fong et al., 2013). For example, the BER protein thymine DNA glycosylase acts as a co-activator for ER and promotes recruitment of the p160 co-activators and the CBP/p300 histone acetyltransferase (Lucey et al., 2005; Tini et al., 2002). One reason advanced for the function of DNA repair proteins in gene regulation is that they may aid in relieving torsional stress generated by transcription-induced DNA supercoiling (Ma and Wang, 2014). Movement of RNA polymerase (RNAP) along the DNA template during transcription generates over-winding (positive DNA supercoiling) in front and negative DNA supercoiling behind it. Failure to resolve DNA supercoiling will ultimately affect transcription. Additionally, generation of DNA supercoiling at one promoter can affect transcription from a distal promoter, so-called topological promoter coupling (Ma and Wang, 2014). Given the recent identification of active transcription at enhancer regions, which generates eRNAs, RNAP procession is also likely to create torsional stress at enhancer regions, which might contribute to inhibition of transcription from coupled gene promoters. Furthermore, regulation of transcription by enhancer regions entails communication between distal enhancers and regulated promoters through chromatin looping, a process that is also influenced by DNA supercoiling (Kulaeva et al., 2012). Thus, DNA topoisomerases, which relax negative and positive DNA supercoils, are important in transcription regulation. Indeed, ER- and androgen receptor (AR)-induced transcription in breast and prostate cancer involves transient DSBs generated by TOP1 and TOP2 (Ju et al., 2006; Puc et al., 2015). Activation of the LSD1 histone demethylase by ER binding promoted 8-oxoguanine modification of DNA and recruitment of 8-oxoguanine-DNA glycosylase and BER to stimulate chromatin changes that facilitate interaction between ER-regulated gene enhancers and promoters (Perillo et al., 2008). Thus, DSB formation and their resolution is an important component of the regulation of gene expression by of ER.

**Figure 7:**
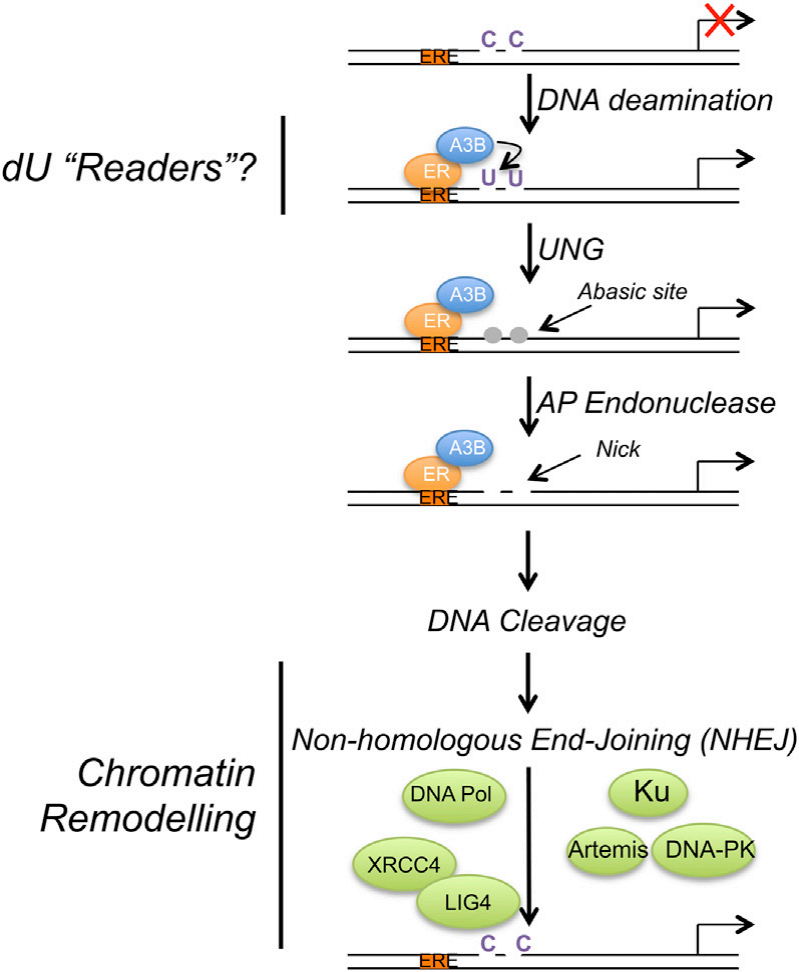
Model for A3B-Mediated Activation of ER Enhancers toward Regulation of ER Target Gene Expression Estrogen binding to ER promotes its recruitment to ER enhancers. A3B, recruited to these regions through interaction with ER, provides enzymatic conversion of C to U. Generation of U:G mismatches promotes DNA nicks through the action of UNG and AP endonuclease, resulting in DNA cleavage and repair by the non-homologous end-joining DNA repair pathway. Induction of transient C-to-U changes stimulates chromatin modification and remodeling and PolII recruitment to facilitate expression of ER target genes.

Here, we report an alternative mechanism for transcription of ER-regulated genes in which DSBs are generated in a process initiated by estrogen-ER-dependent recruitment of A3B. The importance of A3B in ER action is implied by our observation of the exceptionally high genome-wide co-localization of ER and A3B binding regions. Moreover, we show that estrogen treatment causes rapid induction of DSBs, as demonstrated by γH2AX activation. Importantly, the majority of estrogen-induced γH2AX occurs at ER and A3B binding regions, and we have shown that γH2AX induction is dependent on A3B. Our work demonstrates that A3B directs cytidine deamination at ER binding regions to facilitate DSBs through activation of BER and subsequent repair of these lesions by the NHEJ pathway. The critical role of A3B action in the regulation of gene expression by ER is established by its requirement for breast cancer cell growth in vitro and in vivo. Based on these findings, we propose that the A3B-mediated generation of C-to-U changes and activation of DNA repair pathways facilitates chromatin remodeling and enhancer/promoter interaction. In support of this, A3B is required for SWI/SNF recruitment, activating histone modifications and PolII recruitment to ER binding regions (a schematic model is shown in Figure 7).

Interestingly, DNA damage by irradiation or with the use of models in which DNA DSBs are locally induced with restriction enzymes such as I-SceI has demonstrated that ATM promotes dynamic chromatin condensation and transcriptional silencing at DSBs (Khurana et al., 2014; Shanbhag et al., 2010). DNA-PKcs can repress transcription (Pankotai et al., 2012), but its recruitment to transcription-factor-promoted DSBs stimulates transcription (Ju et al., 2006). As proposed by Tjian and colleagues (Fong et al., 2013), transient DSB generation by transcription factors may limit γH2AX accumulation and spreading to check retention of DNA repair factors and thus control the extent of the DDR response. In agreement with this model, DNA DSB-induced H2AX phosphorylation is spread over large domains around the DSB (Iacovoni et al., 2010). This contrasts with the restricted γH2AX distribution induced by A3B recruitment to ER binding regions observed here. Thus, the mode of ATM and DNA-PKcs recruitment appears to determine the effect of DSBs on transcription.

Proteins that preferentially bind to DNA sequences containing methylcytosine (mC) to regulate chromatin and control gene expression are well described. Importantly, proteins that bind to other CpG modifications, including 5-hydroxymethlycytosine (hmC), 5-formylcytosine (fC), and 5-carboxylcytosine (caC) are now being identified and include not only DNA repair factors but also chromatin regulators and transcription factors (Iurlaro et al., 2013; Spruijt et al., 2013). Our findings allow that there may be transcription regulatory proteins that interact with DNA containing dU and that are therefore recruited upon A3B-mediated cytidine deamination at gene enhancers, an intriguing possibility that may deserve further investigation.

Recent studies show that cytidine deamination is an important feature of the mutational landscape in breast (Alexandrov et al., 2013; Burns et al., 2013a; Nik-Zainal et al., 2012), ovarian (Leonard et al., 2013), lung (de Bruin et al., 2014), and other cancers (Burns et al., 2013b; Roberts et al., 2013), with high-level expression of A3B, and experimental studies indicate that A3B may be a key driver of such mutational signatures (Burns et al., 2013a; Taylor et al., 2013). It is tempting to speculate that the cytidine-deaminase-associated mutational landscapes in breast cancer might be enriched at ER/A3B binding regions. In support of this possibility, AR was shown to promote DNA DSBs to aid intra- and inter-chromosomal translocations in prostate cancer cells, one mechanism for which involved the hormone-dependent recruitment of AID (Lin et al., 2009). Determination of the global ER binding profiles by ChIP-seq analysis of breast tumors has shown that there is a high level of plasticity in ER binding in breast cancer (Ross-Innes et al., 2012), such that investigation of any association between A3B/ER binding regions and somatic mutations may entail whole-genome sequencing, coupled with ER and A3B ChIP-seq in the same tumor. Interestingly, however, gene regulatory regions can be characterized by low levels of somatic mutations in cancer in the absence of additional defects in DNA repair (Polak et al., 2014). Our results suggest that activation of DNA repair pathways may protect enhancer regions from A3B-dependent mutagenesis. It would be important therefore to investigate the involvement of defects in BER and/or NHEJ in cancer mutational landscapes that have been ascribed to A3B.

In summary, our results identify an important role for A3B as a regulator of ER-mediated gene expression in breast cancer, with potential as a therapeutic target in ER+ breast cancer. To advance this possibility, it will be important to extend our findings to studies that identify global A3B regulated genes in breast cancer cell lines, as well as in tumor samples. Moreover, as A3B is widely expressed in other cancers, it is likely that A3B inhibition represents an important therapeutic approach to inhibit regulated transcription in other cancer types.

## Experimental Procedures

### Cell Lines, Plasmids, Antibodies, and Real-Time RT-PCR Assays

Cell lines were obtained from and cultured in media recommended by ATCC. MCF7, T47D, SkBr3, COS-1 and HeLa cells were grown in DMEM containing 10% fetal calf serum (FCS). MDA-MB-231 cells stably expressing ER have been described (Bhat-Nakshatri et al., 2004). Hormone depletion was achieved by culturing cells for 72 hr in DMEM lacking phenol red and containing 5% dextran-coated charcoal-stripped FCS. Plasmids, antibodies, and real-time PCR primers are detailed in the Supplemental Experimental Procedures.

### Reporter Gene Assays

Hormone-depleted COS-1 cells were transfected with ERE3-TATA-luc, pRL-TK, together with ER and hemagglutinin (HA)-tagged A3B. Estrogen (10 nM) or an equal volume of ethanol (vehicle) was added 5 hr following transfection. Transfection methodology and luciferase measurements were performed as described previously (Lai et al., 2013). Reporter gene assays in HeLa cells following transfection with ER, A3B, and myc-UGI-NLS were undertaken as above.

### siRNA

Cells were transfected with siRNA using Lipofectamine RNAiMax (Invitrogen). For collecting RNA and protein, 10 nM estrogen was added after 48 hr; RNA and protein lysates were prepared after a further 12 hr. Cell growth was determined using the sulforhodamine B (SRB) assay, as described previously (Lai et al., 2013). Details of siRNAs are provided in the Supplemental Experimental Procedures.

### MCF7 Human Tumor Xenografts

10 μM siRNA prepared with the atelogene in vivo siRNA transfection kit (Koken, Japan) was injected weekly directly into tumors. Tumor volumes were determined twice weekly. At the end of the experiment, protein lysates were prepared from half of each tumor by homogenization in RIPA buffer. RNA was prepared from the remaining halves of each tumor using the RNAeasy kit (QIAGEN). The study was undertaken under the auspices of a UK Home Office project license, using approved procedures.

### Gene Expression

Total RNA was prepared and real-time RT-PCR was performed as described previously (Ngan et al., 2009), using TaqMan gene expression assays from ABI.

### Immunoprecipitations

Immunoprecipitations were performed as described previously (Lopez-Garcia et al., 2006).

### Immunofluorescence

Cells were cultured on glass coverslips in phenol red-free DMEM containing 5% double charcoal-stripped FCS for 3 days before the addition of ligands. Cells were fixed and incubated with antibodies, as described in the Supplemental Experimental Procedures. Images were acquired using a Zeiss LSM510 confocal microscope. Images were analyzed using Fuji Image J (NIH) and CellProfiler (Broad Institute) for quantification of staining.

### ChIP

ChIP was performed as described previously (Lai et al., 2013), using 10 μg of antibody and 100 μl of Protein A Dynalbeads (10002D; Invitrogen). Control ChIP was performed by the addition of mouse immunoglobulins (IgG).

### ChIP and Solexa Sequencing

ChIP DNA was amplified as described (Schmidt et al., 2009). Sequences were generated by the Illumina Hiseq 2000 genome analyzer (using 50 bp reads) and aligned to the Human Reference Genome (assembly hg19, February 2009) using Bowtie 1.0. The model-based analysis for ChIP-seq (MACS) peak caller version 1.4 (Zhang et al., 2008) was used to identify enriched regions of the genome by comparison to an input sample. MACS was used in the default setting with a p value threshold of 10^−5^. To call stimuli specific peaks, we used bedtool to subtract or concatenate BED files generated by MACS. The number of reads obtained, percentage of reads aligned, peaks called, and additional analysis methods are detailed in the Supplemental Experimental Procedures.

### Biotin Labeling of DSB

Biotin labeling was performed as described previously (Ju et al., 2006) and detailed in the Supplemental Experimental Procedures.

### 3D-PCR, Cloning, and Sequencing

3D-PCR was carried out as described elsewhere (Suspène et al., 2005). Genomic DNA was prepared using the Invitrogen genomic extraction kit. Products from the second round PCR were purified using the QIAGEN PCR purification kit, cloned into the TOPO-TA cloning vector (Invitrogen), and sequenced with the T7 sequencing primer.

## Accession Numbers

The accession numbers for the ChIP-seq data reported in this paper are GEO: GSE56979 (A3B ChIP-seq) and GEO: GSE57426 (γH2AX ChIP-seq).

## Supplemental Information

Document S1. Supplemental Experimental Procedures and Figures S1–S7

Table S1. DNA Motif Enrichment Analysis, related to Figures 2 and 4

Document S2. Article plus Supplemental Information
