# Supplementary material for: APOBEC3B-Mediated Cytidine Deamination Is Required for Estrogen Receptor Action in Breast Cancer
Source: Cell Rep. 2015 Sep 24;13(1):108–21. doi: 10.1016/j.celrep.2015.08.066 (PMC4597099; doi:10.1016/j.celrep.2015.08.066)
Supplement: Document S1. Supplemental Experimental Procedures and Figures S1–S7 [file mmc1.pdf]

Cell Reports

Supplemental Information

APOBEC3B-Mediated Cytidine Deamination Is Required for Estrogen Receptor Action in Breast Cancer

Manikandan Periyasamy, Hetal Patel, Chun-Fui Lai, Van T.M. Nguyen, Ekaterina Nevedomskaya, Alison Harrod, Roslin Russell, Judit Remenyi, Anna Maria Ochocka, Ross S. Thomas, Frances Fuller-Pace, Balázs Győrffy, Carlos Caldas, Naveenan Navaratnam, Jason S. Carroll, Wilbert Zwart, R. Charles Coombes, Luca Magnani, Laki Buluwela, and Simak Ali

Supplemental Experimental Procedures

Cell lines. All cell lines were tested on a three-monthly basis to ensure they were free from mycoplasma infection and were genotyped for authenticity using the STR profiling cell line authentication service from LGC Standards (UK).

Plasmids. The expression plasmid pSG5, pSG5 encoding ER and the estrogen-responsive firefly luciferase reporter gene (ERE3-TATA-luc) have been described previously (Lopez-Garcia et al., 2006). The Renilla luciferase reporter gene (pRL-TK) was purchased from Promega, UK. HA-tagged human A3B was kindly provided by Prof B. Cullen and has previously been described (Bogerd et al., 2007). Mutagenesis of HA-A3B was performed by site-directed mutagenesis (Stratagene, UK). Mutagenesis primers had the sequences 5'-GTATCCTGGACCCCCgcCCCGGACgcTGTGGCGAAGCTGGCC-3' (A3B-C97A/C100A) and 5'-CATCTCCTGGAGCCCCgcCTTCTCCTGGGGCgccGCCGGGGAAGTG-3' (A3B-C284A/C289A), where the bases shown in lower case are altered from the wild-type sequence, for amino acid substitutions. Drs B Cullen, H. Weingard, M-A Langlois and M Neuberger kindly provided the other APOBEC expression plasmids. Bacteriophage PBS2 UGI, cloned in frame with a nuclear localisation signal (Reddy et al., 1998), kindly provided by Prof J Cohen, was cloned into pCMV6-Entry mammalian expression plasmid, to generate myc/FLAG-tagged UGI-NLS.

siRNA studies. Cells were cultured in DMEM lacking phenol red and containing 5% DSS for three days prior to transfection with siRNA using the Lipofectamine RNAiMax transfection reagent (Invitrogen), according to manufacturer's protocols. Transfections were performed in 6-well plates for RNA and protein preparation, or in individual wells of 96-well plates for growth studies. For collecting RNA and protein, 10 nM estrogen was added after 48 hours. Cells were harvested for RNA and protein after a further 12 hours. For growth assays, estrogen was added 24 hours after transfection. Cell growth

was determined using the Sulphorhodamine B (SRB) growth assay, as described (Thiruchelvam et al., 2011). siRNA from Qiagen was used for ER (SI03114979, 5'-UCCGAGUAUGAUCCUACCAGA-3') and non-targeting control (siControl; AM4635, Ambion). A3B siRNAs had the sequences, 5'-CCUGAUGGAUCCAGACACA-3' (siA3B#1) and 5'-GGUGUAUUUCAAGCCUCAGTT-3' (siA3B#2). siA3B (3'UTR) had the sequence 5'-AAGUGAUUAAUUGGCUCCAUA-3' (Qiagen cat. No: S104180708). UNG knockdown was carried out using Dharmacon pool cat. No. L011795-00.

Immunoblotting. Cell lysates were prepared and Western blotting was performed as described previously (Lai et al., 2013). Antibodies for ER (HC20; sc-543), TFF1 (sc-28925), BCL2 (sc-492), PGR (sc-538), lamin A/C (sc-7292) and HA tag (sc-7392) were purchased from Santa Cruz. Antibodies for cathepsin D (CTD; ab6313), hsp90 (ab13492), and ß-actin (ab6276) were purchased from Abcam, UK. ER (NCL-ER-6F11) and FLAG (F1804) antibodies were from Novocastra and Sigma-Aldrich, respectively. Commercially available antibodies for AIB1 (611105, BD Biosciences), PDZK1 (10507-2-A, Proteintech) and myc (05-724, Millipore) were used. Immunoblotting for A3B was carried out using a rabbit polyclonal antiserum prepared by immunizing with recombinant protein.

Real-time RT-PCR. Total RNA was prepared and real-time RT-PCR was performed as described previously (Lai et al., 2013), using gene expression assays from Applied Biosystems, tabulated below.

Gene	***Assay Number***
AICDA	Hs00757808
APOBEC1	Hs00242340
APOBEC2	Hs00199012
APOBEC4	Hs00378929
APOBEC3A	Hs00377444
APOBEC3B	Hs00358981
APOBEC3C	Hs0028074

APOBEC3D	Hs00537163
APOBEC3F	Hs01665324
APOBEC3G	Hs00222415
APOBEC3H	Hs00419665
BCL2	Hs00411268
Cathepsin D (CTD)	Hs00157201
CCND1	Hs00233498
ESR1	Hs00174860
GAPDH	Hs99999905
GREB1	Hs00536409
PDZK1	Hs00275727
PGR	Hs00172183
TFF1	Hs00170216
UNG	Hs00422172

Co-immunoprecipitations. Immunoprecipitations were carried out as described previously (Lopez-Garcia et al., 2006). Briefly, cells were lysed in RIPA buffer (150 mM NaCl, 1% NP-40, 0.5% deoxycholic acid, 0.1% SDS and 50 mM Tris-HCl (pH 7.5) containing protease inhibitors. Lysates were pre-cleared by incubating with agarose beads (Santa Cruz) at 4°C for 2 hours. Antibodies were incubated with agarose beads for 2 hours at 4°C. Following washing three times with ice-cold PBS, the beads were resuspended in PBS and transferred to the pre-cleared lysates for overnight incubation. Following incubation with protein lysates, the beads were washed in ice-cold PBS and re-suspended in 2X sample buffer (0.125 M Tris-HCl at pH 6.8, 4% SDS, 20% Glycerol, 10% β-mercaptoethanol and 0.004% bromophenol blue), heated at 95°C for 10 minutes and immunoblotted.

Immunofluorescence. MCF7 cells were cultured on glass coverslips in phenol red-free DMEM containing 5% dextran-coated charcoal-stripped FCS, for 3 days before the addition of 10nM E2, 100 nM fulvestrant or 100 nM 4-hydroxytamoxifen, as appropriate. Cells were fixed with 4% paraformaldehyde for 10 minutes and permeabilsed with 0.2% Triton X-100 for a further 10

minutes. Non-specific staining was blocked with blocking solution (10% fetal calf serum/3% bovine serum albumin in PBS) for 1 hour, followed by incubation with γH2AX (05-636, Millipore), 53BP1 (ab36823, Abcam), PolII phospho-serine 2 (Ab5095, Abcam) or ER (HC-20, Santa Cruz) antibodies for 1 hour. Cells were washed three times in blocking buffer and the slides were incubated with Alexafluor 488 and 555 labeled antibodies (Invitrogen) for 1 hour. Cells were washed three times in blocking buffer and then DAPI and ToPro (Invitrogen) nuclear dyes were used to visualise nuclei. Cells were mounted with VectaShield (Vector labs) for confocal analysis. Images were acquired using the Carl Zeiss confocal microscope using the LSM 510 image browser. Images were analysed using Fuji Image J (NIH, USA) and CellProfiler (Broad Institute, USA) to quantitate number of foci per cell.

MCF-7 Human tumour xenografts. Female, 7-week-old, nu/nu-BALB/c athymic nude mice were purchased from Harlan Olac Ltd, UK. The study was undertaken under the auspices of a UK Home Office project license, using approved procedures. 0.72-mg 17β-estradiol 60-day release pellets (Innovative Research of America) were implanted subcutaneously prior to subcutaneous injection of MCF-7 cells (5×10^6) in a volume of 100µl into the flank of the animals. When tumours had reached 50-100 mm^3, they were randomly assigned into three groups, the vehicle, siControl and the siA3B treatment groups. Tumour-bearing mice were treated weekly by injection of 10 µM siRNA prepared with the atelogene *in vivo* siRNA transfection kit (Koken, Japan) directly into tumours. Tumour diameters were measured twice weekly with digital calipers and tumour volumes were calculated using the formula, length (L) X $width^2$ (W)/2. At the end of the experiment, tumours were excised and snap frozen. Tumours were divided in two and protein lysates were prepared by adding 400 µl RIPA buffer, including protease and phosphatase inhibitors and homogenizing in tubes containing 2.8 mm ceramic beads (Peqlab; cat no: 91-PCS-CK28) using a Precellys-24 homogeniser. Lysates were cleared by centrifugation for 5 min. at 13,000 rpm. RNA was prepared from the remaining halves of each tumour using the RNAeasy kit (Qiagen). Real-time RT-PCR and immunoblotting was performed as described above.

Chromatin immunoprecipitation (ChIP). Cells were cultured in estrogen-free medium for 3 days before addition of 100nM estrogen. ChIP assays were performed as described previously (Lai et al., 2013), using antibodies listed below, or the in-house rabbit A3B polyclonal antiserum. Control ChIP was performed by the addition of mouse immunoglobulins (IgG). For each ChIP, 10μg of antibody and 100μl of Protein A Dynalbeads (10002D; Invitrogen) were used.

Antibody	Catalogue No.	Supplier
ER	sc-543	Santa Cruz
DNA-PKcs	sc-9051	Santa Cruz
Ku70	sc-5309	Santa Cruz
BRG1	sc-10768	Santa Cruz
BRM	sc-6450	Santa Cruz
Histone H3	**Ab1791**	Abcam
H3K9ac	**Ab4441**	Abcam
H3K4me3	**Ab8580**	Abcam
UNG	ab23926	Abcam
γH2AX	05-636	Millipore
RNA PolII	05-623	Millipore

Real-time PCR (qPCR) was performed on the enriched DNA using primers listed below.

Gene		***Sequence***
TFF1 promoter proximal ER binding region (-340/-54 bp)	Forward	TATGAATCACTTCTGCAGTGAG
	Reverse	GAGCGTTAGATAACATTTGCC
TFF1 control (-685/-520 bp)	Forward	GTGATTCTCCTGACTTAACC
	Reverse	TGGCGCAGTGGCTCACGCTG
GREB1	Forward	GGGTGAAATGAAGTGGCATGTG
	Reverse	GAACAAAACAGAGCAAGGCCAAA
SCN2A1	Forward	AGAGTGGGCTTTGGTTTCCT
	Reverse	CCATCAGAAGCCAAAATGCT

RPL13A	Forward	CACGTTCTCTTGGACAGCAA
	Reverse	TTAAGGCTCCGCTGAGAGAG

Chromatin Immunoprecipitations and Solexa sequencing (ChIP-seq). ChIP DNA was amplified as described (Schmidt et al., 2009). Sequences were generated by the Illumina Hiseq 2000 genome analyser (using 50 bp reads), and aligned to the Human Reference Genome (assembly hg19, February 2009) using Bowtie 1.0. Enriched regions of the genome were identified by comparing the ChIP samples to an input sample using the MACS peak caller (Zhang et al., 2008), version 1.4. The number of reads obtained, the percentage of reads aligned and number of peaks called, are detailed in figs. S3D and S7A.

Motif analysis, heatmaps and genomic distributions of binding events. ChIP-seq data snapshots were generated using the Integrative Genome Viewer IGV 2.3 (www.broadinstitute.org/igv/). Motif analyses were performed using Cistrome (cistrome.org), applying the SeqPos motif tool (He et al., 2010). The genomic distributions of binding sites were analysed using the *cis*-regulatory element annotation system (CEAS) (Ji et al., 2006). The genes closest to the binding site on both strands were analysed. If the binding region is within a gene, CEAS software indicates whether it is in a 5′UTR, a 3′UTR, a coding exon, or an intron. Promoter is defined as a region 0-3 kb upstream from RefSeq 5′ start. If a binding site is >3 kb away from the RefSeq transcription start site, it is considered distal intergenic. For integration with gene expression data, binding events were considered proximal when identified in a gene body or within 20kb upstream of the transcription start site. Heatmaps were plotted using the CHASE software package (http://chase.cs.univie.ac.at/overview).

Biotin labeling of DNA strand breaks. Biotin labelling of DNA strand breaks was performed as described (Ju et al., 2006). Estrogen was added to MCF-7 cells cultured for 72 hours in estrogen-free medium. Cells were fixed with Streck Tissue Fixative (STF, Streck Laboratories) for 10 minutes at 37°C, scraped from plates, washed twice with ice-cold PBS and centrifuged at 2,000

rpm for 5 minutes. Cell pellets were re-suspended in Buffer A (0.25% Triton X-100, 10mM EDTA, 10mM HEPES [pH6.5]) and placed on a rotating wheel for 10 minutes. Cells were centrifuged at 2,000 rpm for 5 minutes and pellets were re-suspended in Buffer B (200mM NaCl, 1mM EDTA, 10mM HEPES pH6.5), and permeabilized with Buffer C (100mM Tris-HCl pH7.4, 50mM EDTA, 1% Triton X-100) for one hour at 4°C. The nuclear pellets thus prepared were sequentially washed with ice-cold PBS, deionized water and 1x terminal deoxynucleotide transferase (TdT; Promega) reaction Buffer. To label DNA breaks the nuclei were incubated with biotin-16-dUTP (Roche, UK) and TdT for 1 hour at 37°C. Residual biotin-16-dUTP was removed by washing with Buffer D (100mM Tris-HCl pH7.4, 150mM NaCl). Nuclei were fixed by the addition of 1% formaldehyde and biotin-labelled ends were immunopurified using an anti-biotin antibody (Sigma-Aldrich, UK). DNA was prepared as for the ChIP assay. Real-time PCR analysis was performed using primers listed below.

Primer		***PCR Primer Sequence***	***PCR Product (hg19)***	***ERE Location***	***ERE sequence***
TFF1 B/C (-391/-240)	F	CCCGTGAGCCACTGTTGT	chr21:43,787,017 - 43,787,210	chr21:43,787,036 - 43,787,050	AGGTCACGGTGGCCA
	R	ATGGGAGTCTCCTCCAACCT			
TFF1 A/C (-567/-240)	F	TTAAGTGATCCGCCTGCTTT	chr21:43,786,884-43,787,210	as above	
	R	ATGGGAGTCTCCTCCAACCT			
GREB1	F	GAAGGGCAGAGCTGATAACG	chr2:11,672,533 - 11,672,663	chr2:11,672,659 - 11,672, 673	GGGTCATTCTGACCT
	R	GACCCAGTTGCCACACTTTT			
EGR3	F	GCCATCTGAGGCTTGAAAAG	chr8:22,592,462 - 22,592,611	chr8:22,592,668 - 22,592,682	AGGTCATGGTATAAG
	R	CCCATCTCACCAGCTACCAT			
PDZK1	F	AGGCCCAGCAAAGACAAATG	chr1:145,726,657 - 145,726,743	chr1:145,726,711 - 145,726,725	AGGTCACCCAGTCCT
	R	AAACCACAGGCTGAGGACTG			
SCN2A1	F	AGAGTGGGCTTTGGTTTCCT	chr2:166,094,638 - 166,094,813	Not applicable	
	R	CCATCAGAAGCCAAAATGCT			

3D-PCR, Cloning and Sequencing

PCR primer details for 3D-PCR are given below.

TFF1		***Sequence***
1st round PCR	Forward	TTAAGTGATCCGCCTGCTTT
	Reverse	AGCCCCGGATTTTATAGGG

2nd round PCR	Forward	TGATCCGCCTGCTTTGGCCT
	Reverse	CCGGATTTTATAGGGCAGGC
PDZK1		***Sequence***
1st round PCR	Forward	GCAACCTCTGCCTCTCAATC
	Reverse	GTCCCAGGCTTCCTTCTTCT
2nd round PCR	Forward	AGACAAGGTTTTGCCATGTG
	Reverse	TCTTCCCCTTCACACACTCA

Analysis of breast cancer gene expression microarray data sets

A trancriptomic breast cancer database of publicly available microarrays was established as described previously (Gyorffy and Schafer, 2009). The entire database contains 5,935 breast cancer patients. In these patients, 4,659 have survival information, with the average follow-up for relapse-free survival = 68.7 months. 76% of the patients are ER-positive and 42% are lymph node positive. The gene chip files were MAS5 normalized using the affy Bioconductor library. Illumina microarrays generated by the Metabric consortium (Curtis et al., 2012) were processed in the R statistical environment (http://www.r-project.org). The database includes 1,988 patients, the average overall survival is 8.07 years, 76% of the patients are ER-positive and 47.3% are lymph node positive. The Illumina microarray files were processed using the beadarray package, the illuminaHumanv3 database for annotation and quantile normalization via the preprocessCore package (http://www.bioconductor.org).

Kaplan-Meier survival plots and the hazard ratios with 95% confidence intervals and logrank P-values were calculated and plotted in R, as previously described (Gyorffy et al., 2013). Statistical significance was set at $p<0.01$. In addition, Cox proportional hazard regression was performed to compare the association between gene expression, clinical variables and survival using WinSTAT 2013 for Microsoft Excel (Robert K. Fitch Software, Germany).

Supplemental Figure Legends

Figure S1. APOBEC3B expression is associated with poor patient survival in estrogen receptor-positive breast cancer, related to figure 1. (A, B) Kaplan-Meier plots of breast cancer survival for ER-positive breast cancer patients (Fig. 1A), ER-negative patients, or all patients, according to A3B expression in the METABRIC data set. **(C)** Multivariate Cox regression analysis (METABRIC), including A3B expression, nodal status, grade and tumor size. **(D-F)** KM plots of a database comprising public Affymetrix microarray data show association between A3B expression and poor outcome in ER-positive breast cancer. **(G)** RNA prepared from breast cancer cell lines was assessed for APOBEC gene expression by quantitative RT-PCR. Boxplots show expression of all family members. APOBEC3B is the most highly and widely expressed family member, APOBEC3C being the other family member expressed widely in breast cancer cells. Expression of all other family members is 10-1000 times lower than that of APOBEC3B in the vast majority of lines. **(H-R)** Shown is the mRNA expression of APOBEC family members in each breast cancer cell line, which are summarized in (G). The order in which the cell lines are shown is based on the APOBEC3B expression profile, ranging from lines with the least APOBEC3B expression (left) to highest APOBEC3B expression (right) (H).

Figure S2. APOBEC3B regulates ER target gene expression in MCF-7 cells, related to figure 1. (A) Hormone-depleted MCF-7 cells were transfected with two independent siRNAs for A3B, or with a control siRNA. Cell growth in the presence of 10 nM estrogen or vehicle control was measured using the sulphorhodamine B (SRB) assay and is shown relative to growth on day of transfection (day 0; n=5). **(B)** MCF-7 cells were transfected with A3B siRNAs. Forty-eight hours later, estrogen (10 nM) was added and RNA prepared a further 12 hours later. Quantitative RT-PCR was undertaken and gene expression was corrected for GAPDH levels and is shown for the estrogen treated samples, relative to expression of each gene in the vehicle control (n=3). **(C)** Hormone-depleted MCF-7 cells were transfected with two independent A3B siRNAs or with control siRNA. Estrogen (10 nM) was added

after 48 hours and protein lysates prepared a further 12 hours later were immunoblotted. ß-actin served as a loading control. **(D)** SkBr3 cells, which are null for A3B, were transfected with A3B siRNAs. There was no effect on SkBr3 cell growth (n=5). **(E)** MDA-MB-231 ER-negative breast cancer cells that express A3B were treated with A3B siRNAs and growth was assessed over 4 days (n=4) The bar chart shows real-time RT-PCR for A3B following siRNA transfection (n=3). **(F)** Hormone-depleted MCF-7 cells were transfected with HA-A3B. After 24 hours, the cells were transfected with A3B siRNAs targeting the coding region (ORF), or with a siRNA that targets the A3B 3'-untranslated region (3'UTR). A further 24 hours later, estrogen (10 nM) was added for 12 hours. Real-time RT-PCR results are shown for A3B and ER target genes, TFF1, PDZK1 and PGR (n=3). *=p<0.01 for a comparison of siControl transfected cells with siA3B transfected cells, comparisons being within the vector or HA-A3B transfected samples. # denotes significant differences (p<0.01) for siControl, siA3B (3'UTR), siA3B#1 or siA3B#2 for equivalent vector and HA-A3B transfections. **(G)** Immunoblotting for A3B and HA tags demonstrates expression of ectopic and endogenous A3B.

Figure S3. Recruitment of APOBEC3B to chromatin is estrogen receptor dependent, related to figure 2. (A) ChIP assay for the ER binding region of the GREB1 gene, using lysates prepared from hormone-depleted MCF-7 cells treated with estrogen for 45 minutes, as for the TFF1 gene in Fig. 2C. **(B)** Details of A3B ChIP-sequencing runs are shown. **(C)** Genomic distribution of binding regions from A3B ChIP-seq in estrogen-treated cells. **(D)** MCF-7 cells were treated with Fulvestrant (FUL) for 24 hours. Estrogen was added 45 minutes prior to ChIP lysate preparation. ChIP was performed for the ER binding region of the PDZK1 gene, as for the TFF1 gene in Fig. 2E. Immunoblotting confirms that fulvestrant treatment downregulates ER. Note that fulvestrant treatment does not influence A3B levels. **(E)** ChIP was performed from MCF-7 lysates prepared following transfection with siA3B or siControl.

Figure S4. APOBEC3B mediates C-to-T transitions in the PDZK1 ER binding region in T47D cells, related to figure 3.

(A) genome browser snapshot of A3B, γH2AX and ER ChIP-seq. **(B)** Real-time RT-PCR was carried out using RNA prepared from T47D cells transfected with siControl or siA3B (n=3). **(C)** agarose gel analysis of 3D-PCR amplicons from T47D cells transfected with control or A3B siRNAs using primers flanking the PDZK1 ER binding region. Asterisks denote p<0.001. **(D)** mutation analysis of 3D-PCR amplicons was carried out by cloning and sequencing of PCR products (n>45). C-to-T changes were found in 19/56 (33.3%) of clones from siControl cells, compared with 4/47 (8.5%) of clones from siA3B transfected T47D cells. **(E)** Positions of C-to-T changes identified from cloning of 3D-PCR amplicons are depicted as blue dots. Dots above the red bar show C-to-T changes on the coding strand, those below the red bar are C-to-T changes on the template strand. The region sequenced maps between bp 145,726,380 and 145,726,873 (hg19). **(F)** Hormone-depleted T47D cells were transfected with UNG siRNA, or with siControl. Cells were treated with 10 nM estrogen after 48 hours and RNA prepared after a further 12 hours. Real-time PCR analysis for three replicates is shown. **(G)** Hormone-depleted T47D cells were transfected with myc-tagged UGI. Estrogen was added after 24 hours. RNA was prepared after a further 12 hours and protein lysates after 24 hours estrogen treatment. Real-time PCR is shown for n=3. * = p<0.001.

Figure S5. Estrogen treatment results in rapid and transient induction of γH2AX, related to figure 4. **(A)** Hormone-depleted MCF-7 cells were treated for 10 minutes with estrogen, diethylstilbesterol (DES) or the selective estrogen receptor-α agonist PPT. Cells were fixed and immunostained with antibodies for γH2AX and nuclei were visualised by staining with TOPRO DNA stain. **(B)** The number of γH2AX foci per cell were quantified using Cell Profiler 2.0. The bar chart shows the mean number of γH2AX foci in 100 cells from five replicates (total n=500). **(C)** Hormone-depleted MCF-7 cells were treated with vehicle, estrogen (10 nM), 4-hydroxytamoxifen (OHT; 100 nM) or Fulvestrant (FUL; 100 nM) for 10 minutes. For the H_2O_2 (10 mM) treatment, cells were fixed 45 minutes after the addition of H_2O_2. **(D)** hormone-depleted MCF-7 cells were transfected with A3B or ER siRNAs and immunostained for

γH2AX following 10 min estrogen treatment. **(E, F)** Estrogen, OHT or fulvestrant were added to T47D cells. The cells were fixed after 10 minutes and immunostained for γH2AX. The bar chart shows quantification for γH2AX foci number per cell for 100 cells (n=5 for a total of 500 cells). **(G)** Hormone-depleted MCF-7 cells were pre-treated with vehicle, a DNA-PK inhibitor (Nu7441; 5μM) or ATM inhibitor (KU55933; 5μM), for 1 hour and then vehicle or estrogen for 10 minutes. The bar chart shows quantification of a total of 500 cells from 5 replicates. **(H)** Estrogen-treated MCF-7 cells were immunostained for γH2AX (green) and 53BP1 (red). Fluorescence intensity overlap was assessed using the Carl Zeiss LSM 510 software to demonstrate co-localisation. **(I)** hormone-depleted MCF-7 cells were transfected with an UNG siRNA and immunostained for γH2AX following 10 min estrogen treatment.

Figure S6. Ectopic expression of the estrogen receptor in MDA-MB-231 cells facilitates APOBEC3B-dependent γH2AX induction and promotes expression of ER target genes, related to figure 4. (A) Treatment of the ER-negative MDA-MB-231 cells cultured in hormone-depleted culture medium, with estrogen for 10 minutes did not induce γH2AX. By contrast, estrogen treatment of two independent MDA-MB-231 derived lines stably expressing ER, caused γH2AX induction. **(B)** γH2AX induction was blocked by siA3B transfection in these lines. **(C)** Immunoblotting for A3B and ER following siA3B transfection shows A3B knockdown in MDA-MB-231 and ER expressing clones. **(D)** ER expression in MDA-MB-231 stimulates expression of ER target genes in an estrogen-dependent manner. A3B knockdown inhibits expression of these ER target genes (n=3; *$p<0.001$; #$p<0.05$).

Figure S7. γH2AX ChIP-seq shows concordance with A3B and ER binding regions related to figure 4. (A) Details of γH2AX ChIP-sequencing runs. **(B)** Correlation coefficient values on the genome scale for γH2AX, A3B and ER using raw (Wig) data. ChIP-seq reads were normalized (wig file) and binned in windows of 100 kb where the average score was calculated. The data were used to calculate genome wide correlation using a Spearman's

correlation score. **(C)** Heat map showing clustered binding signal for regions enriched in γH2AX for all treatment conditions. The window represents ±2.5 kb regions from the centre of the γH2AX regions. **(D)** Public data for MCF-7 cells from GEO was downloaded and aligned to hg19 using bwa software with default parameters. The MCF-7 datasets for p300 (Zwart et al., 2011), H4K12ac (Nagarajan et al., 2015), H3K4me1 (Theodorou et al., 2013) and BRD4 (Nagarajan et al., 2015) have been described, as has the GRO-seq (Hah et al., 2013). Encode Project data for H3K27ac and H3K4me3 were also used (http://encodeproject.org). Consensus ER peak list was based on the peaks present in at least two out of three MCF7 ER ChIP-seq replicates from Ross-Innes *et al*. (Ross-Innes et al., 2012) and contained 58661 peaks. Promoter-associated ER peaks were defined as peaks that overlap 3000 bp promoter regions (3000 bp upstream of TSS). All the other ER peaks were considered as enhancer-associated peaks. Peaks were sorted based on ER intensity. Heatmaps were visualized using seqMiner v1.3 software.

Table S1. DNA Motif enrichment analysis, related to figures 2 and 4.

References

Bogerd, H.P., Wiegand, H.L., Doehle, B.P., and Cullen, B.R. (2007). The intrinsic antiretroviral factor APOBEC3B contains two enzymatically active cytidine deaminase domains. Virology *364*, 486-493.

Curtis, C., Shah, S.P., Chin, S.F., Turashvili, G., Rueda, O.M., Dunning, M.J., Speed, D., Lynch, A.G., Samarajiwa, S., Yuan, Y., *et al.* (2012). The genomic and transcriptomic architecture of 2,000 breast tumours reveals novel subgroups. Nature *486*, 346-352.

Gyorffy, B., and Schafer, R. (2009). Meta-analysis of gene expression profiles related to relapse-free survival in 1,079 breast cancer patients. Breast cancer research and treatment *118*, 433-441.

Gyorffy, B., Surowiak, P., Budczies, J., and Lanczky, A. (2013). Online survival analysis software to assess the prognostic value of biomarkers using transcriptomic data in non-small-cell lung cancer. PloS one *8*, e82241.

Hah, N., Murakami, S., Nagari, A., Danko, C. G., and Kraus, W. L. (2013). Enhancer transcripts mark active estrogen receptor binding sites. Genome research *23*, 1210-1223.

He, H.H., Meyer, C.A., Shin, H., Bailey, S.T., Wei, G., Wang, Q., Zhang, Y., Xu, K., Ni, M., Lupien, M.*, et al.* (2010). Nucleosome dynamics define transcriptional enhancers. Nature genetics *42*, 343-347.

Ji, X., Li, W., Song, J., Wei, L., and Liu, X.S. (2006). CEAS: cis-regulatory element annotation system. Nucleic acids research *34*, W551-554.

Ju, B.G., Lunyak, V.V., Perissi, V., Garcia-Bassets, I., Rose, D.W., Glass, C.K., and Rosenfeld, M.G. (2006). A topoisomerase IIbeta-mediated dsDNA break required for regulated transcription. Science *312*, 1798-1802.

Lai, C.F., Flach, K.D., Alexi, X., Fox, S.P., Ottaviani, S., Thiruchelvam, P.T., Kyle, F.J., Thomas, R.S., Launchbury, R., Hua, H.*, et al.* (2013). Co-regulated gene expression by oestrogen receptor alpha and liver receptor homolog-1 is a feature of the oestrogen response in breast cancer cells. Nucleic acids research *41*, 10228-10240.

Lopez-Garcia, J., Periyasamy, M., Thomas, R.S., Christian, M., Leao, M., Jat, P., Kindle, K.B., Heery, D.M., Parker, M.G., Buluwela, L.*, et al.* (2006). ZNF366 is an estrogen receptor corepressor that acts through CtBP and histone deacetylases. Nucleic acids research *34*, 6126-6136.

Nagarajan, S., Benito, E., Fischer, A., and Johnsen, S. A. (2015). H4K12ac is regulated by estrogen receptor-alpha and is associated with BRD4 function and inducible transcription. Oncotarget *6*, 7305-7317.

Reddy, S.M., Williams, M., and Cohen, J.I. (1998). Expression of a uracil DNA glycosylase (UNG) inhibitor in mammalian cells: varicella-zoster virus can replicate in vitro in the absence of detectable UNG activity. Virology *251*, 393-401.

Ross-Innes, C. S., Stark, R., Teschendorff, A. E., Holmes, K. A., Ali, H. R., Dunning, M. J., Brown, G. D., Gojis, O., Ellis, I. O., Green, A. R.*, et al.* (2012). Differential oestrogen receptor binding is associated with clinical outcome in breast cancer. Nature *481*, 389-393.

Schmidt, D., Wilson, M.D., Spyrou, C., Brown, G.D., Hadfield, J., and Odom, D.T. (2009). ChIP-seq: using high-throughput sequencing to discover protein-DNA interactions. Methods *48*, 240-248.

Theodorou, V., Stark, R., Menon, S., and Carroll, J. S. (2013). GATA3 acts upstream of FOXA1 in mediating ESR1 binding by shaping enhancer accessibility. Genome research *23*, 12-22.

Thiruchelvam, P.T., Lai, C.F., Hua, H., Thomas, R.S., Hurtado, A., Hudson, W., Bayly, A.R., Kyle, F.J., Periyasamy, M., Photiou, A.*, et al.* (2011). The liver receptor homolog-1 regulates estrogen receptor expression in breast cancer cells. Breast cancer research and treatment *127*, 385-396.

Zhang, Y., Liu, T., Meyer, C.A., Eeckhoute, J., Johnson, D.S., Bernstein, B.E., Nusbaum, C., Myers, R.M., Brown, M., Li, W.*, et al.* (2008). Model-based analysis of ChIP-Seq (MACS). Genome biology *9*, R137.

Zwart, W., Theodorou, V., Kok, M., Canisius, S., Linn, S., and Carroll, J. S. (2011). Oestrogen receptor-co-factor-chromatin specificity in the transcriptional regulation of breast cancer. The EMBO journal *30*, 4764-4776.

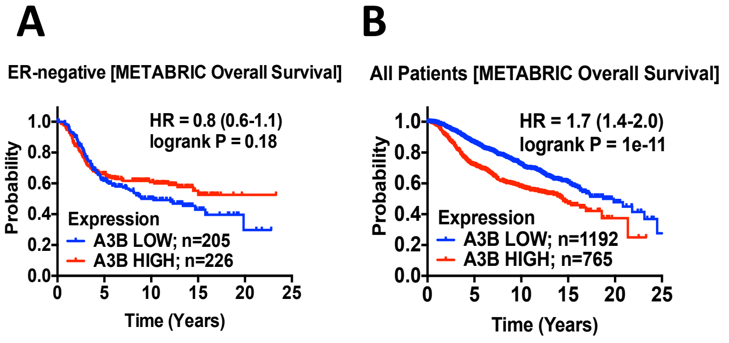

C

METABRIC	p-value	HR	Conf. (±)
APOBEC3B	1.27×10^{-6}	1.59	0.19
Lymph Node Status	4.49×10^{-7}	1.92	0.20
Tumor Grade	4.00×10^{-2}	1.18	0.16
Tumor Size	5.05×10^{-7}	1.02	0.00

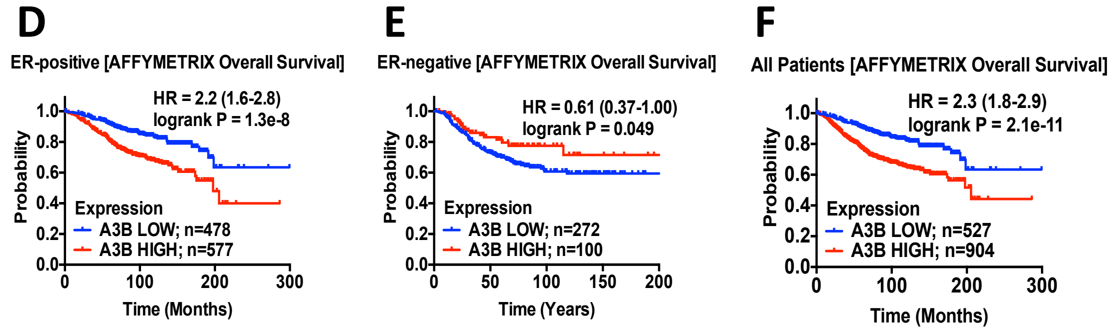

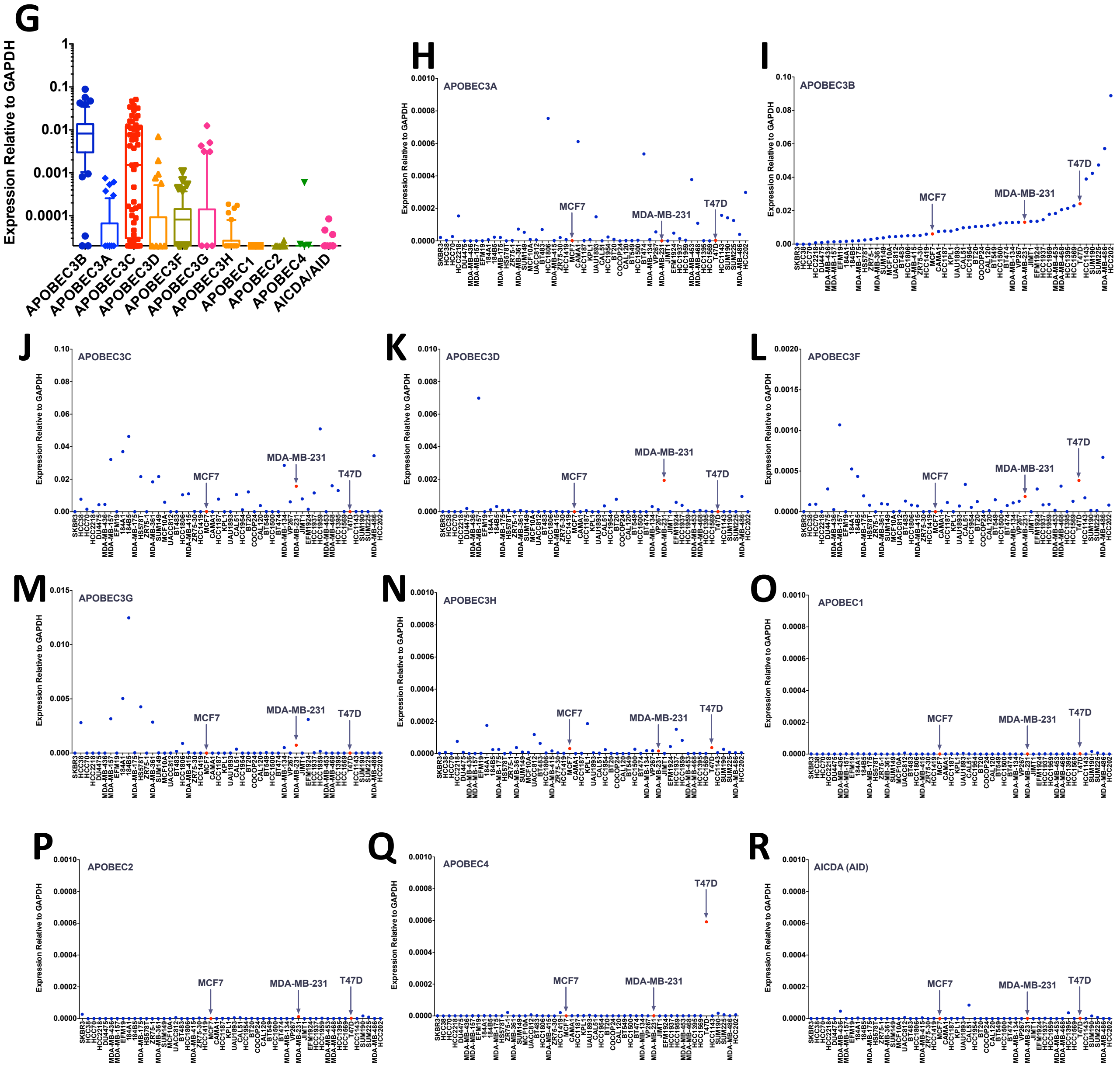

Figure S1 Periyasamy *et al*

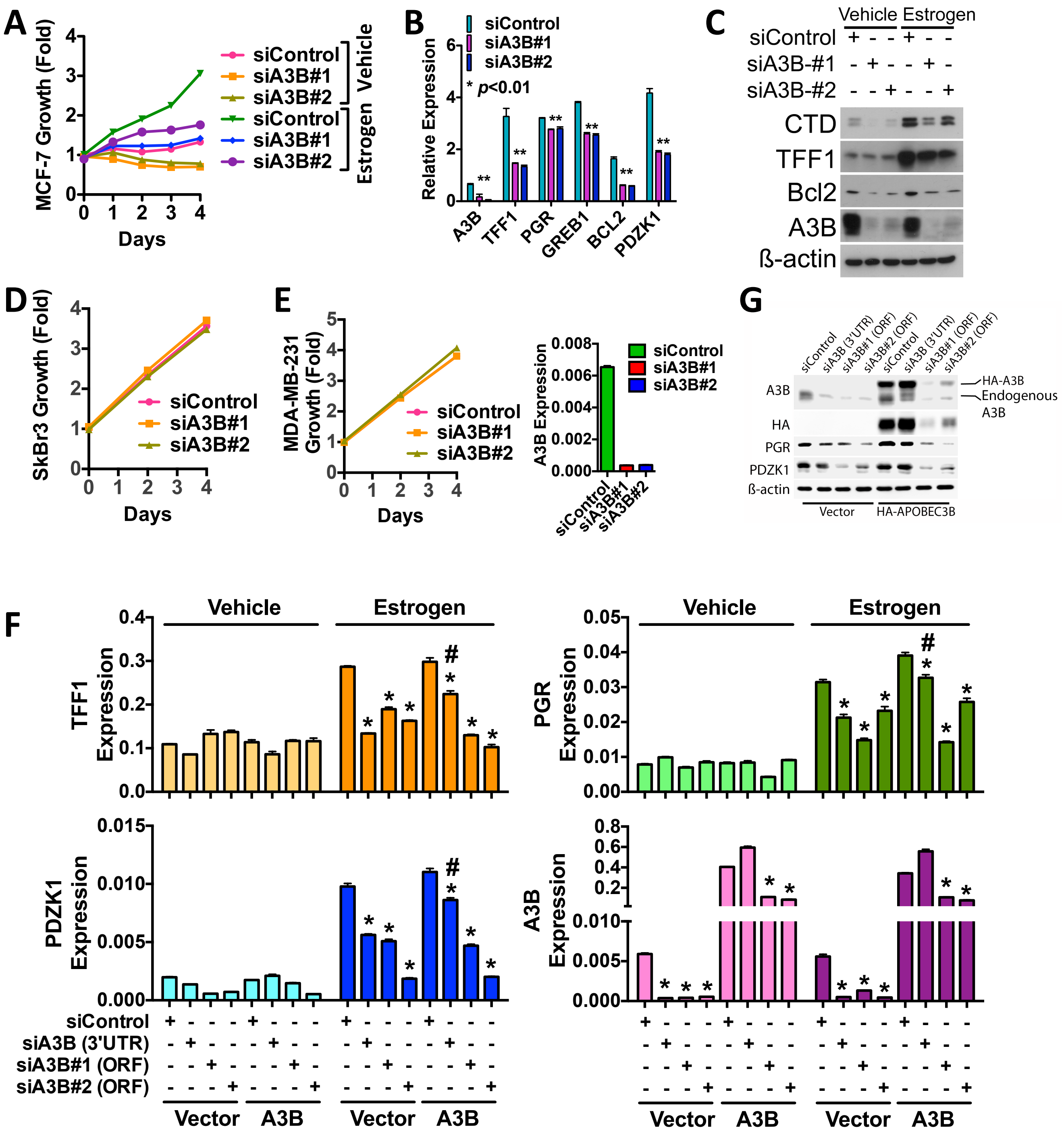

Figure S2 Periyasamy *et al*

A

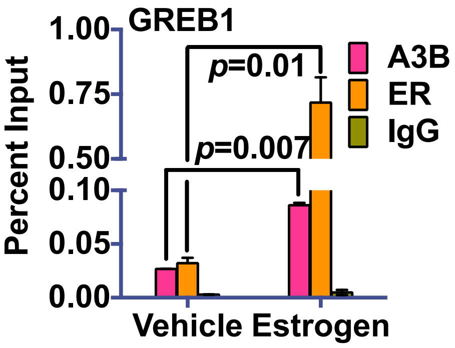

B

A3B ChIP-seq	Total Reads	Aligned Reads	% Aligned
Input Vehicle	18,032,282	15,180,049	84.2
A3B Vehicle	25,996,991	25,237,544	97.1
A3B Estrogen	32,913,006	31,895,248	96.9

C

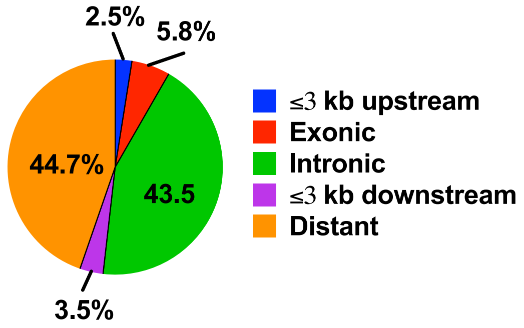

D

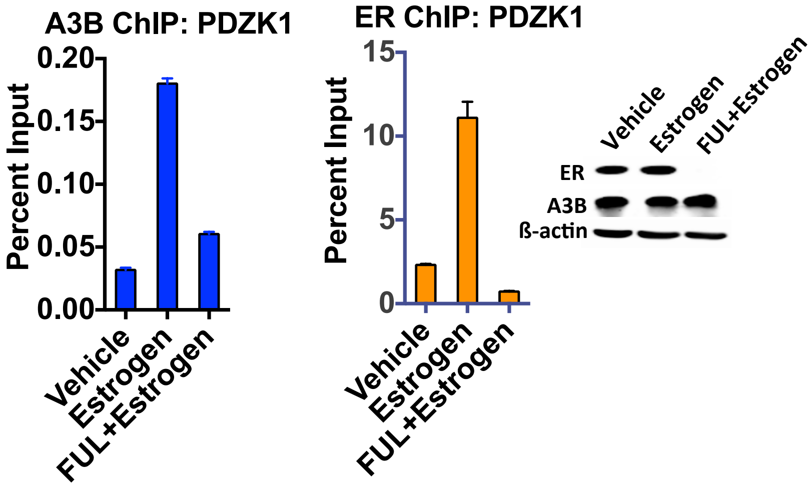

E

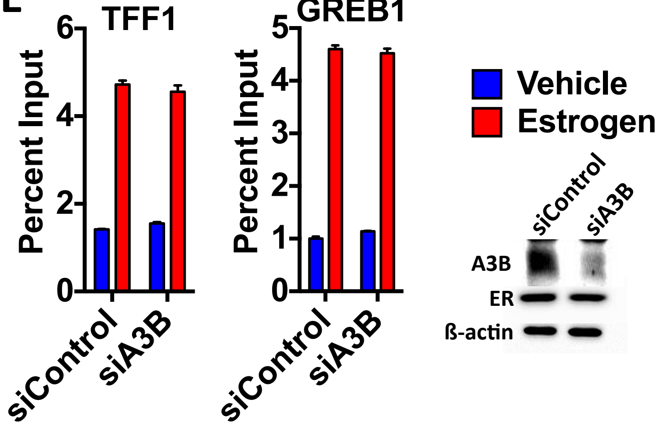

Figure S3 Periyasamy *et al*

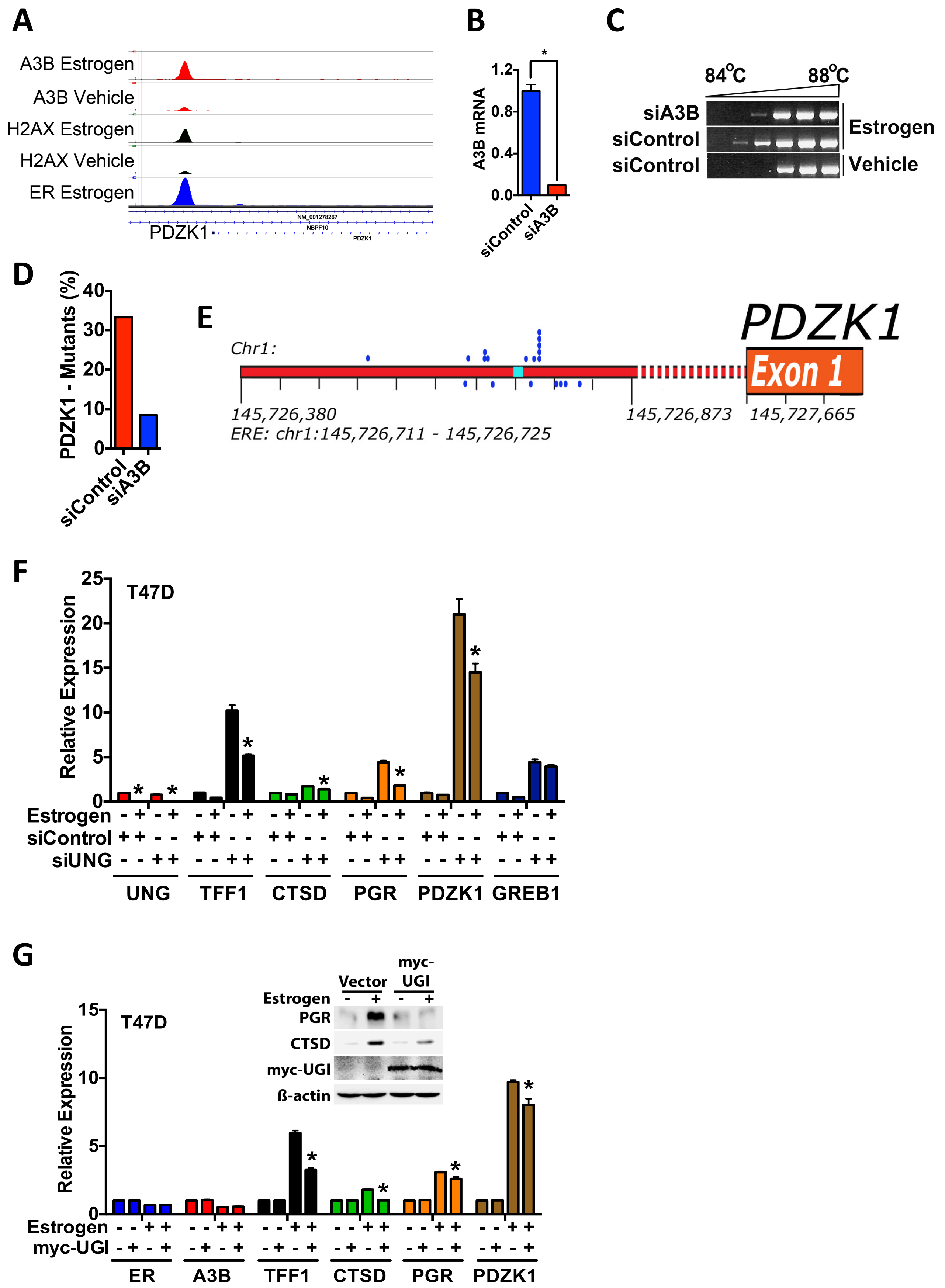

Figure S4 Periyasamy *et al*

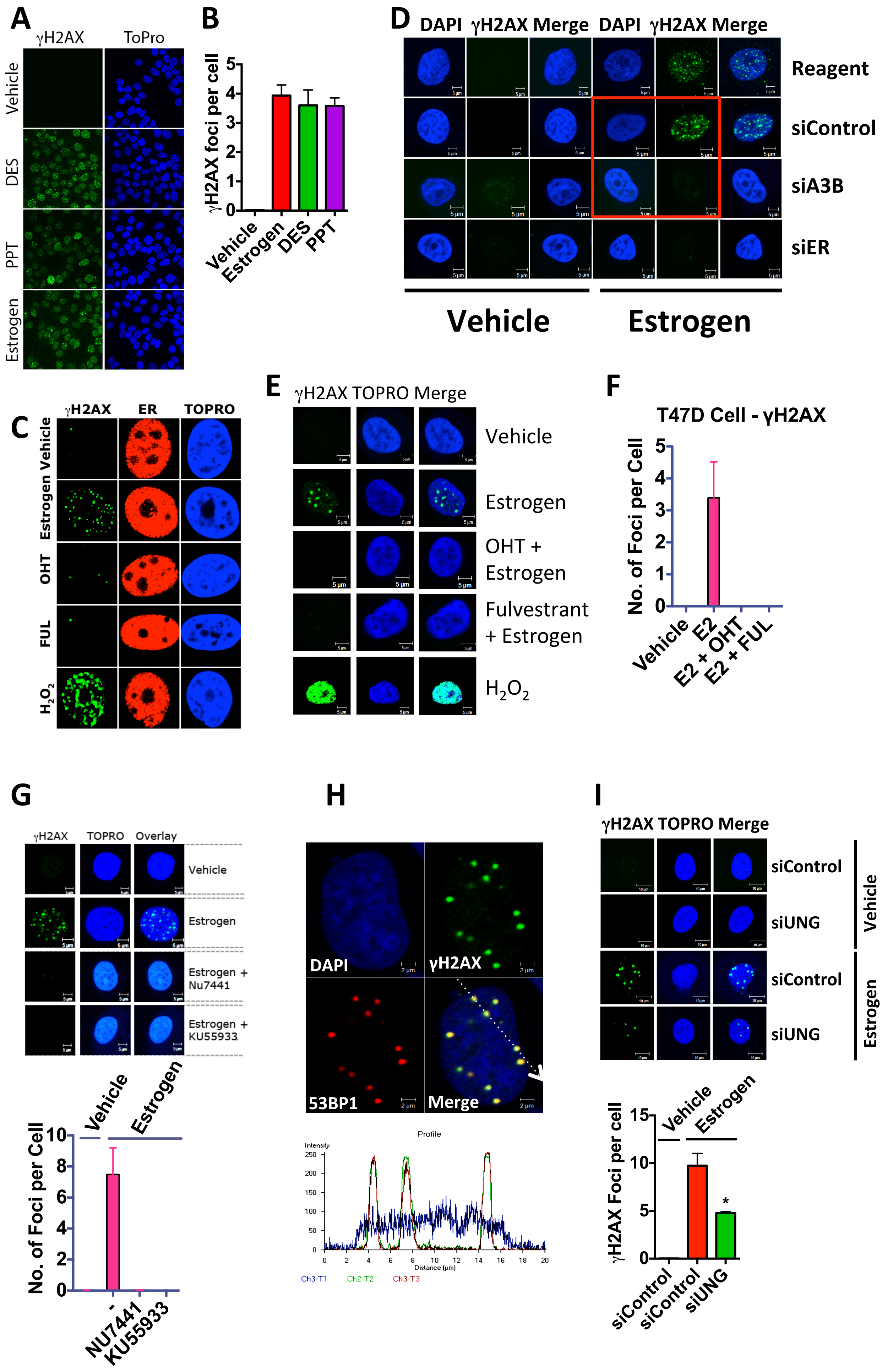

Figure S5 Periyasamy *et al*

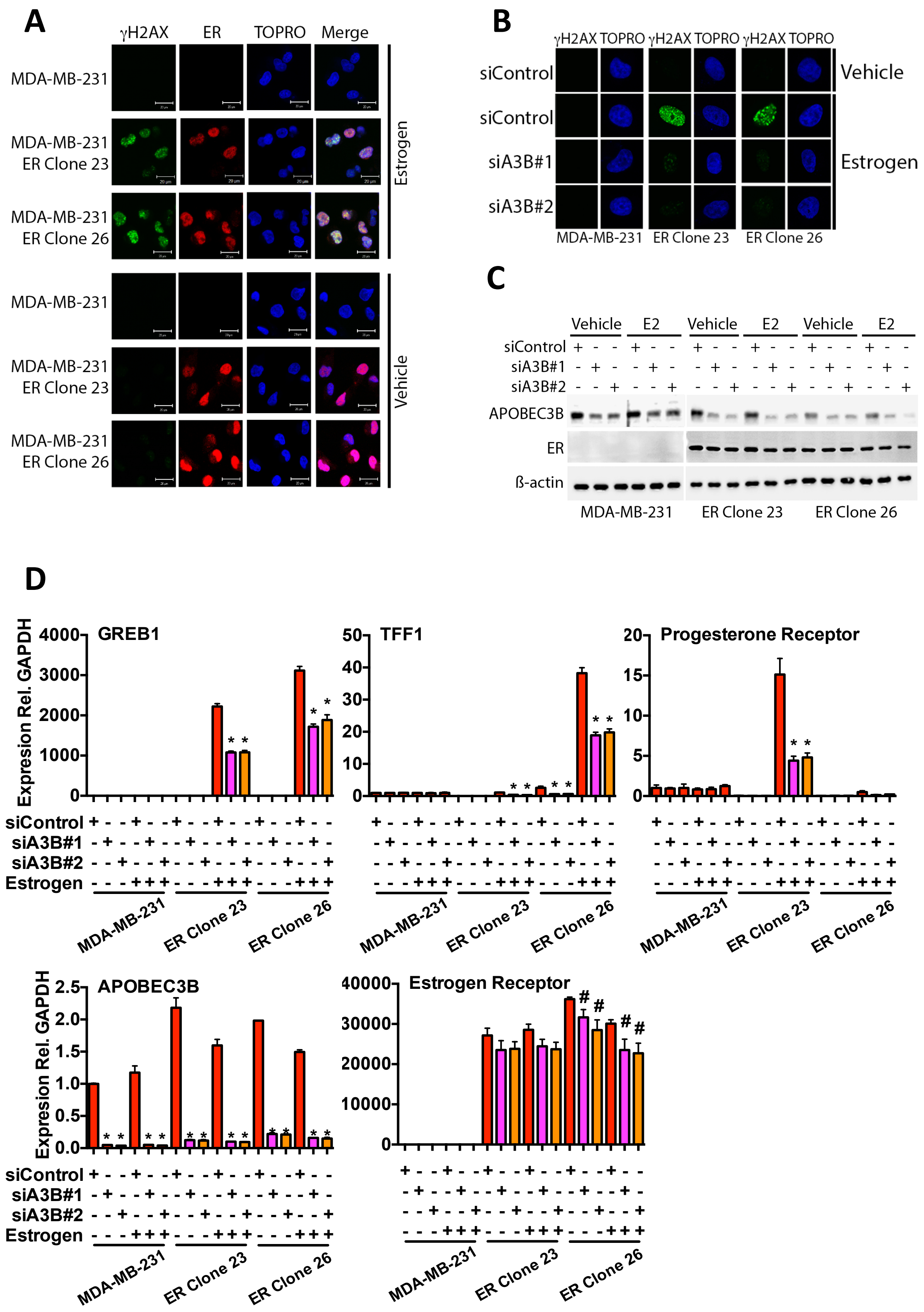

Figure S6 Periyasamy *et al*

A

γH2AX ChIP-seq	Total Reads	Aligned Reads	% Aligned
Vehicle	5,748,915	5,626,134	97.9
Estrogen	13,046,826	12,711,728	97.4
H_2O_2	7,057,147	6,944,363	98.4
H_2O_2/Estrogen	12,937,543	12,548,040	97.0

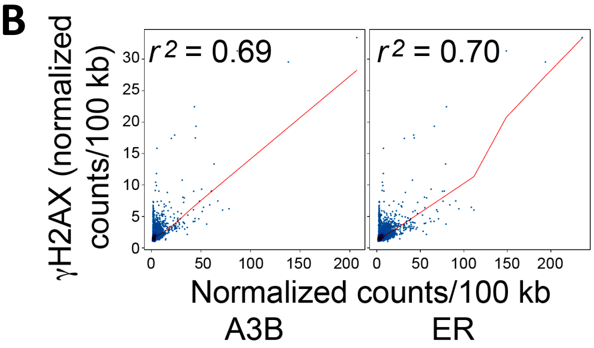

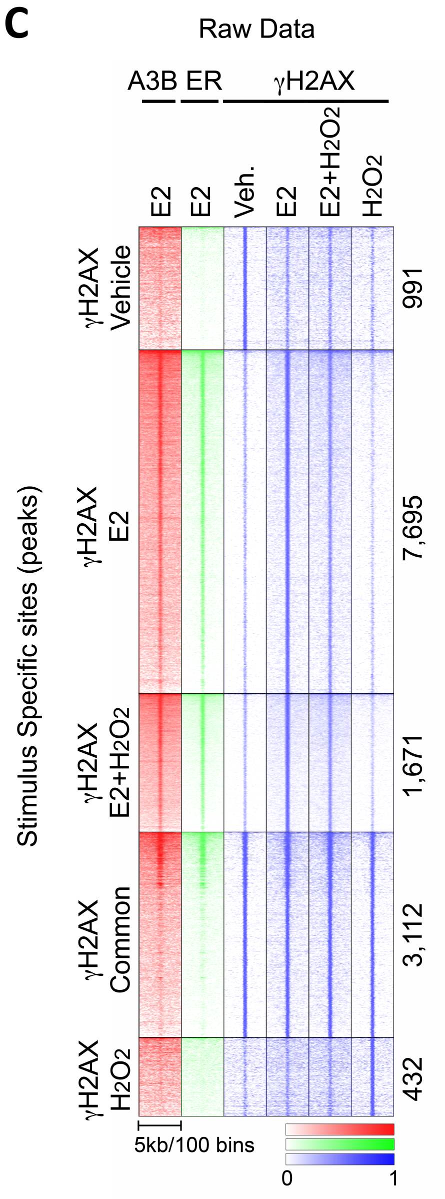

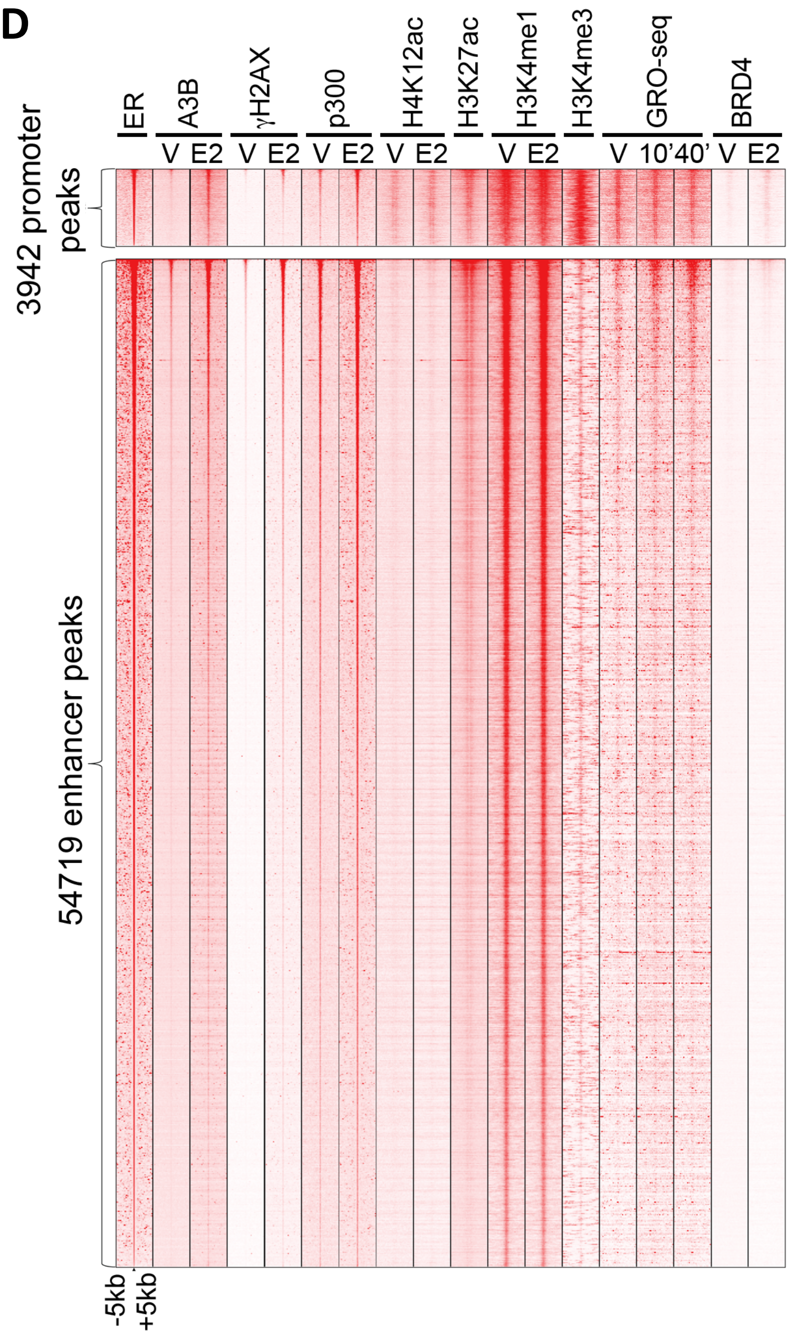

Figure S7 Periyasamy *et al*
