# Supplementary material for: APOBEC3B-Mediated Cytidine Deamination Is Required for Estrogen Receptor Action in Breast Cancer
Source: Cell Rep. 2015 Sep 24;13(1):108–21. doi: 10.1016/j.celrep.2015.08.066 (PMC4597099; doi:10.1016/j.celrep.2015.08.066)
Supplement: Document S2. Article plus Supplemental Information [file mmc3.pdf]

Cell Reports

Article

APOBEC3B-Mediated Cytidine Deamination Is Required for Estrogen Receptor Action in Breast Cancer

Graphical Abstract

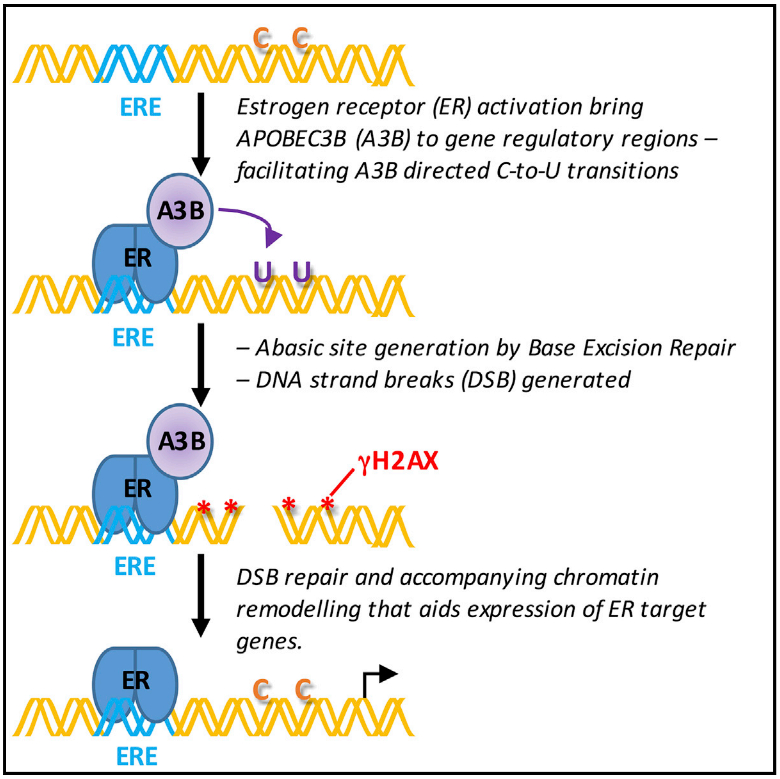

Authors

Manikandan Periyasamy, Hetal Patel, Chun-Fui Lai, ..., Luca Magnani, Laki Buluwela, Simak Ali

Correspondence

simak.ali@imperial.ac.uk

In Brief

Periyasamy et al. show that APOBEC3B is required for the regulation of gene expression by the estrogen receptor in breast cancer cells. They report APOBEC3B can promote cytidine deamination at gene regulatory regions, with consequent repair providing a mechanism for chromatin remodelling that facilitates gene expression.

Highlights

- APOBEC3B is associated with poor survival in ER+ breast cancer patients
- APOBEC3B controls breast cancer cell growth by promoting ER transcriptional activity
- APOBEC3B can cause C-to-U mutations at ER target genes, to activate DNA repair
- Repair of APOBEC3B-induced lesions allows chromatin remodelling that stimulates gene expression

Accession Numbers

GSE56979
GSE57426

Periyasamy et al., 2015, Cell Reports *13*, 108–121
October 6, 2015 ©2015 The Authors
http://dx.doi.org/10.1016/j.celrep.2015.08.066

OPEN ACCESS
CellPress

Cell Reports
Article

APOBEC3B-Mediated Cytidine Deamination Is Required for Estrogen Receptor Action in Breast Cancer

Manikandan Periyasamy,[1] Hetal Patel,[1] Chun-Fui Lai,[1] Van T.M. Nguyen,[1] Ekaterina Nevedomskaya,[2] Alison Harrod,[1] Roslin Russell,[3] Judit Remenyi,[4] Anna Maria Ochocka,[1] Ross S. Thomas,[1] Frances Fuller-Pace,[4] Balázs Győrffy,[5] Carlos Caldas,[3] Naveenan Navaratnam,[6] Jason S. Carroll,[3] Wilbert Zwart,[2] R. Charles Coombes,[1] Luca Magnani,[1] Laki Buluwela,[1] and Simak Ali[1,*]

[1]Department of Surgery and Cancer, Imperial College London, Hammersmith Hospital Campus, Du Cane Road, London W12 0NN, UK
[2]Department of Molecular Pathology, The Netherlands Cancer Institute, 1066 CX Amsterdam, the Netherlands
[3]Cancer Research UK, Cambridge Research Institute, Li Ka Shing Centre, Robinson Way, Cambridge CB2 0RE, UK
[4]Division of Cancer Research, University of Dundee, Ninewells Hospital and Medical School, Dundee DD1 9SY, UK
[5]MTA TTK Lendület Cancer Biomarker Research Group, Second Department of Pediatrics, Semmelweis University and MTA-SE Pediatrics and Nephrology Research Group, Budapest 1085, Hungary
[6]MRC Clinical Sciences Centre, Imperial College London, Hammersmith Hospital Campus, Du Cane Road, London W12 0NN, UK
*Correspondence: simak.ali@imperial.ac.uk
http://dx.doi.org/10.1016/j.celrep.2015.08.066
This is an open access article under the CC BY license (http://creativecommons.org/licenses/by/4.0/).

SUMMARY

Estrogen receptor α (ERα) is the key transcriptional driver in a large proportion of breast cancers. We report that APOBEC3B (A3B) is required for regulation of gene expression by ER and acts by causing C-to-U deamination at ER binding regions. We show that these C-to-U changes lead to the generation of DNA strand breaks through activation of base excision repair (BER) and to repair by non-homologous end-joining (NHEJ) pathways. We provide evidence that transient cytidine deamination by A3B aids chromatin modification and remodelling at the regulatory regions of ER target genes that promotes their expression. A3B expression is associated with poor patient survival in ER+ breast cancer, reinforcing the physiological significance of A3B for ER action.

INTRODUCTION

Estrogens play a central role in promoting breast cancer development (Ali and Coombes, 2002) and are important in uterine and ovarian cancer (O'Donnell et al., 2005; Shang, 2006). Two closely related nuclear receptors, estrogen receptor α (ERα, herein referred to as ER) and ERβ, mediate estrogen actions (Dahlman-Wright et al., 2006). ERα is dominant in breast cancer; 70% of breast cancers express ERα, and therapies to inhibit its activity have transformed breast cancer treatment. However, many patients develop resistance, with few treatment options being available for endocrine-therapy-resistant breast cancer (Osborne and Schiff, 2011).

Gene expression profiling and approaches for genome-wide identification of ER binding regions have allowed the identification of direct ER targets in breast cancer cells and highlight the importance of pioneer factors, particularly FOXA1 and GATA3 in directing ER by promoting chromatin accessibility and long-range chromatin interactions (Magnani et al., 2011; Ross-Innes et al., 2012). Critical for transcription regulation by ER is the ordered recruitment of a multitude of transcriptional co-regulator complexes with enzymatic activities for histone modification and chromatin remodeling (Métivier et al., 2006), which promote short- and long-range protein-DNA and protein-protein interactions between enhancer regions and gene promoters, to facilitate expression of ER target genes that drive breast cancer cell proliferation. The processes of transcription and DNA repair are intimately linked, as defined most obviously for the basal transcription factor TFIIH, which is essential for transcription initiation by RNA polymerase II (PolII), but is also required for the transcription-coupled nucleotide excision repair (Compe and Egly, 2012; Kamileri et al., 2012). Other DNA repair pathways also aid transcription (Fong et al., 2013) by promoting active DNA demethylation (Bhutani et al., 2011; Nabel and Kohli, 2011), enhancer RNA (eRNA) synthesis (Puc et al., 2015), and chromatin remodeling (Ju et al., 2006; Perillo et al., 2008), through processes that can involve the generation of single- and double-strand DNA breaks at enhancer regions.

The AID/APOBEC genes comprise a family of enzymes that mutate RNA or DNA by deaminating cytidine to uridine. Among their functions are RNA editing of the apolipoprotein B pre-mRNA by APOBEC1 and generation of antibody diversity by class-switch recombination and somatic hypermutation through DNA editing by AID (Conticello, 2008). In primates, there are seven closely related APOBEC3 genes, some of which function in retroviral restriction by promoting "hypermutation" in viral genomes. These functions do not readily explain the potential roles of APOBEC3 genes in non-immune system tissues, including breast, lung, cervix, bladder, and ovary, or the overexpression of some members of the family in cancers from these tissues (Burns et al., 2013a, 2013b; Leonard et al., 2013), although a potential role in the repair of DNA double-strand breaks (DSBs), resulting in resistance of lymphoma cells to ionizing radiation, has

been ascribed to APOBEC3G (Nowarski et al., 2012). Interestingly, ectopic expression of APOBEC3A (A3A) and A3B can promote mutagenesis in cancer cells (Burns et al., 2013a; Landry et al., 2011; Taylor et al., 2013).

Cancer genomes are marked by an accretion of somatic mutations. Recent whole-genome sequencing of breast cancer has yielded genome-wide mutational signatures, one of which is consistent with the DNA mutation profiles associated with cytidine deamination by APOBEC3 genes (Alexandrov et al., 2013; Nik-Zainal et al., 2012). Similar mutational signatures have been described in ovarian, bladder, cervical, head and neck, and lung cancer (Burns et al., 2013a, 2013b; de Bruin et al., 2014; Leonard et al., 2013; Roberts et al., 2013). A3B expression is frequently elevated in breast and other cancers that feature mutational landscapes consistent with cytidine deaminase activity (Burns et al., 2013a, 2013b). This, together with the demonstration that ectopic A3B expression can promote C-to-T mutations in breast cancer cells, has led to a proposed model in which A3B overexpression in breast cancer could aid tumor initiation and progression by driving somatic mutations in cancer. However, breast cancers from patients featuring a germline copy-number polymorphism involving A3A and A3B, in which A3B is effectively deleted, carry a greater burden of mutations associated with the APOBEC-dependent signature than those in which the A3A and A3B genes are intact (Nik-Zainal et al., 2014), bringing into question the importance of A3B in this process.

To investigate the potential role of A3B in breast cancer, we analyzed its expression in breast cancer. Surprisingly, A3B expression was associated with poor patient survival in ER+ breast cancer, but not in ER− breast cancer, despite the fact that A3B expression was higher in ER− breast cancer than in ER+ breast cancer, thus implicating A3B in ER action. Here, we provide evidence for a molecular mechanism in which A3B causes local and transient C-to-U transitions at ER enhancers, leading to activation of base excision repair (BER) and non-homologous end-joining (NHEJ) pathways, which in turn promote chromatin modification and remodeling, to drive expression of ER target genes.

RESULTS

A3B Expression Is Associated with Poor Prognosis in ER+ Breast Cancer

AID/APOBECs function in retroviral restriction and in the case of AID, in class-switch recombination and somatic hypermutation to generate antibody diversity by causing C-to-T mutations (Conticello, 2008), functions that do not explain the high-level expression of A3B in many cancers (Burns et al., 2013a, 2013b). Toward defining the role of A3B in breast cancer subtypes, we determined the relationship between A3B levels and patient outcome. Analysis of the METABRIC (Curtis et al., 2012) series of 2,000 breast cancer patients revealed that high A3B expression is associated with poor survival (hazard ratio [HR] = 1.5, $p = 1 \times 10^{-11}$) (Figures S1A and S1B). Interestingly, A3B expression was associated with poor outcome in ER+ (HR = 1.9; $p = 4.5 \times 10^{-11}$) (Figure 1A), but not in ER− (p = 0.18), breast cancer. A3B retained its significance (HR = 1.59, $p = 1.27 \times 10^{-6}$) in multivariate analysis of ER+ breast cancer (Figure S1C). There was no association between expression of other APOBECs and poor outcome in ER+ or ER− breast cancer in the METABRIC dataset (data not shown). We extended this analysis to other gene expression microarray datasets. Kaplan-Meier plot analysis of Affymetrix microarray datasets similarly showed that high A3B expression is associated with poor outcome for ER+, but not ER−, breast cancer (Figures S1D–S1F). Forest-plot analysis for relapse-free survival further confirmed the importance of A3B in ER+ breast cancer (Figure 1B). Real-time RT-PCR for 151 breast cancers again confirmed that A3B is expressed in ER+ and ER− breast cancer, as well as in the majority of breast cancer cell lines examined (Figures 1C and S1G–S1R), in agreement with previous findings (Burns et al., 2013a).

A3B Regulates the Growth of ER+ Breast Cancer Cells

Given the association between A3B expression and poor patient survival in ER+ breast cancer, we wondered whether A3B regulates the growth of ER+ breast cancer cells. To investigate this, we treated tumor xenografts of the ER+ and estrogen-regulated MCF7 breast cancer cell line, which expresses moderate levels of A3B, with A3B small interfering RNA (siRNA). A3B knockdown markedly inhibited MCF7 tumor growth (Figure 1D), demonstrating that A3B is required for MCF7 tumor growth in vivo. Remarkably, ER target gene expression was greatly reduced in these tumors (Figures 1E and 1F), suggesting that A3B impacts directly on ER function. Indeed, transfection of MCF7 cells in culture with two independent A3B siRNAs inhibited estrogen-stimulated growth, accompanied by reduced expression of ER regulated genes (Figures S2A–S2C). Inhibition of estrogen-regulated growth and estrogen-responsive gene expression was confirmed in a second ER+ cell line (T47D) (Figures 1G and 1H). SkBr3 cells, which are null for A3B (Komatsu et al., 2008), were not affected by A3B siRNA (Figures S1I and S2D). Growth of the A3B+/ER− MDA-MB-231 cells was also unaffected by A3B siRNA (Figure S2E). Furthermore, inhibition of ER target gene expression by a siRNA targeting the A3B 3′-UTR was rescued by transfection of an A3B expression plasmid that lacks the 3′ UTR. However, siRNAs targeting the A3B coding region knocked down endogenous and ectopic A3B and inhibited ER-regulated genes (Figures S2F and S2G). Taken together, these results demonstrate A3B specificity of the siRNAs and show that A3B regulates growth and expression of estrogen-responsive genes in breast cancer cells.

A3B Is Recruited Globally to ER Binding Regions, and This Requires Its Interaction with the ER

Next, we sought to determine whether A3B is required for the ER transcriptional response. In a reporter gene assay, A3B stimulated ER activity (Figures 2A and 2B). A3B contains two zinc coordinating cytidine deaminase activity (CDA) domains (Conticello, 2008). Substitution of glutamic acid residues at positions 68 or 255 to glutamine, which inhibits the cytidine deaminase activity in the N- and C-terminal domains of A3B, respectively, reduced stimulation of ER activity. Hence, both CDA domains are required for modulation of ER activity by A3B, echoing a previous report, which showed that both CDA domains are

OPEN
ACCESS
CellPress

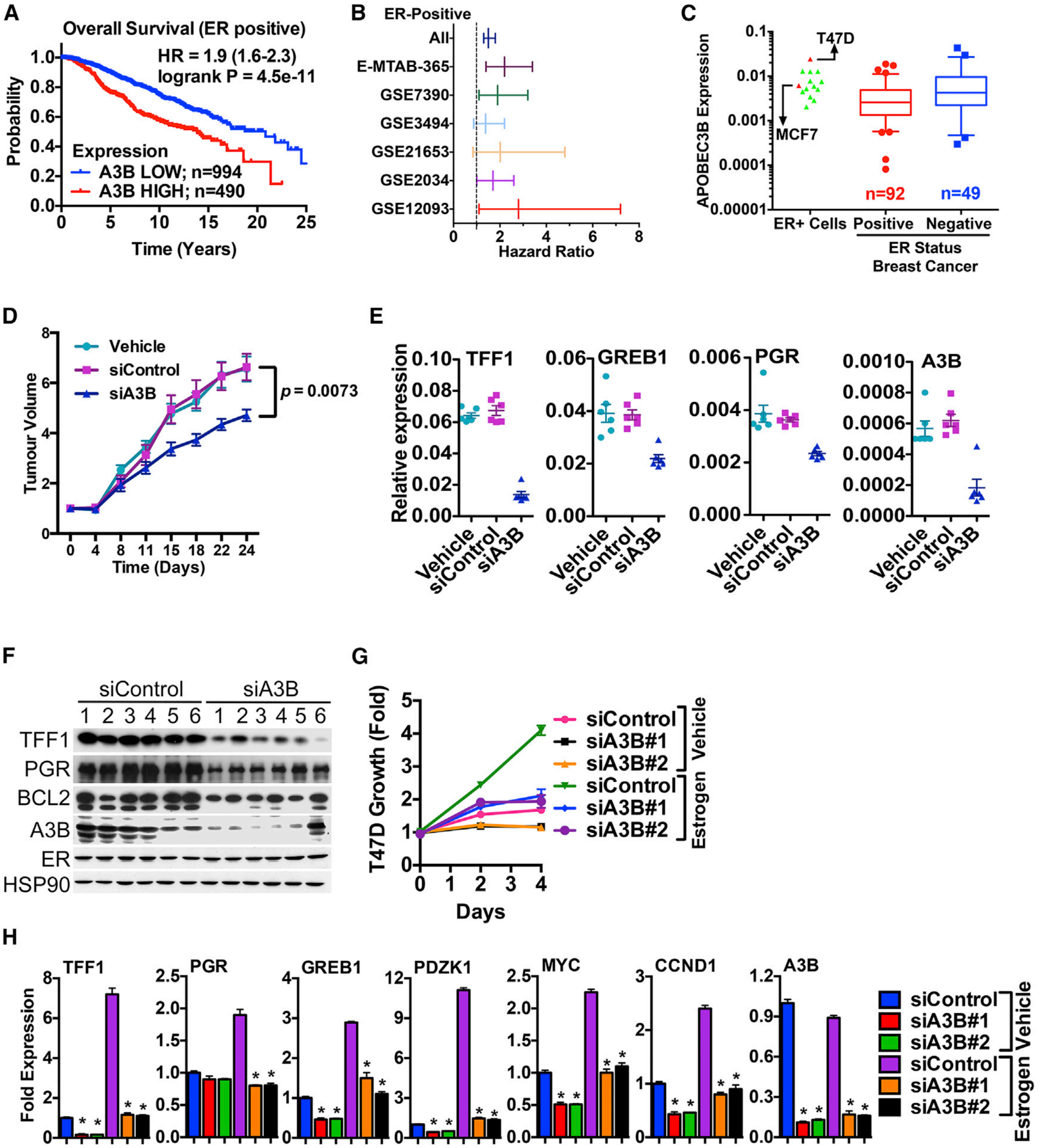

Figure 1. A3B Regulates ER Activity to Promote Breast Cancer Cell Growth

(A) Kaplan-Meier plots of breast cancer survival for ER+ patients from METABRIC, according to A3B expression.

(B) Forest plot analysis of ER+ breast cancers for A3B expression. Affymetrix microarray datasets that included >100 ER+ breast cancers were used in the analysis: E-MTAB-365 (Guedj et al., 2012), GSE7390 (Desmedt et al., 2007), GSE3494 (Miller et al., 2005), GSE21653 (Sabatier et al., 2011), GSE2034 (Wang et al., 2005), and GSE12093 (Zhang et al., 2009). Hazard ratios and 95% confidence intervals are plotted on the x axis.

(C) Real-time RT-PCR was carried out using RNA prepared from ER+ (n = 92) and ER− (n = 49) breast cancers. Also shown are the expression profiles for ER+ breast cancer cell lines, with MCF7 and T47D expression highlighted.

(D) MCF7 tumors were treated weekly with vehicle (n = 6), control siRNA (n = 6), or A3B siRNA (n = 6). Mean tumor volumes are plotted ± SEM. RNA and protein were prepared from tumors at the end of the experiment.

(E) Real-time RT-PCR relative to GAPDH levels (n = 3; $p < 0.0001$) for tumors.

(F) Immunoblotting of protein lysates (20 µg) tumors is shown.

(G) Hormone-depleted T47D cells transfected with A3B siRNAs were assessed for growth (n = 4).

(H) mRNA levels were determined following transfection of hormone-depleted T47D cells with A3B siRNAs. mRNA expression is shown relative to the expression for the siControl samples (n = 3). Statistical significance within each treatment group for each A3B siRNA relative to siControl is denoted by asterisks ($p < 0.001$).

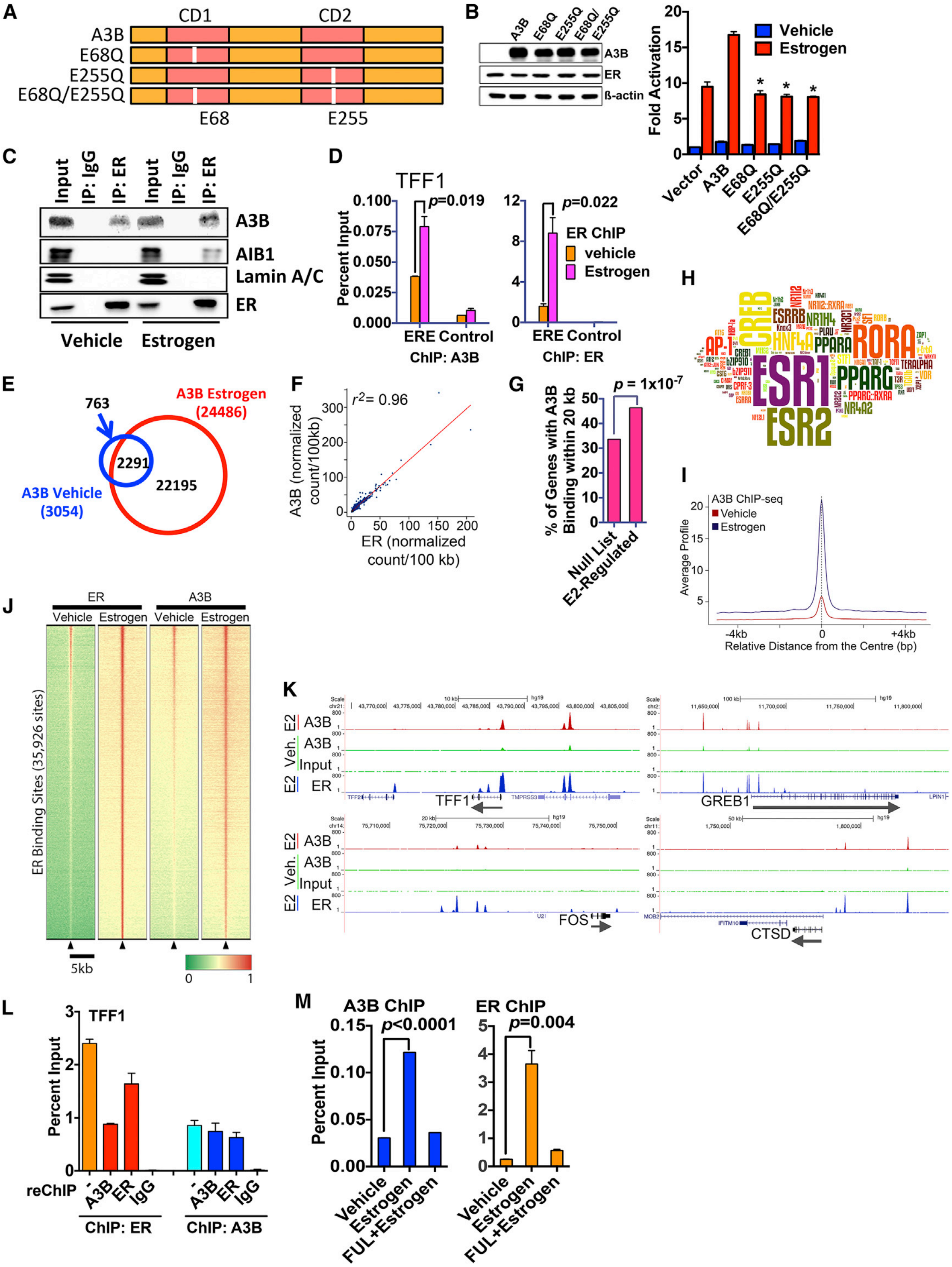

Figure 2. A3B Interacts with ER and Is Recruited to ER Binding Regions

(A) Schematic representation of A3B. Highlighted are the catalytic site mutations used here.

(B) COS-1 cells cultured in hormone-free medium were transfected with an estrogen-responsive luciferase reporter gene, ER and A3B. Estrogen (10 nM) was added for 20 hr. Data are shown as fold activation, relative to reporter activity for vehicle treatment, following co-transfection of ER without A3B (vector control) (n = 3; * = $p < 0.05$). Immunoblotting for ER and A3B following transfection of COS-1 cells is also shown.

(C) Hormone-depleted MCF7 cells treated with estrogen were immunoprecipitated with an ER antibody. Input represents 10% of lysate used in the immunoprecipitations.

(legend continued on next page)

enzymatically active and contribute to C-to-T editing (Bogerd et al., 2007). A3B was co-immunoprecipitated with ER in MCF7 cells (Figure 2C), and chromatin immunoprecipitation (ChIP) assays showed that A3B is recruited, in an estrogen-dependent manner, to the ER binding regions in the TFF1 and GREB1 genes (Figures 2D and S3A). AIB1, which interacts with ER in an estrogen-dependent manner (Anzick et al., 1997), acts as a control.

ChIP sequencing (ChIP-seq), for A3B to define the global distribution of A3B on chromatin, identified 24,486 binding sites in MCF7 cells treated with estrogen (Figures 2E and S3B). A3B binding was primarily observed at intronic and gene-distal regions (Figure S3C), suggesting that A3B binding occurs in gene regulatory regions. ChIP-seq for ER has established that the great majority of ER binding events also map to sites within introns and at considerable distances upstream and downstream of transcribed regions (Welboren et al., 2009). Analysis of the raw reads demonstrated a remarkably close genomic co-localization of the binding sites for the two proteins (r^2 = 0.96) (Figure 2F). A3B binding sites were significantly enriched in the vicinity of estrogen-responsive genes (Figure 2G) and were enriched in binding motifs for ER (Figure 2H; Table S1). Alignment of all ER binding sites showed that the majority of ER binding sites in MCF7 cells are bound by A3B and that estrogen treatment results in global stimulation of A3B recruitment to ER binding regions (Figures 2I and 2J), as evident from the genome browser snapshots for the ER target genes TFF1, GREB1, FOS, and CTSD (Figure 2K). These results provide a compelling argument for a mechanism of chromatin-based collaboration between A3B and ER, toward the global regulation of ER target genes in breast cancer.

Recovering the chromatin following ChIP for ER and performing ChIP with A3B antibody (ChIP/reChIP) showed that ER and A3B are present concurrently at the TFF1 ERE (Figure 2L). ChIP for A3B followed by reChIP for ER provided similar results. Treatment of MCF7 cells with the anti-estrogen fulvestrant (aka ICI182,780), which specifically promotes the downregulation of ER protein (McClelland et al., 1996), resulted in ER loss and lack of ER binding to chromatin in ChIP assays (Figures 2L and S3D). Fulvestrant did not affect A3B protein levels, but A3B recruitment to the TFF1 and PDZK1 ER binding regions was nevertheless prevented. By contrast, siRNA-mediated A3B knockdown did not affect ER recruitment (Figure S3E). Thus, although A3B interacts with ER in a ligand-independent manner, its recruitment to chromatin is estrogen dependent by virtue of estrogen-stimulated recruitment of ER to chromatin. Furthermore, given that A3B is not required for ER recruitment to DNA, A3B is unlikely to act as a pioneer factor.

A3B Promotes Cytidine Deamination to Generate C-to-U Transitions at ER Binding Regions in Breast Cancer Cells

Mutational inactivation of the A3B catalytic domains inhibited ER stimulation in reporter gene assays (Figure 2A), implying that deamination of deoxycytidine to deoxyuridine (C to U) is required for the regulation of estrogen-responsive gene expression by A3B. We used differential DNA denaturation PCR (3D-PCR), which identifies C-to-T changes, based on detecting PCR amplicons at lower denaturation temperatures arising from an increase in A/T content (Burns et al., 2013a; Suspène et al., 2005), to determine if A3B causes C-to-U changes at ER binding regions. Estrogen treatment of MCF7 cells generated lower-temperature amplicons in the TFF1 promoter region (Figure 3A). Cloning and sequencing of the PCR products from three independent experiments identified C-to-T changes in a total of 12/109 (11%) clones from vehicle-treated cells, increasing to 43/114 (38%) following estrogen treatment (Figure 3B). Importantly, the overwhelming majority (37/43 [86%]) of these changes mapped to the A3B binding region (Figure 3C). Sequencing of 3D-PCR products following A3B knockdown identified C-to-T changes in a total of 58/155 (37%) clones in siControl-transfected, compared with 13/163 (8%) clones in siA3B-transfected, MCF7 cells (Figures 3D and 3E), demonstrating that A3B is necessary for the C-to-U transitions as the TFF1 ER/A3B binding region. Similar results were obtained for the PDZK1 ER/A3B binding region, if A3B was knocked down in T47D cells (Figures S4A–S4E).

Failure to repair cytidine deamination would result in the accumulation of deleterious mutations at gene enhancers. As dU is excised by uracil DNA glycosylase (UNG) (Stavnezer, 2011), we determined if UNG is required for the A3B-dependent cytidine deamintion at ER binding regions. UNG knockdown

(D) MCF7 cells were treated with estrogen (10 nM, 45 min), followed by ChIP. Shown are the results of real-time PCR of ChIP DNA for the TFF1 gene promoter proximal ERE or a control region in the TFF1 gene to which ER binding is not observed (n = 3).
(E) A3B ChIP-seq was carried out following treatment of MCF7 cells with 10 nM estrogen for 45 min. The Venn diagram shows A3B binding regions from the peak-calling analysis.
(F) Genome-wide enrichment correlation analysis for A3B and ER raw signals demonstrates correlation (r^2 = 0.96) between A3B and ER binding sites. A3B and ER ChIP-seq reads were normalized (wig file) and binned in windows of 100 kb where the average score was calculated. The data were then used to calculate genome-wide correlation using a Spearman's correlation score. The score for each window is plotted and an interpolation line added to ease interpretation.
(G) Analysis of A3B binding regions identified by ChIP-seq are significantly enriched in the proximity of estrogen responsive genes.
(H) Analysis of the relative enrichment of transcription factor binding sites was used to generate a *Z* score, which is represented by the size of the motif in the word cloud.
(I) Average signal intensity of A3B ChIP-seq binding events, centered on ER binding regions, shows increased recruitment of A3B to ER binding regions globally, upon estrogen treatment. Signal intensity is a normalized count of individual, non-redundant ChIP fragments at single ER binding sites identified by the peak-calling algorithm (MACS 1.4)
(J) Heatmap showing clustered binding signal for A3B ± estrogen. The window represents ±2.5-kb regions from the center of the ER binding events. The color scale represents relative enrichment based on raw signal.
(K) Representative genome browser snapshots show A3B binding regions and overlap with ER binding regions, shown on the same scale.
(L) ChIP with ER or A3B antibodies (denoted by −) was followed by recovery of the chromatin complexes and reChIP with antibodies for A3B, ER, or mouse immunoglobulins (IgG control) (n = 3).
(M) MCF7 cells were treated with fulvestrant for 24 hr, followed by addition of estrogen for 45 min (n = 3).

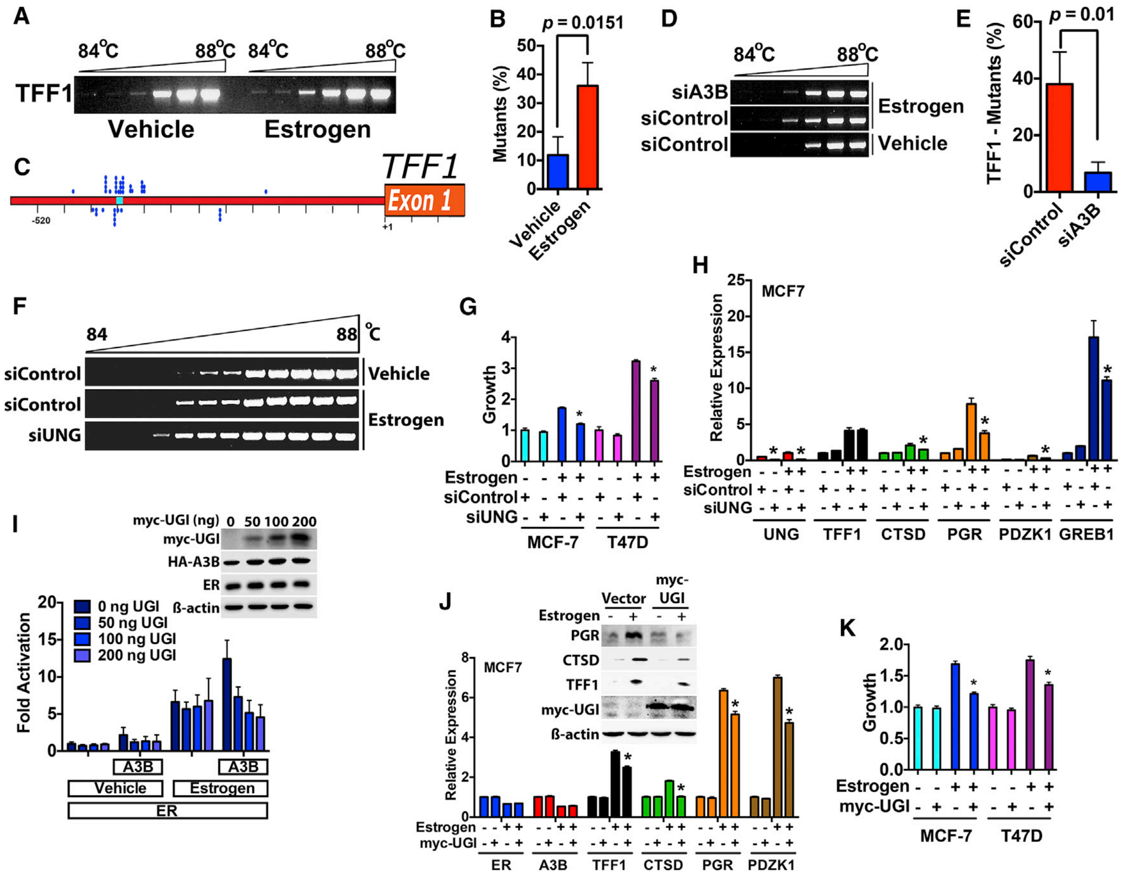

Figure 3. Estrogen Treatment Induces A3B-Dependent C-to-U Transitions at the ER and A3B Binding Region in the TFF1 Gene

(A) Genomic DNA was prepared from MCF7 cells treated with 10 nM estrogen for 45 min. Agarose gel analysis of 3D-PCR for the TFF1 promoter region is shown.

(B) Mutation analysis of 3D-PCR amplicons from vehicle- and estrogen-treated cells is represented as a percentage of clones harboring C-to-T changes. Clones from three independent experiments were sequenced, for a total of >100 clones per treatment.

(C) Each identified C-to-T transition is shown (blue dots), mapped to the region of the TFF1 gene (−520 to +80) amplified by 3D-PCR.

(D) MCF7 cells transfected with A3B or control siRNA were treated with estrogen as described above.

(E) 3D-PCR amplicons were cloned. Shown are the percentage of clones harboring C-to-T transitions for a total of >150 clones generated from three experiments.

(F) 3D-PCR of genomic DNA from MCF7 cells transfected with UNG siRNA were treated with estrogen.

(G) Hormone-depleted MCF7 and T47D cells transfected with siUNG were assessed for growth in the presence or absence of estrogen (*$p < 0.001$; n = 4).

(H) Real-time PCR using RNA prepared from MCF7 cells transfected with siUNG, following 12 hr treatment with estrogen (10 nM) (*$p < 0.001$; n = 3).

(I) HeLa cells cultured in hormone-free medium were transfected with an estrogen-responsive luciferase reporter gene, together with ER, A3B, and myc-UGI (n = 3). Immunoblotting is shown in the inset.

(J) MCF7 cells transfected with myc-UGI were treated with estrogen for 12 hr, and RNA and protein were isolated. Real-time PCR and immunoblotting for ER target genes are shown.

(K) Hormone-depleted MCF7 and T47D cells transfected with myc-UGI were grown in the presence or absence of estrogen for 5 days. Growth was measured using the sulforhodamine B (SRB) assay (*$p < 0.001$; n = 4).

resulted in lower-temperature amplicons at ER/A3B binding regions (Figure 3F), suggesting that lack of UNG "fixes" the A3B directed C-to-U changes in DNA. The consequence of UNG knockdown was inhibition of ER target gene expression and MCF7 and T47D cell growth (Figures 3G, 3H, and S4F).

We used the bacteriophage PBS2 uracil DNA glycosylase inhibitor (UGI), which represses UNG activity in mammalian cells (Burns et al., 2013a), to confirm the involvement of UNG. In a reporter gene assay, ectopic expression of UGI prevented the stimulation of ER activity by A3B (Figure 3I). UGI transfection also repressed endogenous ER target gene expression and estrogen-stimulated growth of MCF7 and T47D cells (Figures 3J, 3K, and S4G). Finally, ChIP showed that UNG was recruited to ER binding regions in the TFF1 and PDZK1 genes, its recruitment being prevented by A3B knockdown (Figures 4A–4C). Thus, UNG is required for ER function and is recruited to ER binding sites in an A3B-dependent manner. Importantly, these results indicate that repair of A3B driven cytidine deamination involves the action of UNG.

Estrogen Binding to ER Promotes DNA Strand Breaks at ER Binding Regions

The mechanism of immunoglobulin gene class switch recombination involves cytidine deamination by AID and subsequent

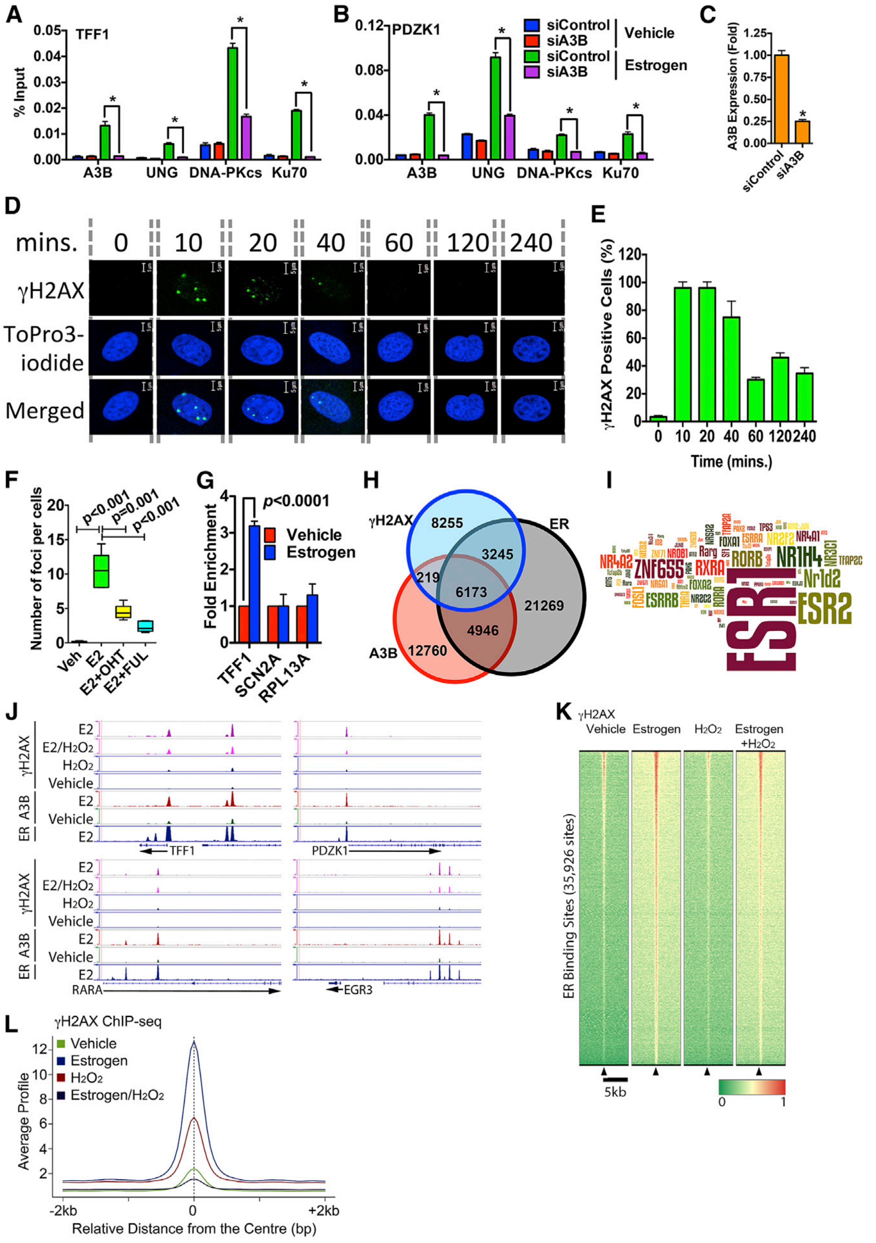

Figure 4. Estrogen Treatment of MCF7 Cells Induces DSBs at ER Binding Regions

Hormone-depleted MCF7 cells were used in all experiments.

(A and B) ChIP for A3B, UNG, DNA-PKcs, and Ku70 was performed following estrogen addition to MCF7 cells transfected with siA3B or siControl. Real-time PCR was performed on recovered DNA, using primers flanking the ER binding regions in TFF1 and PDZK1 genes (n = 3, *p < 0.001).

(C) A3B mRNA levels by real-time PCR for samples used above.

(D) Estrogen was added and cells were immunostained for γH2AX. Nuclei were visualized with the TOPRO DNA stain.

(E) γH2AX in 100 cells from five replicates (total n = 500) was quantified using Cell Profiler 2.0.

(F) OHT or FUL was added for 1 hr, followed by addition of estrogen (E2) for 10 min. Boxplots show the mean γH2AX foci number in 100 cells (n = 5).

(G) γH2AX ChIP (MCF7) and real-time PCR for the TFF1 ER binding site or promoter regions of the SCN2A1 and RPL13A genes (n = 3).

(H) Venn diagram of γH2AX, A3B, and ER binding events from ChIP-seq experiments for estrogen-treated cells. Individual peaks were identified using the same peak-calling algorithm (MACS1.4) using identical settings.

(I) Analysis of the relative enrichment of transcription factor binding sites was used to generate a *Z* score, which is represented by the size of the motif in the word cloud.

(J) Genome browser snapshots of γH2AX ChIP-seq in MCF7 cells treated with estrogen, H_2O_2, or vehicle.

(K) Heatmap showing clustered binding signals for regions enriched in γH2AX for all treatment conditions. The windows represent ±5.0-kb regions from the center of the ER binding events. The color scale shows relative enrichment based on raw signal.

(L) Average signal intensities of γH2AX ChIP-seq binding events centered on ER binding regions are shown for the different treatments. Signal intensity is a normalized count of individual, non-redundant ChIP fragments at single ER binding sites identified with the MACS1.4 peak-calling algorithm.

dU excision by UNG results in the generation of DNA strand breaks that can be repaired by the NHEJ pathway (Kotnis et al., 2009; Nowarski et al., 2012). Moreover, high-level A3G expression in leukemia cells promotes DNA strand breaks (Kotnis et al., 2009; Nowarski et al., 2012). Finally, A3B overexpression promotes γH2AX (Burns et al., 2013a), which is activated at DNA strand breaks (DSB) and is thus a marker for DSB. We reasoned, therefore, that targeted A3B-mediated cytidine deamination and dU excision by UNG could promote DSB generation at ER/A3B enhancers. If so, then estrogen treatment of breast cancer cells should be sufficient to cause DSBs in breast cancer cells. Indeed, treatment of MCF7 cells with estrogen induced γH2AX within 10 min (Figures 4D and 4E). γH2AX induction required ER, since synthetic ER ligands also induced γH2AX (Figures S5A and S5B), while treatment with anti-estrogens 4-hydroxytamoxifen (OHT) or fulvestrant (FUL) prevented estrogen induction of γH2AX (Figures 4F and S5C), as did transfection with ER siRNA (Figure S5D). Estrogen similarly induced γH2AX in an ER-dependent manner in T47D cells (Figures S5E and S5F), but not in the ER- MDA-MB-231 cells (Figure S6A). However, estrogen stimulation of γH2AX was possible in MDA-MB-231 cells ectopically expressing ER.

Estrogen induced γH2AX required DNA-PK and ATM activities and the γH2AX foci co-localized with 53BP1 (Figures S5G and S5H), verifying that estrogen treatment promotes DSBs. We used ChIP for γH2AX to determine if the DSBs localize to ER

and A3B binding regions. Estrogen treatment stimulated γH2AX at the ER/A3B binding region in the TFF1 gene, but no γH2AX enrichment was observed at the promoter of a gene that is not expressed in MCF7 cells (SCN2A) (Ju et al., 2006), nor was γH2AX enrichment observed at the non-ER target gene RPL13A, which is expressed in MCF7 cells (Figure 4G). We undertook γH2AX ChIP-seq to determine the global distribution of γH2AX following estrogen treatment (Figure S7A). Peak calling identified 17,892 γH2AX binding events in the estrogen-treated samples. Using the definition that a binding region must overlap by at least one base pair, 54% (9,637/17,892) of γH2AX regions co-localized with A3B and/or ER binding events, with two-thirds (64%, 6,173/9,637) of these regions co-localizing to A3B and ER co-incident binding events (Figure 4H). Motif enrichment analysis confirmed that the γH2AX regions are highly enriched for ER (ESR1) binding motifs (Figure 4I). Correlation coefficient values for the raw sequencing data confirmed the co-localization of γH2AX regions with A3B (r^2 = 0.69) and ER (r^2 = 0.70) binding events (Figure S7B). Genomic loci exemplifying γH2AX at A3B and ER binding regions are shown in Figure 4J.

Aligning the γH2AX peaks showed very little enrichment for γH2AX in vehicle-treated cells at ER binding regions (Figures 4K and 4L). H_2O_2 treatment, which induced γH2AX, also did not result in much enrichment of γH2AX at ER binding regions. Indeed, co-treatment with H_2O_2 and estrogen reduced γH2AX at ER regions, compared with estrogen alone. Indeed, the majority of γH2AX regions induced by estrogen were enriched for ER and A3B binding (Figure S7C). There was some enrichment for ER and A3B sites in γH2AX regions that were common to all treatments. However, there was very little overlap with ER or A3B sites for γH2AX regions present in vehicle treated, or following H_2O_2 treatment, indicating that estrogen/ER induced γH2AX occurs at sites that are quite distinct from those caused by DNA-damaging agents.

As described above, the greater part of ER and A3B binding occurs at distal regions. Active regulatory regions such as enhancers and promoters carry specific epigenetic modifications including H3K27ac (enhancers and promoters), H3K4me1 (enhancer specific), and H3K4me3 (promoter specific) (ENCODE Project Consortium, 2012). Interestingly, A3B was found at 93% of active enhancers and 7% of active promoters (Figure S7D). This was confirmed by strong enrichment for BRD4 and p300, two ubiquitous co-activators found at active regulatory elements (Hnisz et al., 2013). In addition, using recently published GRO-seq data (Hah et al., 2013), we could identify bi-directional transcription at a subset of distally bound A3B sites, indicating the possibility of eRNA synthesis at these elements.

APOBEC3B Action at ER Target Genes Causes Transient DNA Strand Breaks That Are Repaired by NHEJ

Our results demonstrate that A3B induces C-to-U transitions and promotes UNG, DNA-PK, and Ku70 recruitment to ER binding regions (Figures 4A and 4B). We have also shown that estrogen/ER rapidly induces γH2AX globally at ER and A3B binding regions. These findings imply that A3B action is required for DSB generation at ER binding regions. Indeed, A3B knockdown blocked estrogen-induced γH2AX in MCF7 cells (Figures 5A, 5B, and S5D) and prevented γH2AX at ER binding sites in the TFF1 and PDZK1 genes (Figure 5C). Similarly, UNG was required, as its knockdown also inhibited estrogen induction of γH2AX (Figure S5I). Moreover, estrogen induction of γH2AX in MDA-MB-231 cells expressing ER required A3B (Figures S6B and S6C). A3B was also necessary for optimal expression of ER-regulated genes in the ER-expressing MDA-MB-231 lines (Figure S6D).

Next, we used biotin-16-deoxyuridine triphosphate (dUTP) labeling of DSBs with terminal deoxynucleotide transferase (TdT) (Ju et al., 2006) followed by biotin ChIP and real-time PCR to directly determine if DSBs are formed at ER/A3B binding regions. Real-time PCR using primers located 3′ to the region in the TFF1 gene to which the great majority of C-to-U transitions mapped showed 6-fold enrichment over the vehicle control within 10 min of estrogen addition (Figure 5D). DSBs in this region were reduced to basal levels by 60 min, in general agreement with the reduction in γH2AX over this time frame. A similar rapid but transient induction of DSBs was observed for other ER target genes, but there were no detectable DSBs at the non-expressed SCN2A1 gene promoter, or at a region 2 kb 5′ to the ER binding region in TFF1 (TFF1 control). PCR using primers A/C, which amplify across the region containing the C-to-U transitions failed to show estrogen stimulation of DSBs, indicating that estrogen induces DSBs within the region of the TFF1 gene that is characterized by A3B-mediated C-to-U transitions (Figure 5E). Estrogen induction of DSBs was prevented if the cells were transfected with A3B siRNAs (Figures 5E and 5F).

A3B Is Required for Histone Modification and Recruitment of Chromatin Remodeling Factors at ER Binding Regions

Transcription factors regulate gene expression by promoting the ordered recruitment of diverse complexes that modify and remodel chromatin, leading to transcription initiation. Recent studies show that DNA repair factors regulate gene expression by aiding chromatin remodeling (Fong et al., 2013). A3B knockdown prevented estrogen stimulation of histone modifications associated with transcription at ER target genes (Figures 6A and 6B). Importantly, H2AX phosphorylation at serine-139 (γH2AX) promotes chromatin recruitment of BRG1, the catalytic subunit of the SWI/SNF ATPase-dependent chromatin remodeling complex (Lee et al., 2010). In agreement with a model in which A3B-mediated cytidine deamination leads to H2AX activation, A3B knockdown prevented BRG1 recruitment (Figures 6C and 6D). A3B knockdown also inhibited PolII recruitment to the TFF1 and GREB1 genes. Interestingly, the estrogen-stimulated γH2AX co-localized with activated (phosphorylated) PolII, further evidence that the estrogen/ER and A3B regulated DSB formation occurs of transcriptionally active chromatin. In addition to regulating histone modification and recruitment of chromatin remodelers, A3B was required for PolII recruitment to ER/A3B binding regions (Figures 6E and 6F). The importance of H2AX activation was underscored by the fact that treatment with the DNA-PKcs inhibitor NU7441 or the ATM inhibitor KU55933 inhibited histone modification, as well as BRG1 and PolII recruitment at ER target genes (Figures 6G–6M). Note that these treatments did not affect A3B and ER recruitment.

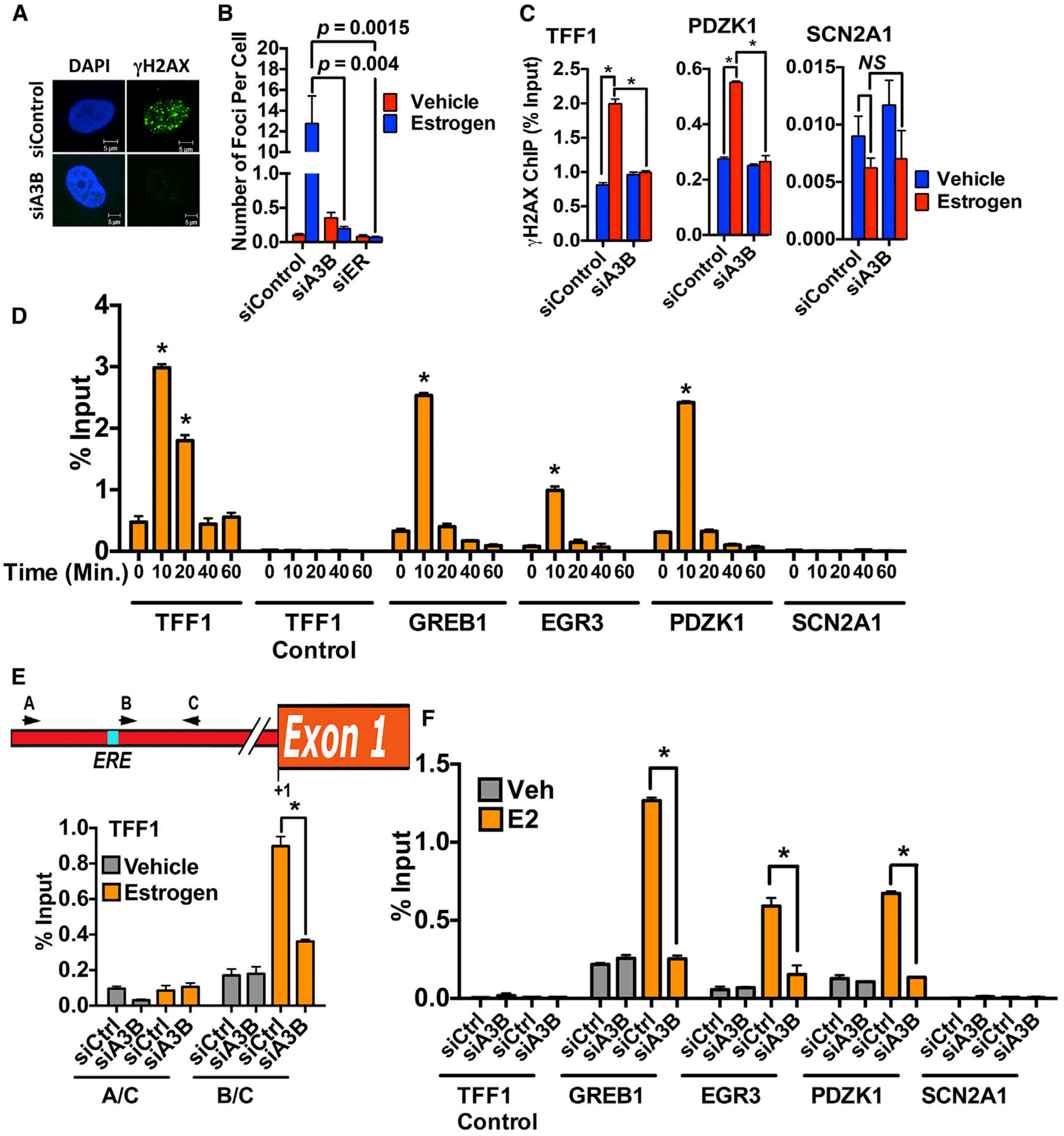

Figure 5. Estrogen-Induced DNA Strand Breaks Are Dependent on A3B

(A) Hormone-depleted MCF7 cells were used for all experiments. Cells transfected with siA3B or siControl were treated with estrogen. Shown are representative images for γH2AX staining.

(B) The average γH2AX foci number per cell in 100 cells from five replicates ± SEM.

(C) ChIP assay with MCF7 cells transfected with A3B or control siRNA.

(D) Estrogen was added and cells end labeled by incubation with biotin-16-dUTP in the presence of terminal deoxynucleotide transferase (TdT). ChIP was performed with a biotin antibody.

(E and F) Estrogen was added following siRNA transfections for 10 min. Biotin end labeling was performed as in (D).

(C–F) n = 3; *p < 0.001; NS, not significant.

DISCUSSION

The molecular mechanisms by which ER drives breast cancer has identified transcription factors that direct ER to active enhancers (Magnani et al., 2011) and revealed that ER controls the coordinated recruitment of chromatin remodeling and modification proteins and the transcription machinery that together facilitate the program of estrogen-responsive gene expression (Métivier et al., 2006). There is also gathering evidence that many protein complexes that sense and repair DNA damage are important for regulation of gene expression by ER and other transcription factors, their recruitment aiding chromatin modification/remodeling to promote gene activation (Fong et al., 2013). For example, the BER protein thymine DNA glycosylase

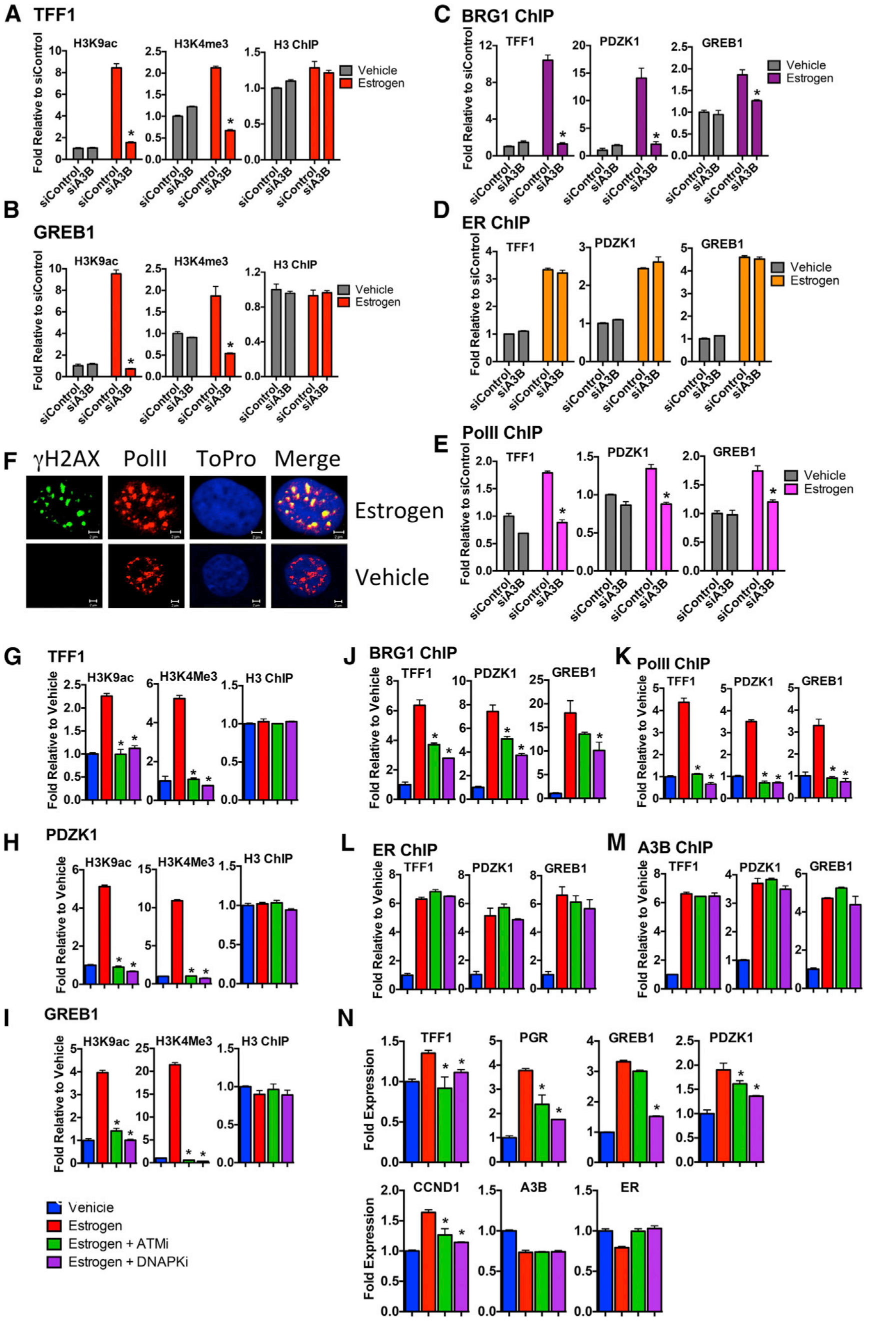

Figure 6. A3B Is Required for Chromatin Remodeling and Activating Histone Modifications at ER Enhancers

All experiments were undertaken with hormone-depleted MCF7 cells.

(A and B) ChIP for histone H3 and H3 modifications associated with gene activation, was performed following estrogen addition to cells transfected with siA3B or control siRNA (n = 3, $^*p < 0.001$).

(C–E) ChIP for BRG1, ER, and PolII followed by real-time for A3B/ER binding sites in TFF1, PDZK1, and GREB1.

(F) Cells were treated with estrogen for 10 min and immunostained for γH2AX (green) and PolII (red).

(G–M) 5 μM NU7441 (DNA-PKcs inhibitor) or KU55933 (ATM inhibitor) was added for 1 hr followed by estrogen addition. ChIP was performed as above (n = 3, $^*p < 0.001$).

(N) Cells were treated with NU7441 or KU55933 for 1 hr, at which point estrogen was added. Real-time RT-PCR was performed with RNA prepared after 4 hr (n = 3; $^*p < 0.001$).

acts as a co-activator for ER and promotes recruitment of the p160 co-activators and the CBP/p300 histone acetyltransferase (Lucey et al., 2005; Tini et al., 2002). One reason advanced for the function of DNA repair proteins in gene regulation is that they may aid in relieving torsional stress generated by transcription-induced DNA supercoiling (Ma and Wang, 2014). Movement of RNA polymerase (RNAP) along the DNA template during transcription generates over-winding (positive DNA supercoiling) in front and negative DNA supercoiling behind it. Failure to resolve DNA supercoiling will ultimately affect transcription. Additionally, generation of DNA supercoiling at one promoter can affect transcription from a distal promoter, so-called topological promoter coupling (Ma and Wang, 2014). Given the recent identification of active transcription at enhancer regions, which generates eRNAs, RNAP procession is also likely to create torsional stress at enhancer regions, which might contribute to inhibition of transcription from coupled gene promoters. Furthermore, regulation of transcription by enhancer regions entails communication between distal enhancers and regulated promoters through chromatin looping, a process that is also influenced by DNA supercoiling (Kulaeva et al., 2012). Thus, DNA topoisomerases, which relax negative and positive DNA supercoils, are important in transcription regulation. Indeed, ER- and androgen receptor (AR)-induced transcription in breast and prostate cancer involves transient DSBs generated by TOP1 and TOP2 (Ju et al., 2006; Puc et al., 2015). Activation of the LSD1 histone demethylase by ER binding promoted 8-oxoguanine modification of DNA and recruitment of 8-oxoguanine-DNA glycosylase and BER to stimulate chromatin changes that facilitate interaction between ER-regulated gene enhancers and promoters (Perillo et al., 2008). Thus, DSB formation and their resolution is an important component of the regulation of gene expression by of ER.

Here, we report an alternative mechanism for transcription of ER-regulated genes in which DSBs are generated in a process initiated by estrogen-ER-dependent recruitment of A3B. The importance of A3B in ER action is implied by our observation of the exceptionally high genome-wide co-localization of ER

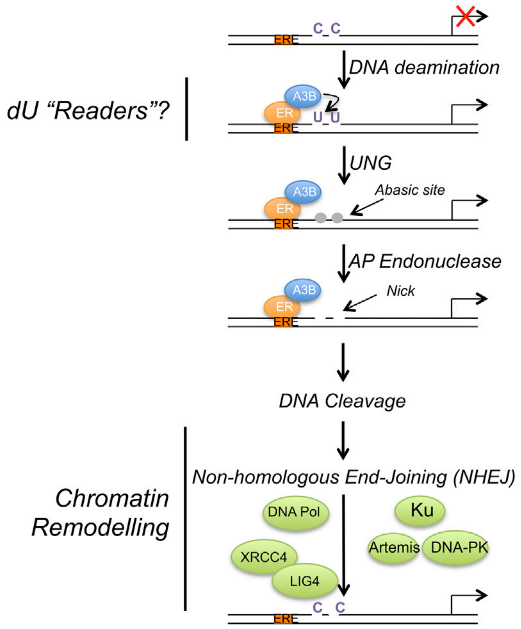

Figure 7. Model for A3B-Mediated Activation of ER Enhancers toward Regulation of ER Target Gene Expression

Estrogen binding to ER promotes its recruitment to ER enhancers. A3B, recruited to these regions through interaction with ER, provides enzymatic conversion of C to U. Generation of U:G mismatches promotes DNA nicks through the action of UNG and AP endonuclease, resulting in DNA cleavage and repair by the non-homologous end-joining DNA repair pathway. Induction of transient C-to-U changes stimulates chromatin modification and remodeling and PolII recruitment to facilitate expression of ER target genes.

and A3B binding regions. Moreover, we show that estrogen treatment causes rapid induction of DSBs, as demonstrated by γH2AX activation. Importantly, the majority of estrogen-induced γH2AX occurs at ER and A3B binding regions, and we have shown that γH2AX induction is dependent on A3B. Our work demonstrates that A3B directs cytidine deamination at ER binding regions to facilitate DSBs through activation of BER and subsequent repair of these lesions by the NHEJ pathway. The critical role of A3B action in the regulation of gene expression by ER is established by its requirement for breast cancer cell growth in vitro and in vivo. Based on these findings, we propose that the A3B-mediated generation of C-to-U changes and activation of DNA repair pathways facilitates chromatin remodeling and enhancer/promoter interaction. In support of this, A3B is required for SWI/SNF recruitment, activating histone modifications and PolII recruitment to ER binding regions (a schematic model is shown in Figure 7).

Interestingly, DNA damage by irradiation or with the use of models in which DNA DSBs are locally induced with restriction enzymes such as I-SceI has demonstrated that ATM promotes dynamic chromatin condensation and transcriptional silencing at DSBs (Khurana et al., 2014; Shanbhag et al., 2010). DNA-PKcs can repress transcription (Pankotai et al., 2012), but its recruitment to transcription-factor-promoted DSBs stimulates transcription (Ju et al., 2006). As proposed by Tjian and colleagues (Fong et al., 2013), transient DSB generation by transcription factors may limit γH2AX accumulation and spreading to check retention of DNA repair factors and thus control the extent of the DDR response. In agreement with this model, DNA DSB-induced H2AX phosphorylation is spread over large domains around the DSB (Iacovoni et al., 2010). This contrasts with the restricted γH2AX distribution induced by A3B recruitment to ER binding regions observed here. Thus, the mode of ATM and DNA-PKcs recruitment appears to determine the effect of DSBs on transcription.

Proteins that preferentially bind to DNA sequences containing methylcytosine (mC) to regulate chromatin and control gene expression are well described. Importantly, proteins that bind to other CpG modifications, including 5-hydroxymethlycytosine (hmC), 5-formylcytosine (fC), and 5-carboxylcytosine (caC) are now being identified and include not only DNA repair factors but also chromatin regulators and transcription factors (Iurlaro et al., 2013; Spruijt et al., 2013). Our findings allow that there may be transcription regulatory proteins that interact with DNA containing dU and that are therefore recruited upon A3B-mediated cytidine deamination at gene enhancers, an intriguing possibility that may deserve further investigation.

Recent studies show that cytidine deamination is an important feature of the mutational landscape in breast (Alexandrov et al., 2013; Burns et al., 2013a; Nik-Zainal et al., 2012), ovarian (Leonard et al., 2013), lung (de Bruin et al., 2014), and other cancers (Burns et al., 2013b; Roberts et al., 2013), with high-level expression of A3B, and experimental studies indicate that A3B may be a key driver of such mutational signatures (Burns et al., 2013a; Taylor et al., 2013). It is tempting to speculate that the cytidine-deaminase-associated mutational landscapes in breast cancer might be enriched at ER/A3B binding regions. In support of this possibility, AR was shown to promote DNA DSBs to aid intra- and inter-chromosomal translocations in prostate cancer cells, one mechanism for which involved the hormone-dependent recruitment of AID (Lin et al., 2009). Determination of the global ER binding profiles by ChIP-seq analysis of breast tumors has shown that there is a high level of plasticity in ER binding in breast cancer (Ross-Innes et al., 2012), such that investigation of any association between A3B/ER binding regions and somatic mutations may entail whole-genome sequencing, coupled with ER and A3B ChIP-seq in the same tumor. Interestingly, however, gene regulatory regions can be characterized by low levels of somatic mutations in cancer in the absence of additional defects in DNA repair (Polak et al., 2014). Our results suggest that activation of DNA repair pathways may protect enhancer regions from A3B-dependent mutagenesis. It would be important therefore to investigate the involvement of defects in BER and/or NHEJ in cancer mutational landscapes that have been ascribed to A3B.

In summary, our results identify an important role for A3B as a regulator of ER-mediated gene expression in breast cancer, with potential as a therapeutic target in ER+ breast cancer. To advance this possibility, it will be important to extend our findings to studies that identify global A3B regulated genes in breast cancer cell lines, as well as in tumor samples. Moreover, as A3B is widely expressed in other cancers, it is likely that A3B inhibition represents an important therapeutic approach to inhibit regulated transcription in other cancer types.

EXPERIMENTAL PROCEDURES

Cell Lines, Plasmids, Antibodies, and Real-Time RT-PCR Assays

Cell lines were obtained from and cultured in media recommended by ATCC. MCF7, T47D, SkBr3, COS-1 and HeLa cells were grown in DMEM containing 10% fetal calf serum (FCS). MDA-MB-231 cells stably expressing ER have been described (Bhat-Nakshatri et al., 2004). Hormone depletion was achieved by culturing cells for 72 hr in DMEM lacking phenol red and containing 5% dextran-coated charcoal-stripped FCS. Plasmids, antibodies, and real-time PCR primers are detailed in the Supplemental Experimental Procedures.

Reporter Gene Assays

Hormone-depleted COS-1 cells were transfected with ERE3-TATA-luc, pRL-TK, together with ER and hemagglutinin (HA)-tagged A3B. Estrogen (10 nM) or an equal volume of ethanol (vehicle) was added 5 hr following transfection. Transfection methodology and luciferase measurements were performed as described previously (Lai et al., 2013). Reporter gene assays in HeLa cells following transfection with ER, A3B, and myc-UGI-NLS were undertaken as above.

siRNA

Cells were transfected with siRNA using Lipofectamine RNAiMax (Invitrogen). For collecting RNA and protein, 10 nM estrogen was added after 48 hr; RNA and protein lysates were prepared after a further 12 hr. Cell growth was determined using the sulforhodamine B (SRB) assay, as described previously (Lai et al., 2013). Details of siRNAs are provided in the Supplemental Experimental Procedures.

MCF7 Human Tumor Xenografts

10 μM siRNA prepared with the atelogene in vivo siRNA transfection kit (Koken, Japan) was injected weekly directly into tumors. Tumor volumes were determined twice weekly. At the end of the experiment, protein lysates were prepared from half of each tumor by homogenization in RIPA buffer. RNA was prepared from the remaining halves of each tumor using the RNAeasy kit (QIAGEN). The study was undertaken under the auspices of a UK Home Office project license, using approved procedures.

Gene Expression

Total RNA was prepared and real-time RT-PCR was performed as described previously (Ngan et al., 2009), using TaqMan gene expression assays from ABI.

Immunoprecipitations

Immunoprecipitations were performed as described previously (Lopez-Garcia et al., 2006).

Immunofluorescence

Cells were cultured on glass coverslips in phenol red-free DMEM containing 5% double charcoal-stripped FCS for 3 days before the addition of ligands. Cells were fixed and incubated with antibodies, as described in the Supplemental Experimental Procedures. Images were acquired using a Zeiss LSM510 confocal microscope. Images were analyzed using Fuji Image J (NIH) and CellProfiler (Broad Institute) for quantification of staining.

ChIP

ChIP was performed as described previously (Lai et al., 2013), using 10 μg of antibody and 100 μl of Protein A Dynalbeads (10002D; Invitrogen). Control ChIP was performed by the addition of mouse immunoglobulins (IgG).

ChIP and Solexa Sequencing

ChIP DNA was amplified as described (Schmidt et al., 2009). Sequences were generated by the Illumina Hiseq 2000 genome analyzer (using 50 bp reads) and aligned to the Human Reference Genome (assembly hg19, February 2009) using Bowtie 1.0. The model-based analysis for ChIP-seq (MACS) peak caller version 1.4 (Zhang et al., 2008) was used to identify enriched regions of the genome by comparison to an input sample. MACS was used in the default setting with a p value threshold of 10^{-5}. To call stimuli specific peaks, we used bedtool to subtract or concatenate BED files generated by MACS. The number of reads obtained, percentage of reads aligned, peaks called, and additional analysis methods are detailed in the Supplemental Experimental Procedures.

Biotin Labeling of DSB

Biotin labeling was performed as described previously (Ju et al., 2006) and detailed in the Supplemental Experimental Procedures.

3D-PCR, Cloning, and Sequencing

3D-PCR was carried out as described elsewhere (Suspène et al., 2005). Genomic DNA was prepared using the Invitrogen genomic extraction kit. Products from the second round PCR were purified using the QIAGEN PCR purification kit, cloned into the TOPO-TA cloning vector (Invitrogen), and sequenced with the T7 sequencing primer.

ACCESSION NUMBERS

The accession numbers for the ChIP-seq data reported in this paper are GEO: GSE56979 (A3B ChIP-seq) and GEO: GSE57426 (γH2AX ChIP-seq).

SUPPLEMENTAL INFORMATION

Supplemental information includes Supplemental Experimental Procedures, seven figures, and one table and can be found with this article online at http://dx.doi.org/10.1016/j.celrep.2015.08.066.

ACKNOWLEDGMENTS

We are grateful to M.-A. Langlois and the late M. Neuberger for helpful discussions and plasmids. We thank B. Cullen and H. Weingard for APOBEC plasmids and J. Cohen for UGI. We also thank A. Ashworth and P. Edwards for breast cancer cell lines and H. Nakshatri for MDA-MB-231-ER cells. For their help, we thank R. Kerkhoven and L. Game (Solexa sequencing) and K.I. Hng (microscopy). This work was funded by CRUK and Breast Cancer Now. N.N. is funded by the MRC.

Received: May 14, 2015
Revised: July 16, 2015
Accepted: August 24, 2015
Published: September 24, 2015

REFERENCES

Alexandrov, L.B., Nik-Zainal, S., Wedge, D.C., Aparicio, S.A., Behjati, S., Biankin, A.V., Bignell, G.R., Bolli, N., Borg, A., Børresen-Dale, A.L., et al.; Australian Pancreatic Cancer Genome Initiative; ICGC Breast Cancer Consortium; ICGC MMML-Seq Consortium; ICGC PedBrain (2013). Signatures of mutational processes in human cancer. Nature *500*, 415–421.

Ali, S., and Coombes, R.C. (2002). Endocrine-responsive breast cancer and strategies for combating resistance. Nat. Rev. Cancer *2*, 101–112.

Anzick, S.L., Kononen, J., Walker, R.L., Azorsa, D.O., Tanner, M.M., Guan, X.Y., Sauter, G., Kallioniemi, O.P., Trent, J.M., and Meltzer, P.S. (1997). AIB1, a steroid receptor coactivator amplified in breast and ovarian cancer. Science *277*, 965–968.

Bhat-Nakshatri, P., Campbell, R.A., Patel, N.M., Newton, T.R., King, A.J., Marshall, M.S., Ali, S., and Nakshatri, H. (2004). Tumour necrosis factor and PI3-kinase control oestrogen receptor alpha protein level and its transrepression function. Br. J. Cancer *90*, 853–859.

Bhutani, N., Burns, D.M., and Blau, H.M. (2011). DNA demethylation dynamics. Cell *146*, 866–872.

Bogerd, H.P., Wiegand, H.L., Doehle, B.P., and Cullen, B.R. (2007). The intrinsic antiretroviral factor APOBEC3B contains two enzymatically active cytidine deaminase domains. Virology *364*, 486–493.

Burns, M.B., Lackey, L., Carpenter, M.A., Rathore, A., Land, A.M., Leonard, B., Refsland, E.W., Kotandeniya, D., Tretyakova, N., Nikas, J.B., et al. (2013a).

APOBEC3B is an enzymatic source of mutation in breast cancer. Nature *494*, 366–370.

Burns, M.B., Temiz, N.A., and Harris, R.S. (2013b). Evidence for APOBEC3B mutagenesis in multiple human cancers. Nat. Genet. *45*, 977–983.

Compe, E., and Egly, J.M. (2012). TFIIH: when transcription met DNA repair. Nat. Rev. Mol. Cell Biol. *13*, 343–354.

Conticello, S.G. (2008). The AID/APOBEC family of nucleic acid mutators. Genome Biol. *9*, 229.

Curtis, C., Shah, S.P., Chin, S.F., Turashvili, G., Rueda, O.M., Dunning, M.J., Speed, D., Lynch, A.G., Samarajiwa, S., Yuan, Y., et al.; METABRIC Group (2012). The genomic and transcriptomic architecture of 2,000 breast tumours reveals novel subgroups. Nature *486*, 346–352.

Dahlman-Wright, K., Cavailles, V., Fuqua, S.A., Jordan, V.C., Katzenellenbogen, J.A., Korach, K.S., Maggi, A., Muramatsu, M., Parker, M.G., and Gustafsson, J.A. (2006). International Union of Pharmacology. LXIV. Estrogen receptors. Pharmacol. Rev. *58*, 773–781.

de Bruin, E.C., McGranahan, N., Mitter, R., Salm, M., Wedge, D.C., Yates, L., Jamal-Hanjani, M., Shafi, S., Murugaesu, N., Rowan, A.J., et al. (2014). Spatial and temporal diversity in genomic instability processes defines lung cancer evolution. Science *346*, 251–256.

Desmedt, C., Piette, F., Loi, S., Wang, Y., Lallemand, F., Haibe-Kains, B., Viale, G., Delorenzi, M., Zhang, Y., d'Assignies, M.S., et al.; TRANSBIG Consortium (2007). Strong time dependence of the 76-gene prognostic signature for node-negative breast cancer patients in the TRANSBIG multicenter independent validation series. Clin. Cancer Res. *13*, 3207–3214.

ENCODE Project Consortium (2012). An integrated encyclopedia of DNA elements in the human genome. Nature *489*, 57–74.

Fong, Y.W., Cattoglio, C., and Tjian, R. (2013). The intertwined roles of transcription and repair proteins. Mol. Cell *52*, 291–302.

Guedj, M., Marisa, L., de Reynies, A., Orsetti, B., Schiappa, R., Bibeau, F., MacGrogan, G., Lerebours, F., Finetti, P., Longy, M., et al. (2012). A refined molecular taxonomy of breast cancer. Oncogene *31*, 1196–1206.

Hah, N., Murakami, S., Nagari, A., Danko, C.G., and Kraus, W.L. (2013). Enhancer transcripts mark active estrogen receptor binding sites. Genome Res. *23*, 1210–1223.

Hnisz, D., Abraham, B.J., Lee, T.I., Lau, A., Saint-André, V., Sigova, A.A., Hoke, H.A., and Young, R.A. (2013). Super-enhancers in the control of cell identity and disease. Cell *155*, 934–947.

Iacovoni, J.S., Caron, P., Lassadi, I., Nicolas, E., Massip, L., Trouche, D., and Legube, G. (2010). High-resolution profiling of gammaH2AX around DNA double strand breaks in the mammalian genome. EMBO J. *29*, 1446–1457.

Iurlaro, M., Ficz, G., Oxley, D., Raiber, E.A., Bachman, M., Booth, M.J., Andrews, S., Balasubramanian, S., and Reik, W. (2013). A screen for hydroxymethylcytosine and formylcytosine binding proteins suggests functions in transcription and chromatin regulation. Genome Biol. *14*, R119.

Ju, B.G., Lunyak, V.V., Perissi, V., Garcia-Bassets, I., Rose, D.W., Glass, C.K., and Rosenfeld, M.G. (2006). A topoisomerase IIbeta-mediated dsDNA break required for regulated transcription. Science *312*, 1798–1802.

Kamileri, I., Karakasilioti, I., and Garinis, G.A. (2012). Nucleotide excision repair: new tricks with old bricks. Trends Genet. *28*, 566–573.

Khurana, S., Kruhlak, M.J., Kim, J., Tran, A.D., Liu, J., Nyswaner, K., Shi, L., Jailwala, P., Sung, M.H., Hakim, O., and Oberdoerffer, P. (2014). A macrohistone variant links dynamic chromatin compaction to BRCA1-dependent genome maintenance. Cell Rep. *8*, 1049–1062.

Komatsu, A., Nagasaki, K., Fujimori, M., Amano, J., and Miki, Y. (2008). Identification of novel deletion polymorphisms in breast cancer. Int. J. Oncol. *33*, 261–270.

Kotnis, A., Du, L., Liu, C., Popov, S.W., and Pan-Hammarström, Q. (2009). Non-homologous end joining in class switch recombination: the beginning of the end. Philos. Trans. R. Soc. Lond. B Biol. Sci. *364*, 653–665.

Kulaeva, O.I., Nizovtseva, E.V., Polikanov, Y.S., Ulianov, S.V., and Studitsky, V.M. (2012). Distant activation of transcription: mechanisms of enhancer action. Mol. Cell. Biol. *32*, 4892–4897.

Lai, C.F., Flach, K.D., Alexi, X., Fox, S.P., Ottaviani, S., Thiruchelvam, P.T., Kyle, F.J., Thomas, R.S., Launchbury, R., Hua, H., et al. (2013). Co-regulated gene expression by oestrogen receptor α and liver receptor homolog-1 is a feature of the oestrogen response in breast cancer cells. Nucleic Acids Res. *41*, 10228–10240.

Landry, S., Narvaiza, I., Linfesty, D.C., and Weitzman, M.D. (2011). APOBEC3A can activate the DNA damage response and cause cell-cycle arrest. EMBO Rep. *12*, 444–450.

Lee, H.S., Park, J.H., Kim, S.J., Kwon, S.J., and Kwon, J. (2010). A cooperative activation loop among SWI/SNF, gamma-H2AX and H3 acetylation for DNA double-strand break repair. EMBO J. *29*, 1434–1445.

Leonard, B., Hart, S.N., Burns, M.B., Carpenter, M.A., Temiz, N.A., Rathore, A., Vogel, R.I., Nikas, J.B., Law, E.K., Brown, W.L., et al. (2013). APOBEC3B upregulation and genomic mutation patterns in serous ovarian carcinoma. Cancer Res. *73*, 7222–7231.

Lin, C., Yang, L., Tanasa, B., Hutt, K., Ju, B.G., Ohgi, K., Zhang, J., Rose, D.W., Fu, X.D., Glass, C.K., and Rosenfeld, M.G. (2009). Nuclear receptor-induced chromosomal proximity and DNA breaks underlie specific translocations in cancer. Cell *139*, 1069–1083.

Lopez-Garcia, J., Periyasamy, M., Thomas, R.S., Christian, M., Leao, M., Jat, P., Kindle, K.B., Heery, D.M., Parker, M.G., Buluwela, L., et al. (2006). ZNF366 is an estrogen receptor corepressor that acts through CtBP and histone deacetylases. Nucleic Acids Res. *34*, 6126–6136.

Lucey, M.J., Chen, D., Lopez-Garcia, J., Hart, S.M., Phoenix, F., Al-Jehani, R., Alao, J.P., White, R., Kindle, K.B., Losson, R., et al. (2005). T:G mismatch-specific thymine-DNA glycosylase (TDG) as a coregulator of transcription interacts with SRC1 family members through a novel tyrosine repeat motif. Nucleic Acids Res. *33*, 6393–6404.

Ma, J., and Wang, M. (2014). Interplay between DNA supercoiling and transcription elongation. Transcription *5*, e28636.

Magnani, L., Eeckhoute, J., and Lupien, M. (2011). Pioneer factors: directing transcriptional regulators within the chromatin environment. Trends Genet. *27*, 465–474.

McClelland, R.A., Gee, J.M., Francis, A.B., Robertson, J.F., Blamey, R.W., Wakeling, A.E., and Nicholson, R.I. (1996). Short-term effects of pure anti-oestrogen ICI 182780 treatment on oestrogen receptor, epidermal growth factor receptor and transforming growth factor-alpha protein expression in human breast cancer. Eur. J. Cancer *32A*, 413–416.

Métivier, R., Reid, G., and Gannon, F. (2006). Transcription in four dimensions: nuclear receptor-directed initiation of gene expression. EMBO Rep. *7*, 161–167.

Miller, L.D., Smeds, J., George, J., Vega, V.B., Vergara, L., Ploner, A., Pawitan, Y., Hall, P., Klaar, S., Liu, E.T., and Bergh, J. (2005). An expression signature for p53 status in human breast cancer predicts mutation status, transcriptional effects, and patient survival. Proc. Natl. Acad. Sci. USA *102*, 13550–13555.

Nabel, C.S., and Kohli, R.M. (2011). Molecular biology. Demystifying DNA demethylation. Science *333*, 1229–1230.

Ngan, S., Stronach, E.A., Photiou, A., Waxman, J., Ali, S., and Buluwela, L. (2009). Microarray coupled to quantitative RT-PCR analysis of androgen-regulated genes in human LNCaP prostate cancer cells. Oncogene *28*, 2051–2063.

Nik-Zainal, S., Alexandrov, L.B., Wedge, D.C., Van Loo, P., Greenman, C.D., Raine, K., Jones, D., Hinton, J., Marshall, J., Stebbings, L.A., et al.; Breast Cancer Working Group of the International Cancer Genome Consortium (2012). Mutational processes molding the genomes of 21 breast cancers. Cell *149*, 979–993.

Nik-Zainal, S., Wedge, D.C., Alexandrov, L.B., Petljak, M., Butler, A.P., Bolli, N., Davies, H.R., Knappskog, S., Martin, S., Papaemmanuil, E., et al. (2014). Association of a germline copy number polymorphism of APOBEC3A and APOBEC3B with burden of putative APOBEC-dependent mutations in breast cancer. Nat. Genet. *46*, 487–491.

Nowarski, R., Wilner, O.I., Cheshin, O., Shahar, O.D., Kenig, E., Baraz, L., Britan-Rosich, E., Nagler, A., Harris, R.S., Goldberg, M., et al. (2012). APOBEC3G enhances lymphoma cell radioresistance by promoting cytidine deaminase-dependent DNA repair. Blood *120*, 366–375.

O'Donnell, A.J., Macleod, K.G., Burns, D.J., Smyth, J.F., and Langdon, S.P. (2005). Estrogen receptor-alpha mediates gene expression changes and growth response in ovarian cancer cells exposed to estrogen. Endocr. Relat. Cancer *12*, 851–866.

Osborne, C.K., and Schiff, R. (2011). Mechanisms of endocrine resistance in breast cancer. Annu. Rev. Med. *62*, 233–247.

Pankotai, T., Bonhomme, C., Chen, D., and Soutoglou, E. (2012). DNAPKcs-dependent arrest of RNA polymerase II transcription in the presence of DNA breaks. Nat. Struct. Mol. Biol. *19*, 276–282.

Perillo, B., Ombra, M.N., Bertoni, A., Cuozzo, C., Sacchetti, S., Sasso, A., Chiariotti, L., Malorni, A., Abbondanza, C., and Avvedimento, E.V. (2008). DNA oxidation as triggered by H3K9me2 demethylation drives estrogen-induced gene expression. Science *319*, 202–206.

Polak, P., Lawrence, M.S., Haugen, E., Stoletzki, N., Stojanov, P., Thurman, R.E., Garraway, L.A., Mirkin, S., Getz, G., Stamatoyannopoulos, J.A., and Sunyaev, S.R. (2014). Reduced local mutation density in regulatory DNA of cancer genomes is linked to DNA repair. Nat. Biotechnol. *32*, 71–75.

Puc, J., Kozbial, P., Li, W., Tan, Y., Liu, Z., Suter, T., Ohgi, K.A., Zhang, J., Aggarwal, A.K., and Rosenfeld, M.G. (2015). Ligand-dependent enhancer activation regulated by topoisomerase-I activity. Cell *160*, 367–380.

Roberts, S.A., Lawrence, M.S., Klimczak, L.J., Grimm, S.A., Fargo, D., Stojanov, P., Kiezun, A., Kryukov, G.V., Carter, S.L., Saksena, G., et al. (2013). An APOBEC cytidine deaminase mutagenesis pattern is widespread in human cancers. Nat. Genet. *45*, 970–976.

Ross-Innes, C.S., Stark, R., Teschendorff, A.E., Holmes, K.A., Ali, H.R., Dunning, M.J., Brown, G.D., Gojis, O., Ellis, I.O., Green, A.R., et al. (2012). Differential oestrogen receptor binding is associated with clinical outcome in breast cancer. Nature *481*, 389–393.

Sabatier, R., Finetti, P., Cervera, N., Lambaudie, E., Esterni, B., Mamessier, E., Tallet, A., Chabannon, C., Extra, J.M., Jacquemier, J., et al. (2011). A gene expression signature identifies two prognostic subgroups of basal breast cancer. Breast Cancer Res. Treat. *126*, 407–420.

Schmidt, D., Wilson, M.D., Spyrou, C., Brown, G.D., Hadfield, J., and Odom, D.T. (2009). ChIP-seq: using high-throughput sequencing to discover protein-DNA interactions. Methods *48*, 240–248.

Shanbhag, N.M., Rafalska-Metcalf, I.U., Balane-Bolivar, C., Janicki, S.M., and Greenberg, R.A. (2010). ATM-dependent chromatin changes silence transcription in cis to DNA double-strand breaks. Cell *141*, 970–981.

Shang, Y. (2006). Molecular mechanisms of oestrogen and SERMs in endometrial carcinogenesis. Nat. Rev. Cancer *6*, 360–368.

Spruijt, C.G., Gnerlich, F., Smits, A.H., Pfaffeneder, T., Jansen, P.W., Bauer, C., Münzel, M., Wagner, M., Müller, M., Khan, F., et al. (2013). Dynamic readers for 5-(hydroxy)methylcytosine and its oxidized derivatives. Cell *152*, 1146–1159.

Stavnezer, J. (2011). Complex regulation and function of activation-induced cytidine deaminase. Trends Immunol. *32*, 194–201.

Suspène, R., Henry, M., Guillot, S., Wain-Hobson, S., and Vartanian, J.P. (2005). Recovery of APOBEC3-edited human immunodeficiency virus G->A hypermutants by differential DNA denaturation PCR. J. Gen. Virol. *86*, 125–129.

Taylor, B.J., Nik-Zainal, S., Wu, Y.L., Stebbings, L.A., Raine, K., Campbell, P.J., Rada, C., Stratton, M.R., and Neuberger, M.S. (2013). DNA deaminases induce break-associated mutation showers with implication of APOBEC3B and 3A in breast cancer kataegis. eLife *2*, e00534.

Tini, M., Benecke, A., Um, S.J., Torchia, J., Evans, R.M., and Chambon, P. (2002). Association of CBP/p300 acetylase and thymine DNA glycosylase links DNA repair and transcription. Mol. Cell *9*, 265–277.

Wang, Y., Klijn, J.G., Zhang, Y., Sieuwerts, A.M., Look, M.P., Yang, F., Talantov, D., Timmermans, M., Meijer-van Gelder, M.E., Yu, J., et al. (2005). Gene-expression profiles to predict distant metastasis of lymph-node-negative primary breast cancer. Lancet *365*, 671–679.

Welboren, W.J., van Driel, M.A., Janssen-Megens, E.M., van Heeringen, S.J., Sweep, F.C., Span, P.N., and Stunnenberg, H.G. (2009). ChIP-Seq of ERalpha and RNA polymerase II defines genes differentially responding to ligands. EMBO J. *28*, 1418–1428.

Zhang, Y., Liu, T., Meyer, C.A., Eeckhoute, J., Johnson, D.S., Bernstein, B.E., Nusbaum, C., Myers, R.M., Brown, M., Li, W., and Liu, X.S. (2008). Model-based analysis of ChIP-Seq (MACS). Genome Biol. *9*, R137.

Zhang, Y., Sieuwerts, A.M., McGreevy, M., Casey, G., Cufer, T., Paradiso, A., Harbeck, N., Span, P.N., Hicks, D.G., Crowe, J., et al. (2009). The 76-gene signature defines high-risk patients that benefit from adjuvant tamoxifen therapy. Breast Cancer Res. Treat. *116*, 303–309.

Cell Reports

Supplemental Information

APOBEC3B-Mediated Cytidine Deamination Is Required for Estrogen Receptor Action in Breast Cancer

Manikandan Periyasamy, Hetal Patel, Chun-Fui Lai, Van T.M. Nguyen, Ekaterina Nevedomskaya, Alison Harrod, Roslin Russell, Judit Remenyi, Anna Maria Ochocka, Ross S. Thomas, Frances Fuller-Pace, Balázs Győrffy, Carlos Caldas, Naveenan Navaratnam, Jason S. Carroll, Wilbert Zwart, R. Charles Coombes, Luca Magnani, Laki Buluwela, and Simak Ali

Supplemental Experimental Procedures

Cell lines. All cell lines were tested on a three-monthly basis to ensure they were free from mycoplasma infection and were genotyped for authenticity using the STR profiling cell line authentication service from LGC Standards (UK).

Plasmids. The expression plasmid pSG5, pSG5 encoding ER and the estrogen-responsive firefly luciferase reporter gene (ERE3-TATA-luc) have been described previously (Lopez-Garcia et al., 2006). The Renilla luciferase reporter gene (pRL-TK) was purchased from Promega, UK. HA-tagged human A3B was kindly provided by Prof B. Cullen and has previously been described (Bogerd et al., 2007). Mutagenesis of HA-A3B was performed by site-directed mutagenesis (Stratagene, UK). Mutagenesis primers had the sequences 5'-GTATCCTGGACCCCCgcCCCGGACgcTGTGGCGAAGCTGGCC-3' (A3B-C97A/C100A) and 5'-CATCTCCTGGAGCCCCgcCTTCTCCTGGGGCgccGCCGGGGAAGTG-3' (A3B-C284A/C289A), where the bases shown in lower case are altered from the wild-type sequence, for amino acid substitutions. Drs B Cullen, H. Weingard, M-A Langlois and M Neuberger kindly provided the other APOBEC expression plasmids. Bacteriophage PBS2 UGI, cloned in frame with a nuclear localisation signal (Reddy et al., 1998), kindly provided by Prof J Cohen, was cloned into pCMV6-Entry mammalian expression plasmid, to generate myc/FLAG-tagged UGI-NLS.

siRNA studies. Cells were cultured in DMEM lacking phenol red and containing 5% DSS for three days prior to transfection with siRNA using the Lipofectamine RNAiMax transfection reagent (Invitrogen), according to manufacturer's protocols. Transfections were performed in 6-well plates for RNA and protein preparation, or in individual wells of 96-well plates for growth studies. For collecting RNA and protein, 10 nM estrogen was added after 48 hours. Cells were harvested for RNA and protein after a further 12 hours. For growth assays, estrogen was added 24 hours after transfection. Cell growth

was determined using the Sulphorhodamine B (SRB) growth assay, as described (Thiruchelvam et al., 2011). siRNA from Qiagen was used for ER (SI03114979, 5'-UCCGAGUAUGAUCCUACCAGA-3') and non-targeting control (siControl; AM4635, Ambion). A3B siRNAs had the sequences, 5'-CCUGAUGGAUCCAGACACA-3' (siA3B#1) and 5'-GGUGUAUUUCAAGCCUCAGTT-3' (siA3B#2). siA3B (3'UTR) had the sequence 5'-AAGUGAUUAAUUGGCUCCAUA-3' (Qiagen cat. No: S104180708). UNG knockdown was carried out using Dharmacon pool cat. No. L011795-00.

Immunoblotting. Cell lysates were prepared and Western blotting was performed as described previously (Lai et al., 2013). Antibodies for ER (HC20; sc-543), TFF1 (sc-28925), BCL2 (sc-492), PGR (sc-538), lamin A/C (sc-7292) and HA tag (sc-7392) were purchased from Santa Cruz. Antibodies for cathepsin D (CTD; ab6313), hsp90 (ab13492), and ß-actin (ab6276) were purchased from Abcam, UK. ER (NCL-ER-6F11) and FLAG (F1804) antibodies were from Novocastra and Sigma-Aldrich, respectively. Commercially available antibodies for AIB1 (611105, BD Biosciences), PDZK1 (10507-2-A, Proteintech) and myc (05-724, Millipore) were used. Immunoblotting for A3B was carried out using a rabbit polyclonal antiserum prepared by immunizing with recombinant protein.

Real-time RT-PCR. Total RNA was prepared and real-time RT-PCR was performed as described previously (Lai et al., 2013), using gene expression assays from Applied Biosystems, tabulated below.

Gene	***Assay Number***
AICDA	Hs00757808
APOBEC1	Hs00242340
APOBEC2	Hs00199012
APOBEC4	Hs00378929
APOBEC3A	Hs00377444
APOBEC3B	Hs00358981
APOBEC3C	Hs0028074

APOBEC3D	Hs00537163
APOBEC3F	Hs01665324
APOBEC3G	Hs00222415
APOBEC3H	Hs00419665
BCL2	Hs00411268
Cathepsin D (CTD)	Hs00157201
CCND1	Hs00233498
ESR1	Hs00174860
GAPDH	Hs99999905
GREB1	Hs00536409
PDZK1	Hs00275727
PGR	Hs00172183
TFF1	Hs00170216
UNG	Hs00422172

Co-immunoprecipitations. Immunoprecipitations were carried out as described previously (Lopez-Garcia et al., 2006). Briefly, cells were lysed in RIPA buffer (150 mM NaCl, 1% NP-40, 0.5% deoxycholic acid, 0.1% SDS and 50 mM Tris-HCl (pH 7.5) containing protease inhibitors. Lysates were pre-cleared by incubating with agarose beads (Santa Cruz) at 4°C for 2 hours. Antibodies were incubated with agarose beads for 2 hours at 4°C. Following washing three times with ice-cold PBS, the beads were resuspended in PBS and transferred to the pre-cleared lysates for overnight incubation. Following incubation with protein lysates, the beads were washed in ice-cold PBS and re-suspended in 2X sample buffer (0.125 M Tris-HCl at pH 6.8, 4% SDS, 20% Glycerol, 10% β-mercaptoethanol and 0.004% bromophenol blue), heated at 95°C for 10 minutes and immunoblotted.

Immunofluorescence. MCF7 cells were cultured on glass coverslips in phenol red-free DMEM containing 5% dextran-coated charcoal-stripped FCS, for 3 days before the addition of 10nM E2, 100 nM fulvestrant or 100 nM 4-hydroxytamoxifen, as appropriate. Cells were fixed with 4% paraformaldehyde for 10 minutes and permeabilsed with 0.2% Triton X-100 for a further 10

minutes. Non-specific staining was blocked with blocking solution (10% fetal calf serum/3% bovine serum albumin in PBS) for 1 hour, followed by incubation with γH2AX (05-636, Millipore), 53BP1 (ab36823, Abcam), PolII phospho-serine 2 (Ab5095, Abcam) or ER (HC-20, Santa Cruz) antibodies for 1 hour. Cells were washed three times in blocking buffer and the slides were incubated with Alexafluor 488 and 555 labeled antibodies (Invitrogen) for 1 hour. Cells were washed three times in blocking buffer and then DAPI and ToPro (Invitrogen) nuclear dyes were used to visualise nuclei. Cells were mounted with VectaShield (Vector labs) for confocal analysis. Images were acquired using the Carl Zeiss confocal microscope using the LSM 510 image browser. Images were analysed using Fuji Image J (NIH, USA) and CellProfiler (Broad Institute, USA) to quantitate number of foci per cell.

MCF-7 Human tumour xenografts. Female, 7-week-old, nu/nu-BALB/c athymic nude mice were purchased from Harlan Olac Ltd, UK. The study was undertaken under the auspices of a UK Home Office project license, using approved procedures. 0.72-mg 17β-estradiol 60-day release pellets (Innovative Research of America) were implanted subcutaneously prior to subcutaneous injection of MCF-7 cells (5×10^6) in a volume of 100µl into the flank of the animals. When tumours had reached 50-100 mm^3, they were randomly assigned into three groups, the vehicle, siControl and the siA3B treatment groups. Tumour-bearing mice were treated weekly by injection of 10 µM siRNA prepared with the atelogene *in vivo* siRNA transfection kit (Koken, Japan) directly into tumours. Tumour diameters were measured twice weekly with digital calipers and tumour volumes were calculated using the formula, length (L) X width^2 (W)/2. At the end of the experiment, tumours were excised and snap frozen. Tumours were divided in two and protein lysates were prepared by adding 400 µl RIPA buffer, including protease and phosphatase inhibitors and homogenizing in tubes containing 2.8 mm ceramic beads (Peqlab; cat no: 91-PCS-CK28) using a Precellys-24 homogeniser. Lysates were cleared by centrifugation for 5 min. at 13,000 rpm. RNA was prepared from the remaining halves of each tumour using the RNAeasy kit (Qiagen). Real-time RT-PCR and immunoblotting was performed as described above.

Chromatin immunoprecipitation (ChIP). Cells were cultured in estrogen-free medium for 3 days before addition of 100nM estrogen. ChIP assays were performed as described previously (Lai et al., 2013), using antibodies listed below, or the in-house rabbit A3B polyclonal antiserum. Control ChIP was performed by the addition of mouse immunoglobulins (IgG). For each ChIP, 10μg of antibody and 100μl of Protein A Dynalbeads (10002D; Invitrogen) were used.

Antibody	Catalogue No.	Supplier
ER	sc-543	Santa Cruz
DNA-PKcs	sc-9051	Santa Cruz
Ku70	sc-5309	Santa Cruz
BRG1	sc-10768	Santa Cruz
BRM	sc-6450	Santa Cruz
Histone H3	**Ab1791**	Abcam
H3K9ac	**Ab4441**	Abcam
H3K4me3	**Ab8580**	Abcam
UNG	ab23926	Abcam
γH2AX	05-636	Millipore
RNA PolII	05-623	Millipore

Real-time PCR (qPCR) was performed on the enriched DNA using primers listed below.

Gene		***Sequence***
TFF1 promoter proximal ER binding region (-340/-54 bp)	Forward	TATGAATCACTTCTGCAGTGAG
	Reverse	GAGCGTTAGATAACATTTGCC
TFF1 control (-685/-520 bp)	Forward	GTGATTCTCCTGACTTAACC
	Reverse	TGGCGCAGTGGCTCACGCTG
GREB1	Forward	GGGTGAAATGAAGTGGCATGTG
	Reverse	GAACAAAACAGAGCAAGGCCAAA
SCN2A1	Forward	AGAGTGGGCTTTGGTTTCCT
	Reverse	CCATCAGAAGCCAAAATGCT

RPL13A	Forward	CACGTTCTCTTGGACAGCAA
	Reverse	TTAAGGCTCCGCTGAGAGAG

Chromatin Immunoprecipitations and Solexa sequencing (ChIP-seq). ChIP DNA was amplified as described (Schmidt et al., 2009). Sequences were generated by the Illumina Hiseq 2000 genome analyser (using 50 bp reads), and aligned to the Human Reference Genome (assembly hg19, February 2009) using Bowtie 1.0. Enriched regions of the genome were identified by comparing the ChIP samples to an input sample using the MACS peak caller (Zhang et al., 2008), version 1.4. The number of reads obtained, the percentage of reads aligned and number of peaks called, are detailed in figs. S3D and S7A.

Motif analysis, heatmaps and genomic distributions of binding events. ChIP-seq data snapshots were generated using the Integrative Genome Viewer IGV 2.3 (www.broadinstitute.org/igv/). Motif analyses were performed using Cistrome (cistrome.org), applying the SeqPos motif tool (He et al., 2010). The genomic distributions of binding sites were analysed using the *cis*-regulatory element annotation system (CEAS) (Ji et al., 2006). The genes closest to the binding site on both strands were analysed. If the binding region is within a gene, CEAS software indicates whether it is in a 5′UTR, a 3′UTR, a coding exon, or an intron. Promoter is defined as a region 0-3 kb upstream from RefSeq 5′ start. If a binding site is >3 kb away from the RefSeq transcription start site, it is considered distal intergenic. For integration with gene expression data, binding events were considered proximal when identified in a gene body or within 20kb upstream of the transcription start site. Heatmaps were plotted using the CHASE software package (http://chase.cs.univie.ac.at/overview).

Biotin labeling of DNA strand breaks. Biotin labelling of DNA strand breaks was performed as described (Ju et al., 2006). Estrogen was added to MCF-7 cells cultured for 72 hours in estrogen-free medium. Cells were fixed with Streck Tissue Fixative (STF, Streck Laboratories) for 10 minutes at 37°C, scraped from plates, washed twice with ice-cold PBS and centrifuged at 2,000

rpm for 5 minutes. Cell pellets were re-suspended in Buffer A (0.25% Triton X-100, 10mM EDTA, 10mM HEPES [pH6.5]) and placed on a rotating wheel for 10 minutes. Cells were centrifuged at 2,000 rpm for 5 minutes and pellets were re-suspended in Buffer B (200mM NaCl, 1mM EDTA, 10mM HEPES pH6.5), and permeabilized with Buffer C (100mM Tris-HCl pH7.4, 50mM EDTA, 1% Triton X-100) for one hour at 4°C. The nuclear pellets thus prepared were sequentially washed with ice-cold PBS, deionized water and 1x terminal deoxynucleotide transferase (TdT; Promega) reaction Buffer. To label DNA breaks the nuclei were incubated with biotin-16-dUTP (Roche, UK) and TdT for 1 hour at 37°C. Residual biotin-16-dUTP was removed by washing with Buffer D (100mM Tris-HCl pH7.4, 150mM NaCl). Nuclei were fixed by the addition of 1% formaldehyde and biotin-labelled ends were immunopurified using an anti-biotin antibody (Sigma-Aldrich, UK). DNA was prepared as for the ChIP assay. Real-time PCR analysis was performed using primers listed below.

Primer		***PCR Primer Sequence***	***PCR Product (hg19)***	***ERE Location***	***ERE sequence***
TFF1 B/C (-391/-240)	F	CCCGTGAGCCACTGTTGT	chr21:43,787,017 - 43,787,210	chr21:43,787,036 - 43,787,050	AGGTCACGGTGGCCA
	R	ATGGGAGTCTCCTCCAACCT			
TFF1 A/C (-567/-240)	F	TTAAGTGATCCGCCTGCTTT	chr21:43,786,884-43,787,210	as above	
	R	ATGGGAGTCTCCTCCAACCT			
GREB1	F	GAAGGGCAGAGCTGATAACG	chr2:11,672,533 - 11,672,663	chr2:11,672,659 - 11,672, 673	GGGTCATTCTGACCT
	R	GACCCAGTTGCCACACTTTT			
EGR3	F	GCCATCTGAGGCTTGAAAAG	chr8:22,592,462 - 22,592,611	chr8:22,592,668 - 22,592,682	AGGTCATGGTATAAG
	R	CCCATCTCACCAGCTACCAT			
PDZK1	F	AGGCCCAGCAAAGACAAATG	chr1:145,726,657 - 145,726,743	chr1:145,726,711 - 145,726,725	AGGTCACCCAGTCCT
	R	AAACCACAGGCTGAGGACTG			
SCN2A1	F	AGAGTGGGCTTTGGTTTCCT	chr2:166,094,638 - 166,094,813	Not applicable	
	R	CCATCAGAAGCCAAAATGCT			

3D-PCR, Cloning and Sequencing

PCR primer details for 3D-PCR are given below.

TFF1		***Sequence***
1st round PCR	Forward	TTAAGTGATCCGCCTGCTTT
	Reverse	AGCCCCGGATTTTATAGGG

2nd round PCR	Forward	TGATCCGCCTGCTTTGGCCT
	Reverse	CCGGATTTTATAGGGCAGGC
PDZK1		***Sequence***
1st round PCR	Forward	GCAACCTCTGCCTCTCAATC
	Reverse	GTCCCAGGCTTCCTTCTTCT
2nd round PCR	Forward	AGACAAGGTTTTGCCATGTG
	Reverse	TCTTCCCCTTCACACACTCA

Analysis of breast cancer gene expression microarray data sets

A trancriptomic breast cancer database of publicly available microarrays was established as described previously (Gyorffy and Schafer, 2009). The entire database contains 5,935 breast cancer patients. In these patients, 4,659 have survival information, with the average follow-up for relapse-free survival = 68.7 months. 76% of the patients are ER-positive and 42% are lymph node positive. The gene chip files were MAS5 normalized using the affy Bioconductor library. Illumina microarrays generated by the Metabric consortium (Curtis et al., 2012) were processed in the R statistical environment (http://www.r-project.org). The database includes 1,988 patients, the average overall survival is 8.07 years, 76% of the patients are ER-positive and 47.3% are lymph node positive. The Illumina microarray files were processed using the beadarray package, the illuminaHumanv3 database for annotation and quantile normalization via the preprocessCore package (http://www.bioconductor.org).

Kaplan-Meier survival plots and the hazard ratios with 95% confidence intervals and logrank P-values were calculated and plotted in R, as previously described (Gyorffy et al., 2013). Statistical significance was set at $p<0.01$. In addition, Cox proportional hazard regression was performed to compare the association between gene expression, clinical variables and survival using WinSTAT 2013 for Microsoft Excel (Robert K. Fitch Software, Germany).

Supplemental Figure Legends

Figure S1. APOBEC3B expression is associated with poor patient survival in estrogen receptor-positive breast cancer, related to figure 1. **(A, B)** Kaplan-Meier plots of breast cancer survival for ER-positive breast cancer patients (Fig. 1A), ER-negative patients, or all patients, according to A3B expression in the METABRIC data set. **(C)** Multivariate Cox regression analysis (METABRIC), including A3B expression, nodal status, grade and tumor size. **(D-F)** KM plots of a database comprising public Affymetrix microarray data show association between A3B expression and poor outcome in ER-positive breast cancer. **(G)** RNA prepared from breast cancer cell lines was assessed for APOBEC gene expression by quantitative RT-PCR. Boxplots show expression of all family members. APOBEC3B is the most highly and widely expressed family member, APOBEC3C being the other family member expressed widely in breast cancer cells. Expression of all other family members is 10-1000 times lower than that of APOBEC3B in the vast majority of lines. **(H-R)** Shown is the mRNA expression of APOBEC family members in each breast cancer cell line, which are summarized in (G). The order in which the cell lines are shown is based on the APOBEC3B expression profile, ranging from lines with the least APOBEC3B expression (left) to highest APOBEC3B expression (right) (H).

Figure S2. APOBEC3B regulates ER target gene expression in MCF-7 cells, related to figure 1. **(A)** Hormone-depleted MCF-7 cells were transfected with two independent siRNAs for A3B, or with a control siRNA. Cell growth in the presence of 10 nM estrogen or vehicle control was measured using the sulphorhodamine B (SRB) assay and is shown relative to growth on day of transfection (day 0; n=5). **(B)** MCF-7 cells were transfected with A3B siRNAs. Forty-eight hours later, estrogen (10 nM) was added and RNA prepared a further 12 hours later. Quantitative RT-PCR was undertaken and gene expression was corrected for GAPDH levels and is shown for the estrogen treated samples, relative to expression of each gene in the vehicle control (n=3). **(C)** Hormone-depleted MCF-7 cells were transfected with two independent A3B siRNAs or with control siRNA. Estrogen (10 nM) was added

after 48 hours and protein lysates prepared a further 12 hours later were immunoblotted. ß-actin served as a loading control. **(D)** SkBr3 cells, which are null for A3B, were transfected with A3B siRNAs. There was no effect on SkBr3 cell growth (n=5). **(E)** MDA-MB-231 ER-negative breast cancer cells that express A3B were treated with A3B siRNAs and growth was assessed over 4 days (n=4) The bar chart shows real-time RT-PCR for A3B following siRNA transfection (n=3). **(F)** Hormone-depleted MCF-7 cells were transfected with HA-A3B. After 24 hours, the cells were transfected with A3B siRNAs targeting the coding region (ORF), or with a siRNA that targets the A3B 3'-untranslated region (3'UTR). A further 24 hours later, estrogen (10 nM) was added for 12 hours. Real-time RT-PCR results are shown for A3B and ER target genes, TFF1, PDZK1 and PGR (n=3). *=p<0.01 for a comparison of siControl transfected cells with siA3B transfected cells, comparisons being within the vector or HA-A3B transfected samples. # denotes significant differences (p<0.01) for siControl, siA3B (3'UTR), siA3B#1 or siA3B#2 for equivalent vector and HA-A3B transfections. **(G)** Immunoblotting for A3B and HA tags demonstrates expression of ectopic and endogenous A3B.

Figure S3. Recruitment of APOBEC3B to chromatin is estrogen receptor dependent, related to figure 2. (A) ChIP assay for the ER binding region of the GREB1 gene, using lysates prepared from hormone-depleted MCF-7 cells treated with estrogen for 45 minutes, as for the TFF1 gene in Fig. 2C. **(B)** Details of A3B ChIP-sequencing runs are shown. **(C)** Genomic distribution of binding regions from A3B ChIP-seq in estrogen-treated cells. **(D)** MCF-7 cells were treated with Fulvestrant (FUL) for 24 hours. Estrogen was added 45 minutes prior to ChIP lysate preparation. ChIP was performed for the ER binding region of the PDZK1 gene, as for the TFF1 gene in Fig. 2E. Immunoblotting confirms that fulvestrant treatment downregulates ER. Note that fulvestrant treatment does not influence A3B levels. **(E)** ChIP was performed from MCF-7 lysates prepared following transfection with siA3B or siControl.

Figure S4. APOBEC3B mediates C-to-T transitions in the PDZK1 ER binding region in T47D cells, related to figure 3.

(A) genome browser snapshot of A3B, γH2AX and ER ChIP-seq. **(B)** Real-time RT-PCR was carried out using RNA prepared from T47D cells transfected with siControl or siA3B (n=3). **(C)** agarose gel analysis of 3D-PCR amplicons from T47D cells transfected with control or A3B siRNAs using primers flanking the PDZK1 ER binding region. Asterisks denote $p<0.001$. **(D)** mutation analysis of 3D-PCR amplicons was carried out by cloning and sequencing of PCR products (n>45). C-to-T changes were found in 19/56 (33.3%) of clones from siControl cells, compared with 4/47 (8.5%) of clones from siA3B transfected T47D cells. **(E)** Positions of C-to-T changes identified from cloning of 3D-PCR amplicons are depicted as blue dots. Dots above the red bar show C-to-T changes on the coding strand, those below the red bar are C-to-T changes on the template strand. The region sequenced maps between bp 145,726,380 and 145,726,873 (hg19). **(F)** Hormone-depleted T47D cells were transfected with UNG siRNA, or with siControl. Cells were treated with 10 nM estrogen after 48 hours and RNA prepared after a further 12 hours. Real-time PCR analysis for three replicates is shown. **(G)** Hormone-depleted T47D cells were transfected with myc-tagged UGI. Estrogen was added after 24 hours. RNA was prepared after a further 12 hours and protein lysates after 24 hours estrogen treatment. Real-time PCR is shown for n=3. * = $p<0.001$.

Figure S5. Estrogen treatment results in rapid and transient induction of γH2AX, related to figure 4. **(A)** Hormone-depleted MCF-7 cells were treated for 10 minutes with estrogen, diethylstilbesterol (DES) or the selective estrogen receptor-α agonist PPT. Cells were fixed and immunostained with antibodies for γH2AX and nuclei were visualised by staining with TOPRO DNA stain. **(B)** The number of γH2AX foci per cell were quantified using Cell Profiler 2.0. The bar chart shows the mean number of γH2AX foci in 100 cells from five replicates (total n=500). **(C)** Hormone-depleted MCF-7 cells were treated with vehicle, estrogen (10 nM), 4-hydroxytamoxifen (OHT; 100 nM) or Fulvestrant (FUL; 100 nM) for 10 minutes. For the H_2O_2 (10 mM) treatment, cells were fixed 45 minutes after the addition of H_2O_2. **(D)** hormone-depleted MCF-7 cells were transfected with A3B or ER siRNAs and immunostained for

γH2AX following 10 min estrogen treatment. **(E, F)** Estrogen, OHT or fulvestrant were added to T47D cells. The cells were fixed after 10 minutes and immunostained for γH2AX. The bar chart shows quantification for γH2AX foci number per cell for 100 cells (n=5 for a total of 500 cells). **(G)** Hormone-depleted MCF-7 cells were pre-treated with vehicle, a DNA-PK inhibitor (Nu7441; 5μM) or ATM inhibitor (KU55933; 5μM), for 1 hour and then vehicle or estrogen for 10 minutes. The bar chart shows quantification of a total of 500 cells from 5 replicates. **(H)** Estrogen-treated MCF-7 cells were immunostained for γH2AX (green) and 53BP1 (red). Fluorescence intensity overlap was assessed using the Carl Zeiss LSM 510 software to demonstrate co-localisation. **(I)** hormone-depleted MCF-7 cells were transfected with an UNG siRNA and immunostained for γH2AX following 10 min estrogen treatment.

Figure S6. Ectopic expression of the estrogen receptor in MDA-MB-231 cells facilitates APOBEC3B-dependent γH2AX induction and promotes expression of ER target genes, related to figure 4. (A) Treatment of the ER-negative MDA-MB-231 cells cultured in hormone-depleted culture medium, with estrogen for 10 minutes did not induce γH2AX. By contrast, estrogen treatment of two independent MDA-MB-231 derived lines stably expressing ER, caused γH2AX induction. **(B)** γH2AX induction was blocked by siA3B transfection in these lines. **(C)** Immunoblotting for A3B and ER following siA3B transfection shows A3B knockdown in MDA-MB-231 and ER expressing clones. **(D)** ER expression in MDA-MB-231 stimulates expression of ER target genes in an estrogen-dependent manner. A3B knockdown inhibits expression of these ER target genes (n=3; *$p<0.001$; #$p<0.05$).

Figure S7. γH2AX ChIP-seq shows concordance with A3B and ER binding regions related to figure 4. (A) Details of γH2AX ChIP-sequencing runs. **(B)** Correlation coefficient values on the genome scale for γH2AX, A3B and ER using raw (Wig) data. ChIP-seq reads were normalized (wig file) and binned in windows of 100 kb where the average score was calculated. The data were used to calculate genome wide correlation using a Spearman's

correlation score. **(C)** Heat map showing clustered binding signal for regions enriched in γH2AX for all treatment conditions. The window represents ±2.5 kb regions from the centre of the γH2AX regions. **(D)** Public data for MCF-7 cells from GEO was downloaded and aligned to hg19 using bwa software with default parameters. The MCF-7 datasets for p300 (Zwart et al., 2011), H4K12ac (Nagarajan et al., 2015), H3K4me1 (Theodorou et al., 2013) and BRD4 (Nagarajan et al., 2015) have been described, as has the GRO-seq (Hah et al., 2013). Encode Project data for H3K27ac and H3K4me3 were also used (http://encodeproject.org). Consensus ER peak list was based on the peaks present in at least two out of three MCF7 ER ChIP-seq replicates from Ross-Innes *et al*. (Ross-Innes et al., 2012) and contained 58661 peaks. Promoter-associated ER peaks were defined as peaks that overlap 3000 bp promoter regions (3000 bp upstream of TSS). All the other ER peaks were considered as enhancer-associated peaks. Peaks were sorted based on ER intensity. Heatmaps were visualized using seqMiner v1.3 software.

Table S1. DNA Motif enrichment analysis, related to figures 2 and 4.

References

Bogerd, H.P., Wiegand, H.L., Doehle, B.P., and Cullen, B.R. (2007). The intrinsic antiretroviral factor APOBEC3B contains two enzymatically active cytidine deaminase domains. Virology *364*, 486-493.

Curtis, C., Shah, S.P., Chin, S.F., Turashvili, G., Rueda, O.M., Dunning, M.J., Speed, D., Lynch, A.G., Samarajiwa, S., Yuan, Y., *et al.* (2012). The genomic and transcriptomic architecture of 2,000 breast tumours reveals novel subgroups. Nature *486*, 346-352.

Gyorffy, B., and Schafer, R. (2009). Meta-analysis of gene expression profiles related to relapse-free survival in 1,079 breast cancer patients. Breast cancer research and treatment *118*, 433-441.

Gyorffy, B., Surowiak, P., Budczies, J., and Lanczky, A. (2013). Online survival analysis software to assess the prognostic value of biomarkers using transcriptomic data in non-small-cell lung cancer. PloS one *8*, e82241.

Hah, N., Murakami, S., Nagari, A., Danko, C. G., and Kraus, W. L. (2013). Enhancer transcripts mark active estrogen receptor binding sites. Genome research *23*, 1210-1223.

He, H.H., Meyer, C.A., Shin, H., Bailey, S.T., Wei, G., Wang, Q., Zhang, Y., Xu, K., Ni, M., Lupien, M.*, et al.* (2010). Nucleosome dynamics define transcriptional enhancers. Nature genetics *42*, 343-347.

Ji, X., Li, W., Song, J., Wei, L., and Liu, X.S. (2006). CEAS: cis-regulatory element annotation system. Nucleic acids research *34*, W551-554.

Ju, B.G., Lunyak, V.V., Perissi, V., Garcia-Bassets, I., Rose, D.W., Glass, C.K., and Rosenfeld, M.G. (2006). A topoisomerase IIbeta-mediated dsDNA break required for regulated transcription. Science *312*, 1798-1802.

Lai, C.F., Flach, K.D., Alexi, X., Fox, S.P., Ottaviani, S., Thiruchelvam, P.T., Kyle, F.J., Thomas, R.S., Launchbury, R., Hua, H.*, et al.* (2013). Co-regulated gene expression by oestrogen receptor alpha and liver receptor homolog-1 is a feature of the oestrogen response in breast cancer cells. Nucleic acids research *41*, 10228-10240.

Lopez-Garcia, J., Periyasamy, M., Thomas, R.S., Christian, M., Leao, M., Jat, P., Kindle, K.B., Heery, D.M., Parker, M.G., Buluwela, L.*, et al.* (2006). ZNF366 is an estrogen receptor corepressor that acts through CtBP and histone deacetylases. Nucleic acids research *34*, 6126-6136.

Nagarajan, S., Benito, E., Fischer, A., and Johnsen, S. A. (2015). H4K12ac is regulated by estrogen receptor-alpha and is associated with BRD4 function and inducible transcription. Oncotarget *6*, 7305-7317.

Reddy, S.M., Williams, M., and Cohen, J.I. (1998). Expression of a uracil DNA glycosylase (UNG) inhibitor in mammalian cells: varicella-zoster virus can replicate in vitro in the absence of detectable UNG activity. Virology *251*, 393-401.

Ross-Innes, C. S., Stark, R., Teschendorff, A. E., Holmes, K. A., Ali, H. R., Dunning, M. J., Brown, G. D., Gojis, O., Ellis, I. O., Green, A. R.*, et al.* (2012). Differential oestrogen receptor binding is associated with clinical outcome in breast cancer. Nature *481*, 389-393.

Schmidt, D., Wilson, M.D., Spyrou, C., Brown, G.D., Hadfield, J., and Odom, D.T. (2009). ChIP-seq: using high-throughput sequencing to discover protein-DNA interactions. Methods *48*, 240-248.

Theodorou, V., Stark, R., Menon, S., and Carroll, J. S. (2013). GATA3 acts upstream of FOXA1 in mediating ESR1 binding by shaping enhancer accessibility. Genome research *23*, 12-22.

Thiruchelvam, P.T., Lai, C.F., Hua, H., Thomas, R.S., Hurtado, A., Hudson, W., Bayly, A.R., Kyle, F.J., Periyasamy, M., Photiou, A.*, et al.* (2011). The liver receptor homolog-1 regulates estrogen receptor expression in breast cancer cells. Breast cancer research and treatment *127*, 385-396.

Zhang, Y., Liu, T., Meyer, C.A., Eeckhoute, J., Johnson, D.S., Bernstein, B.E., Nusbaum, C., Myers, R.M., Brown, M., Li, W.*, et al.* (2008). Model-based analysis of ChIP-Seq (MACS). Genome biology *9*, R137.

Zwart, W., Theodorou, V., Kok, M., Canisius, S., Linn, S., and Carroll, J. S. (2011). Oestrogen receptor-co-factor-chromatin specificity in the transcriptional regulation of breast cancer. The EMBO journal *30*, 4764-4776.

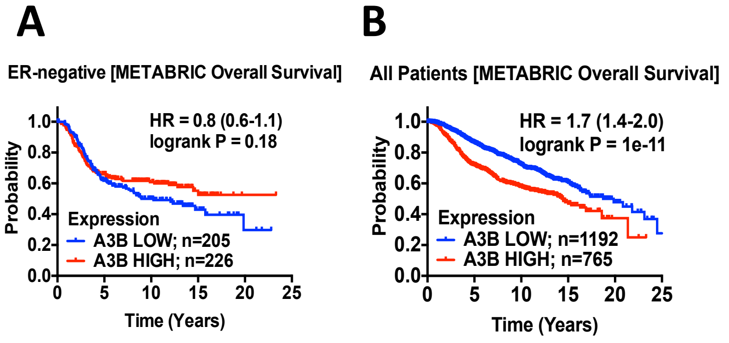

C

METABRIC	p-value	HR	Conf. (±)
APOBEC3B	$1.27x10^{-6}$	1.59	0.19
Lymph Node Status	$4.49x10^{-7}$	1.92	0.20
Tumor Grade	$4.00x10^{-2}$	1.18	0.16
Tumor Size	$5.05x10^{-7}$	1.02	0.00

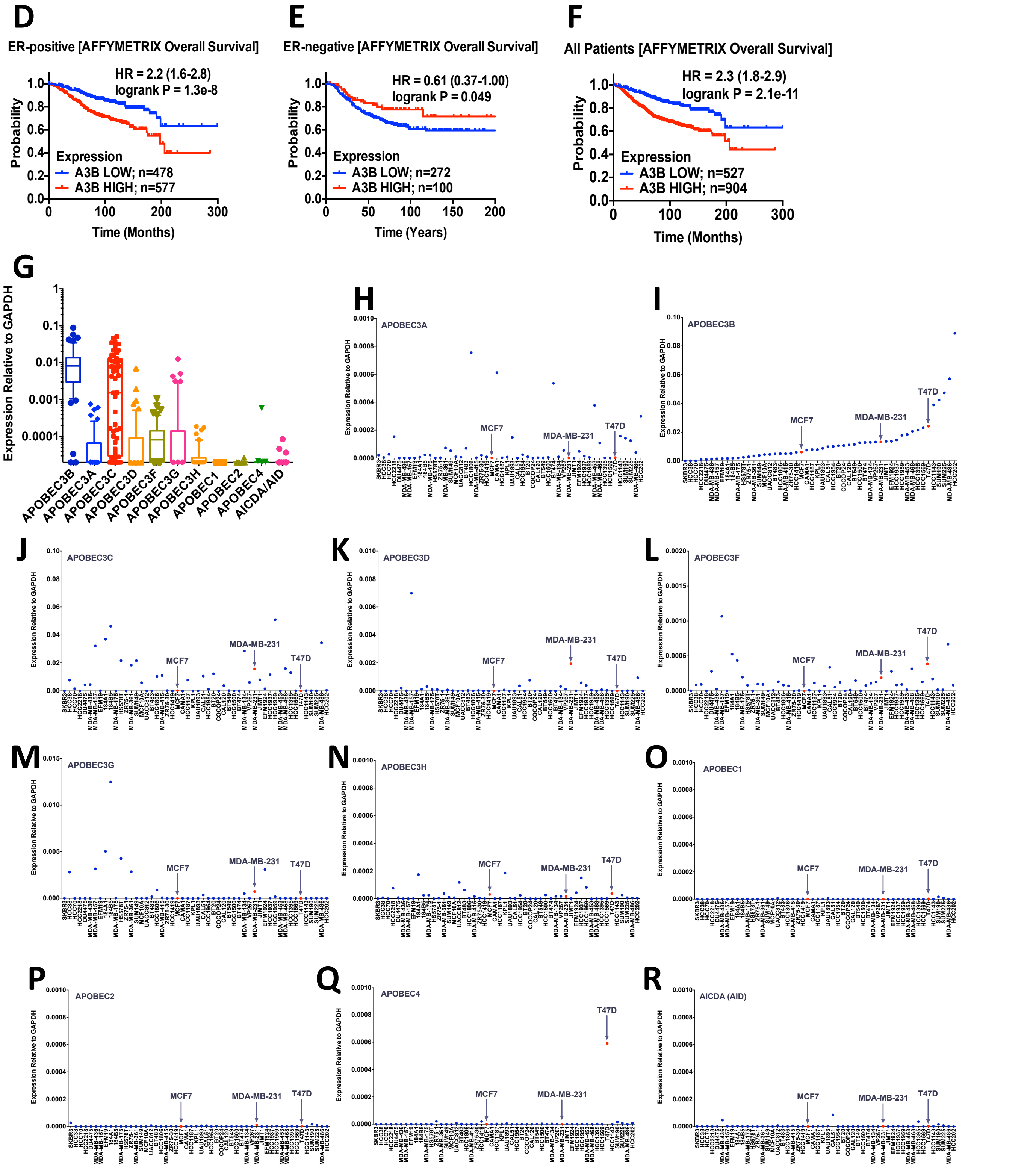

Figure S1 Periyasamy *et al*

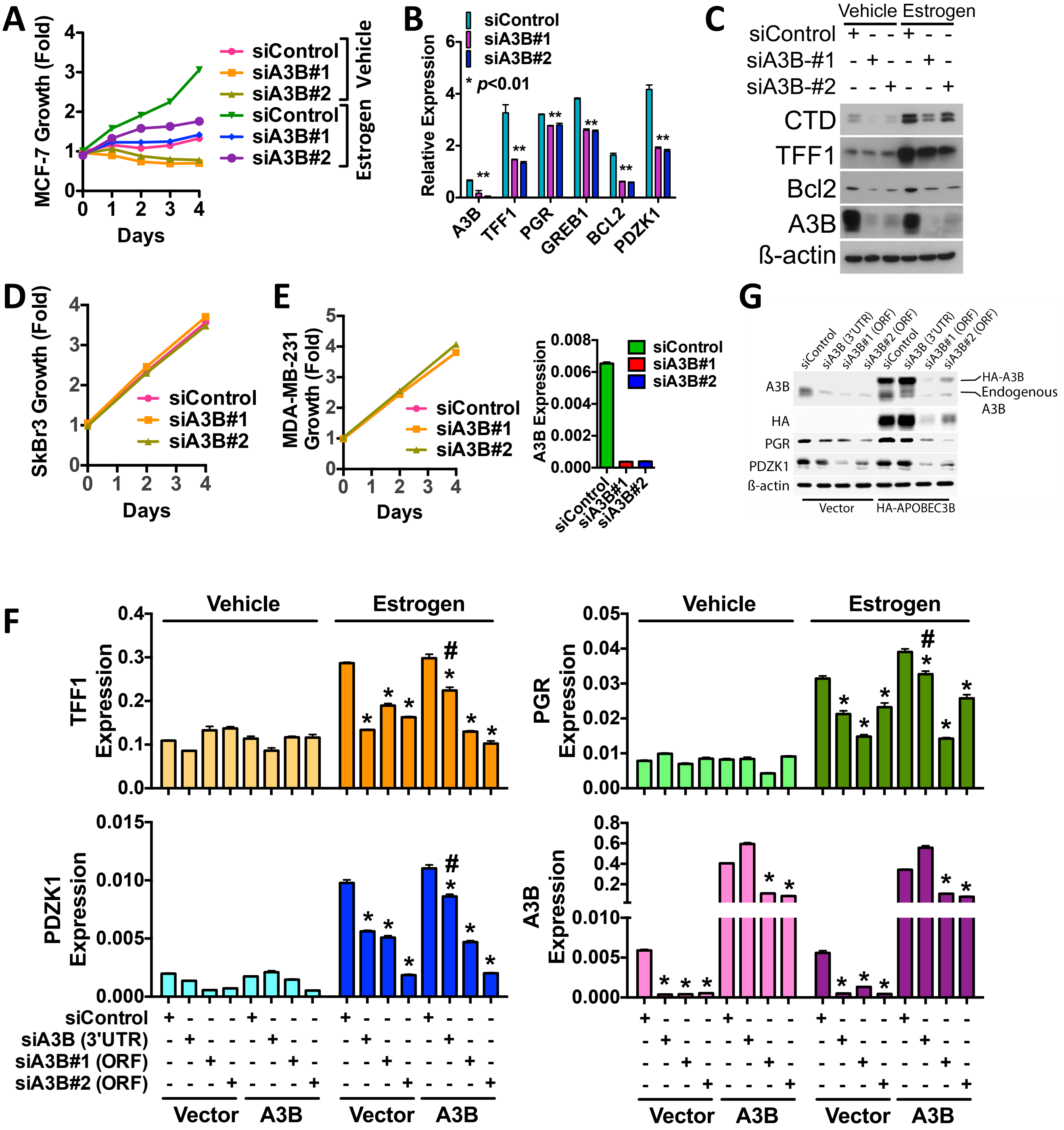

Figure S2 Periyasamy *et al*

A

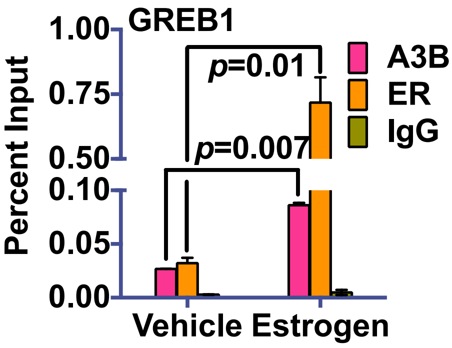

B

A3B ChIP-seq	Total Reads	Aligned Reads	% Aligned
Input Vehicle	18,032,282	15,180,049	84.2
A3B Vehicle	25,996,991	25,237,544	97.1
A3B Estrogen	32,913,006	31,895,248	96.9

C

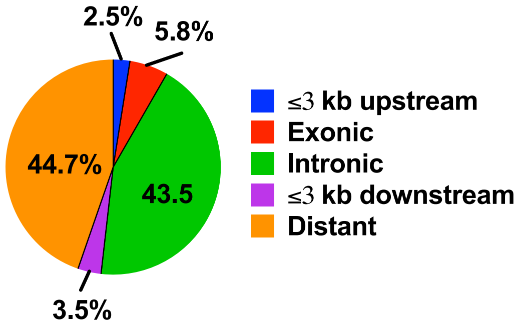

D

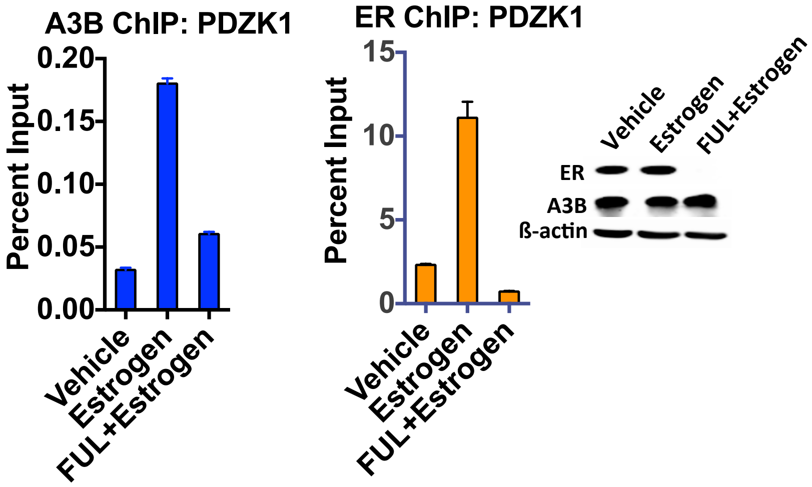

E

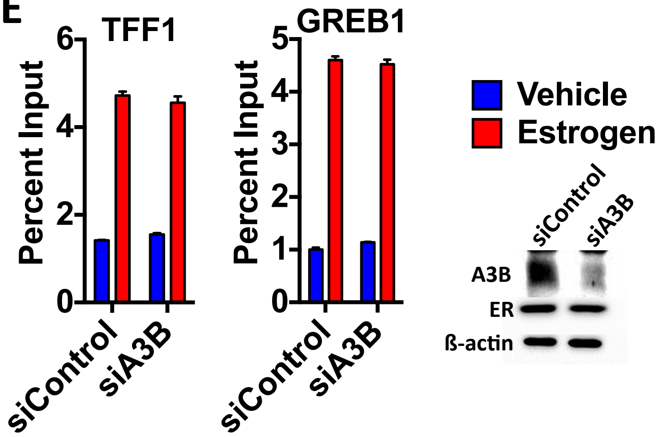

Figure S3 Periyasamy *et al*

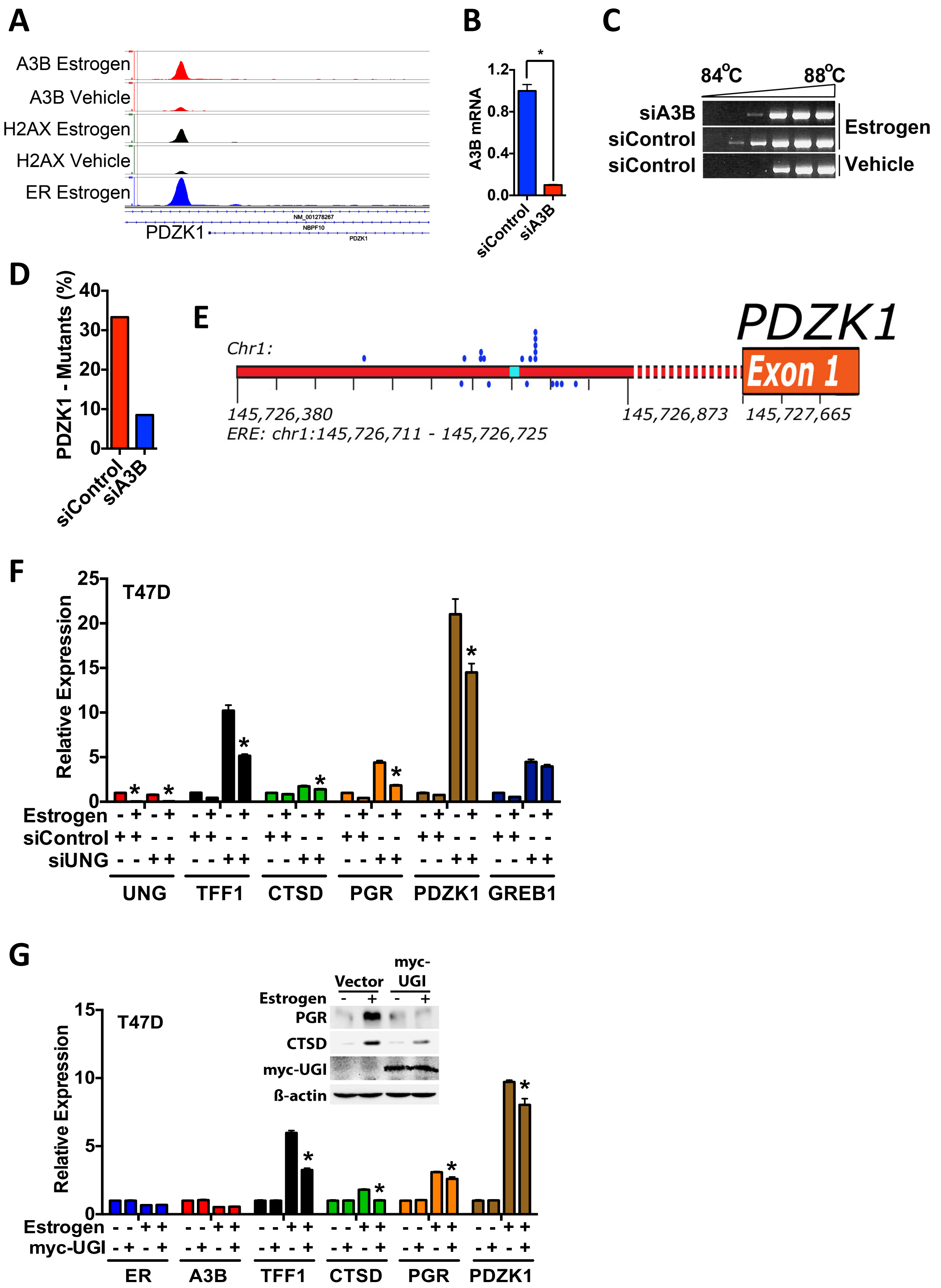

Figure S4 Periyasamy *et al*

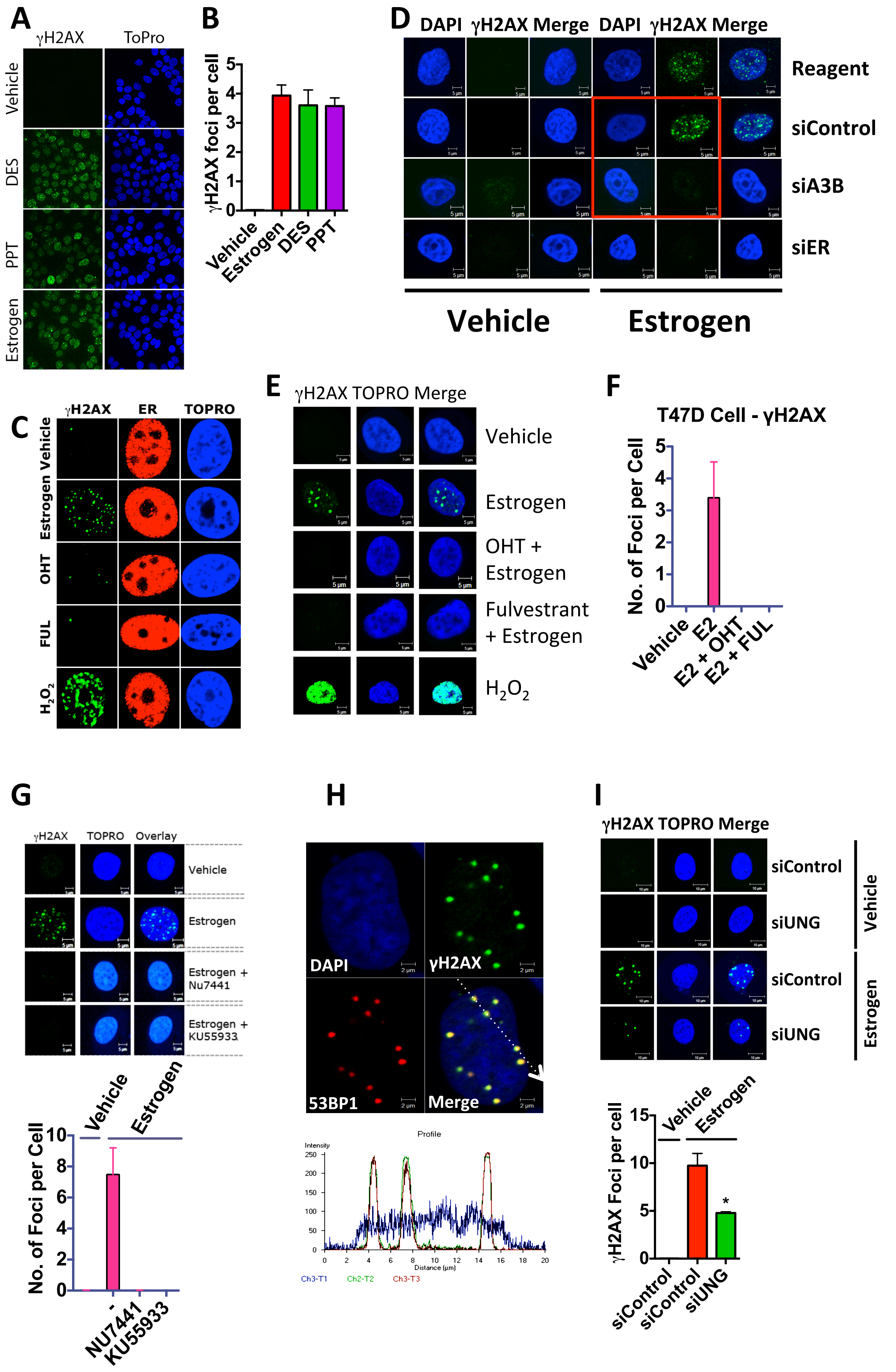

Figure S5 Periyasamy *et al*

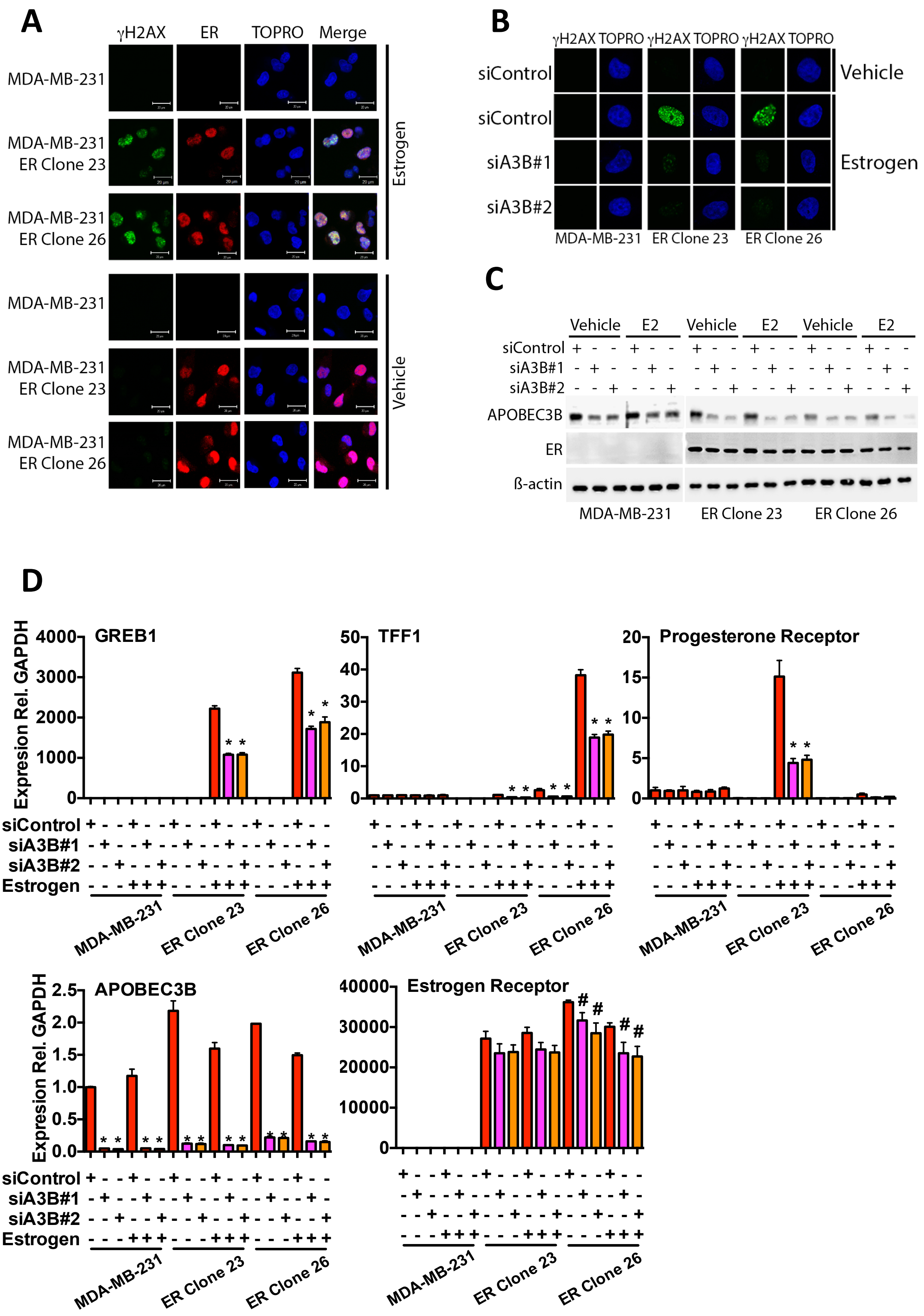

Figure S6 Periyasamy *et al*

A

γH2AX ChIP-seq	Total Reads	Aligned Reads	% Aligned
Vehicle	5,748,915	5,626,134	97.9
Estrogen	13,046,826	12,711,728	97.4
H_2O_2	7,057,147	6,944,363	98.4
H_2O_2/Estrogen	12,937,543	12,548,040	97.0

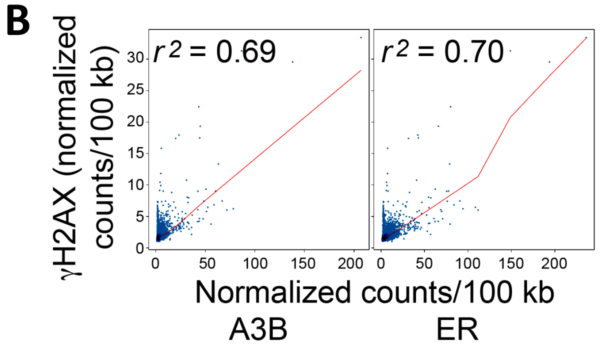

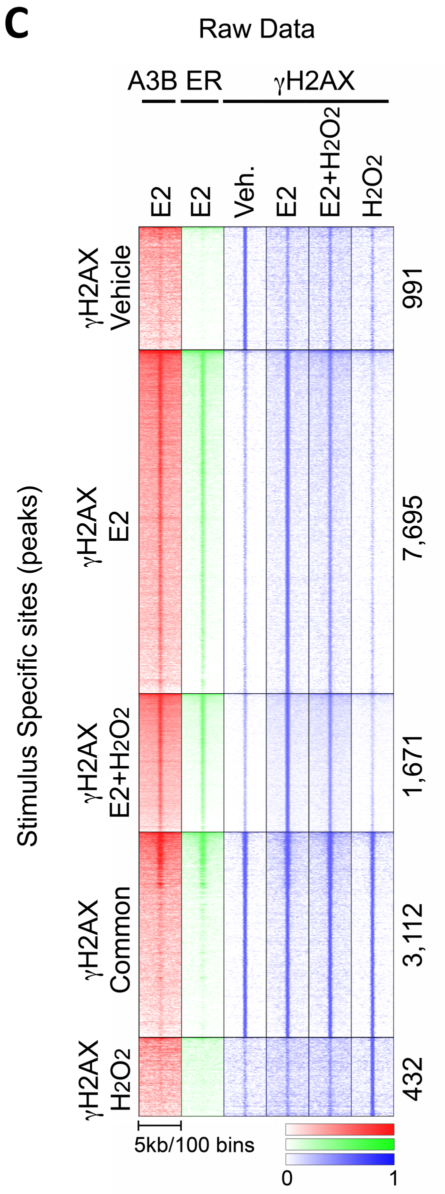

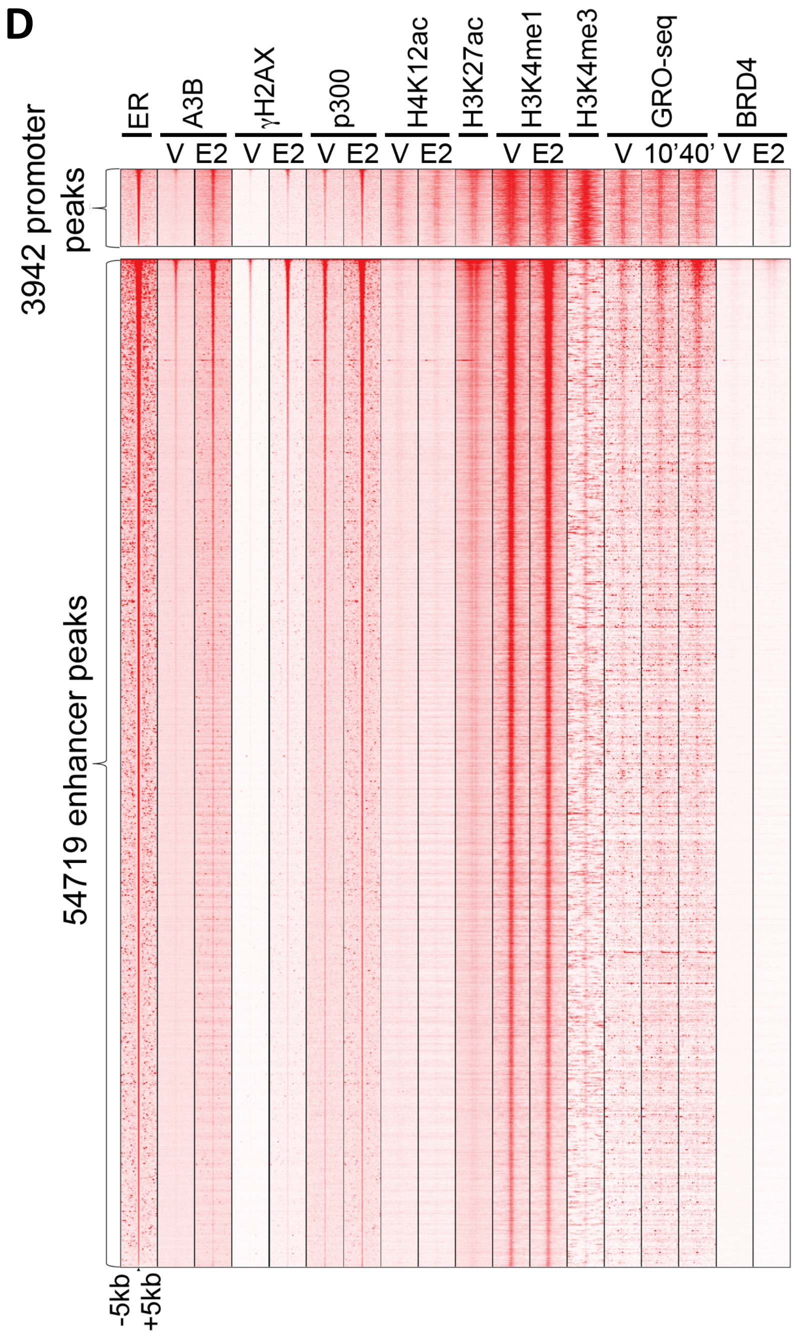

Figure S7 Periyasamy *et al*
